# Ultrafast multi-level control of sub-50 nm skyrmions in a Pd-intercalated van der Waals magnet

**DOI:** 10.1038/s41467-026-75870-4

**Published:** 2026-07-21

**Authors:** Huai Zhang, Bei Ding, Bo Zhao, Ke Pei, Yue Hu, Runhang Zhang, Rui Su, Minghao Zheng, Zefang Li, Qing Zhou, Guoping Zhao, Xingsen Gao, Yangfan Hu, Xue Jiang, Jijun Zhao, Renchao Che, Xuewen Fu, Zhipeng Hou, Junming Liu

**Affiliations:** 1 Guangdong Provincial Key Laboratory of Optical Information Materials and Technology, Institute for Advanced Materials, South China Academy of Advanced Optoelectronics, South China Normal University, Guangzhou, China; 2 Guangdong Basic Research Center of Excellence for Structure and Fundamental Interactions of Matter, Guangdong Provincial Key Laboratory of Quantum Engineering and Quantum Materials, School of Physics, South China Normal University, Guangzhou, China; 3 Key Laboratory of Material Modification by Laser, Ion and Electron Beams, (Dalian University of Technology), Ministry of Education, Dalian, China; 4 College of Smart Materials and Future Energy, Fudan University, Shanghai, China; 5 Ultrafast Electron Microscopy Laboratory, The MOE Key Laboratory of Weak-Light Nonlinear Photonics, School of Physics, Nankai University, Tianjin, China; 6 School of Materials Science and Engineering & Research Institute of Interdisciplinary Science, Dongguan University of Technology, Dongguan, China; 7 School of Materials and Microelectronics, Wuhan University of Technology, Wuhan, China; 8 Guangdong Provincial Key Laboratory of Rare Earth Development and Application, Guangdong Academy of Sciences, Guangzhou, China; 9 College of Physics and Electronic Engineering, Sichuan Normal University, Chengdu, China; 10 Academy for Advanced Interdisciplinary Studies, Nankai University, Tianjin, China; 11 Laboratory of Solid State Microstructures and Innovation Center of Advanced Microstructures, Nanjing University, Nanjing, China

**Keywords:** Spintronics, Magnetic properties and materials

## Abstract

Achieving ultrafast, multi-level control of nanoscale skyrmions offers a transformative route for advancing van der Waals spintronics towards high-speed, scalable neuromorphic computing applications. However, progress has been impeded by the relatively large skyrmion size (~100 nm) in existing van der Waals magnets and the lack of efficient control strategies. Here, we simultaneously address both challenges by combining atomic intercalation with femtosecond laser manipulation. Through Pd atomic intercalation into the van der Waals magnet Fe_3-δ_GaTe_2_, we realize magnetic field-stabilized skyrmions with an average diameter of ~43 nm at room temperature, the smallest skyrmions reported in the van der Waals magnets to date. Mechanism analysis reveals that this size reduction arises from enhanced Dzyaloshinskii-Moriya interaction and suppressed Heisenberg exchange coupling. On this tailored platform, we further demonstrate femtosecond laser-induced ultrafast generation of 43 nm skyrmions with an ultra-low energy consumption of 0.6 pJ per skyrmion. Most importantly, by tuning the laser pulse number, we achieve deterministic, multi-level modulation of skyrmion density, enabling skyrmion-based optical neuromorphic computing with a simulated training accuracy of ~91%.

## Abstract (web summary)

Huai Zhang et al. demonstrate that Pd intercalation in Fe_3-δ_GaTe_2_ stabilizes 43 nm room-temperature skyrmions. On this platform, femtosecond laser pulses enable multi level skyrmion generation for neuromorphic computing.

## Introduction

The emergence of van der Waals (vdW) magnets has opened new frontiers for spintronics, owing to their distinctive properties, such as robust long-range ferromagnetism down to the monolayer limit^1,2^, inherent stacking flexibility for constructing multifunctional architectures^3–5^, and atomic-level structural tunability for precise control over magnetic interactions and emergent spin textures^6–8^. Building on these advantages, the design of vdW spintronic devices has primarily relied on electrical means rooted in complementary metal-oxide-semiconductor (CMOS) architectures^7,9–11^. However, their information writing speed is typically constrained to the nanosecond regime, posing an inherent bottleneck that falls short of the demands of high-throughput computing. In contrast, ultrafast laser excitation, via mechanisms such as the inverse Faraday effect^12^ or ultrafast optical-thermal processes^13–21^, enables magnetization manipulation on the picosecond timescale, offering a significant speed advantage. Moreover, the contactless nature of optical control eliminates the need for complex wiring and gate electrodes inherent to the CMOS architectures^2,22,23^, greatly simplifying device integration, which is a particularly crucial advantage when scaling down to the two-dimensional limit^24^. Therefore, optical control represents a transformative pathway for advancing vdW spintronic technologies.

To realize optically driven spintronic functionalities in vdW magnets, a central requirement is the ability to precisely manipulate their spin textures under ultrafast laser excitation. Among these spin textures^19,25–41^, magnetic skyrmions, topologically protected swirling spin configurations, stand out as particularly promising candidates^25–38^. Compared to conventional collinear domain structures, they exhibit not only enhanced structure stability^42,43^, but also possess additional topological degrees of freedom, such as topological charge, polarity, and chirality^42,43^, enabling richer logic operations beyond binary encoding^44–47^. Moreover, their particle-like feature allows the number of skyrmions to be treated as a discrete, countable variable, facilitating multi-level control^48–52^. These unique attributes make skyrmions well-suited for multi-level memory and neuron-like functionalities, offering a pathway beyond traditional binary memory architectures towards brain-inspired computing paradigms^48–51^. In addition, the pronounced topological magneto-optical Kerr effect of skyrmions allows non-contact fast optical readout^22,23^, further underscoring their potential for realizing optical neuromorphic devices. However, despite these advantages, two key challenges remain unresolved for advancing such applications. First, skyrmions in existing vdW magnets typically exhibit a relatively large diameter (~100 nm at room temperature)^25–38^, posing a critical bottleneck for device miniaturization. Substantially reducing their size while maintaining a high skyrmion density is essential for enhancing storage density and scalability. Second, laser-induced deterministic, multi-level skyrmion modulation, a critical requirement for neuromorphic computing, has yet to be realized^13–15,17,18,20^.

In this work, we present an integrated materials-to-control strategy that enables ultrafast, multi-level control of sub-50 nm skyrmions in an intercalated vdW magnet using femtosecond (fs) laser pulses. By introducing Pd atomic intercalation into the vdW magnet Fe_3-δ_GaTe_2_, we first realize magnetic field-stabilized skyrmions with a minimum average diameter of ~43 nm at room temperature, representing the smallest skyrmions reported in the vdW magnets to date^25–38^. A comprehensive investigation reveals that this size reduction stems from enhanced Dzyaloshinskii-Moriya interaction (DMI) and suppressed Heisenberg exchange coupling. On this tailored vdW material platform, we further demonstrate the fs laser-induced ultrafast generation of ~43 nm skyrmions with a record-high density of ~101 μm^−2^ and an ultra-low energy consumption of 0.6 pJ per skyrmion. Crucially, by tuning the number of laser pulses, we achieve pulse-number-dependent, multi-level modulation of the skyrmion density. These findings establish a viable pathway towards high-speed, low-power, and multi-level generation of densely packed, ultra-small skyrmions in the vdW magnets, laying the groundwork for skyrmion-based neuromorphic computing architectures.

## Results

### Theoretically predicted densely packed, miniaturized skyrmion in vdW magnets

**Fig. 1:**
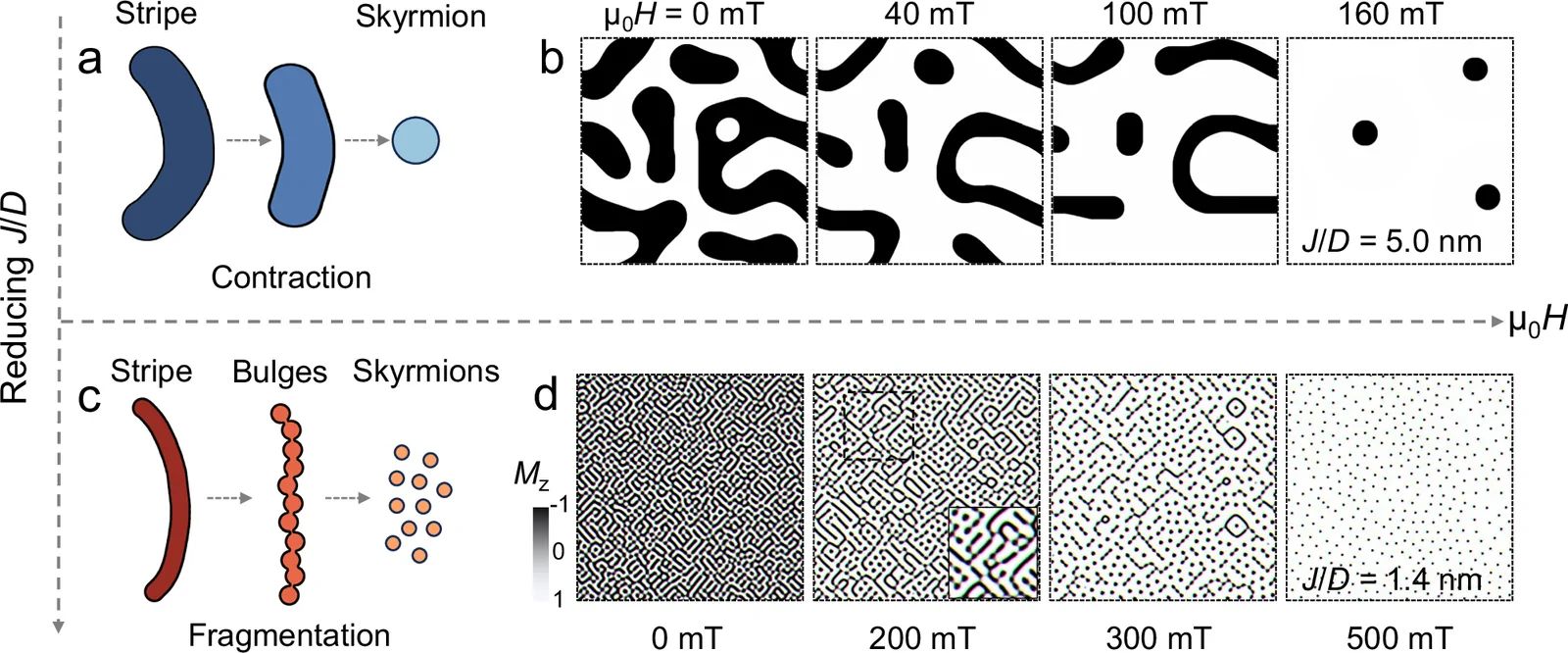
Theoretically predicted densely packed miniaturized skyrmions. **a** Schematic diagram of stripe-skyrmion transition via a contraction mechanism. **b** Simulated magnetization process of magnetic domains using the magnetic parameters experimentally extracted from Fe_3-δ_GaTe_2_. **c** Schematic diagram of stripe-skyrmion transition via a fragment mechanism. **d** Simulated magnetization process of magnetic domains based on a reduced *J*/*D* ratio. The magnetization along the *z*-axis (*M*_z_) is represented by regions in black (-*M*_z_) and white (+*M*_z_). Inset: the amplified view of the image enclosed by the black dashed box.

Room-temperature skyrmions have been recently discovered in several vdW magnets, including (Fe_0.5_Co_0.5_)_5_GeTe_2_^28^, non-stoichiometric Fe_3-δ_GaTe_2_^30–32,34^, Cr_1±δ_Te_2_^36,37^, and Fe_5_GeTe_2_^38^. In these materials, stripe domains form in the absence of external magnetic fields, with their typical width exceeding 100 nm at room temperature^28,30–32,34,36–38^. Upon applying an out-of-plane magnetic field (μ_0_*H*), these stripes progressively contract in length, and each stripe eventually shrinks into, at most, a single skyrmion (schematically illustrated in Fig. 1a). This one-to-one contraction mechanism intrinsically limits the attainable skyrmion density (*ρ*). Moreover, since skyrmions nucleate directly from stripes, their size is inherently tied to the initial stripe width, that is, narrower stripes yield smaller skyrmions, while wider stripes produce larger ones. Consequently, the large intrinsic stripe width in existing vdW magnets leads to skyrmion diameters typically exceeding 100 nm^28,30–32,34,36–38^, imposing a fundamental obstacle to skyrmion miniaturization.

To overcome these constraints and evaluate the feasibility of realizing densely packed, miniaturized skyrmions, we selected non-stoichiometric Fe_3-δ_GaTe_2_ as a model system and initiated our study with micromagnetic simulations. Fe_3-δ_GaTe_2_ was chosen not only because it typifies the magnetization behavior of vdW skyrmion hosts, but also owing to its favorable properties for spintronic applications, such as strong perpendicular magnetic anisotropy^53^, high Curie temperature above room temperature^30–32,53,54^, and pronounced tunnel magnetoresistance effect^55^. We first simulated the room-temperature magnetization behavior of the Fe_3-δ_GaTe_2_ using experimentally derived magnetic parameters (Fig. 1b, Methods, and Suppl. Note 1)^31^, including ferromagnetic exchange constant (*J*), effective magnetic anisotropy constant (*K*_eff_), DMI constant (*D*), and saturation magnetization (*M*_s_). After confirming good agreement between simulations and experiments, we then explored how variations in these parameters influence skyrmion size, density, and evolution dynamics. The simulations reveal that modifications to *J* or *D* remarkably reshape the energy landscape governing the domain evolution process, leading to pronounced effects on skyrmion formation dynamics (Suppl. Fig. S1). In contrast, adjustments to *K*_eff_ and *M*_s_ have relatively minor impact (Suppl. Fig. S2). Among the dominant factors, decreasing *J* or increasing *D* proves particularly effective for stabilizing densely packed, miniaturized skyrmions, aligned with our research objectives. To quantify their interplay, we define *J*/*D* ratio as a key tuning parameter. As *J/D* decreases, the stripe width at zero magnetic field narrows accordingly (Fig. 1c, d and Suppl. Fig. S3), which is a prerequisite for skyrmion miniaturization. Upon increasing μ_0_*H*, the narrowed stripes become less compressible and form numerous bulges along their length. These bulges act as precursors to skyrmions, and beyond a critical field, they evolve into individual skyrmions via a fragmentation mechanism. This process enables a single narrowed stripe to split into multiple skyrmions, thereby simultaneously reducing skyrmion size and increasing packing density. Guided by these simulations, we propose a material-design strategy centered on lowering the *J*/*D* ratio to achieve both skyrmion miniaturization and high density in the vdW magnets.

### Tuning magnetic interactions in the Fe_3-δ_GaTe_2_ via Pd-atom intercalation

**Fig. 2:**
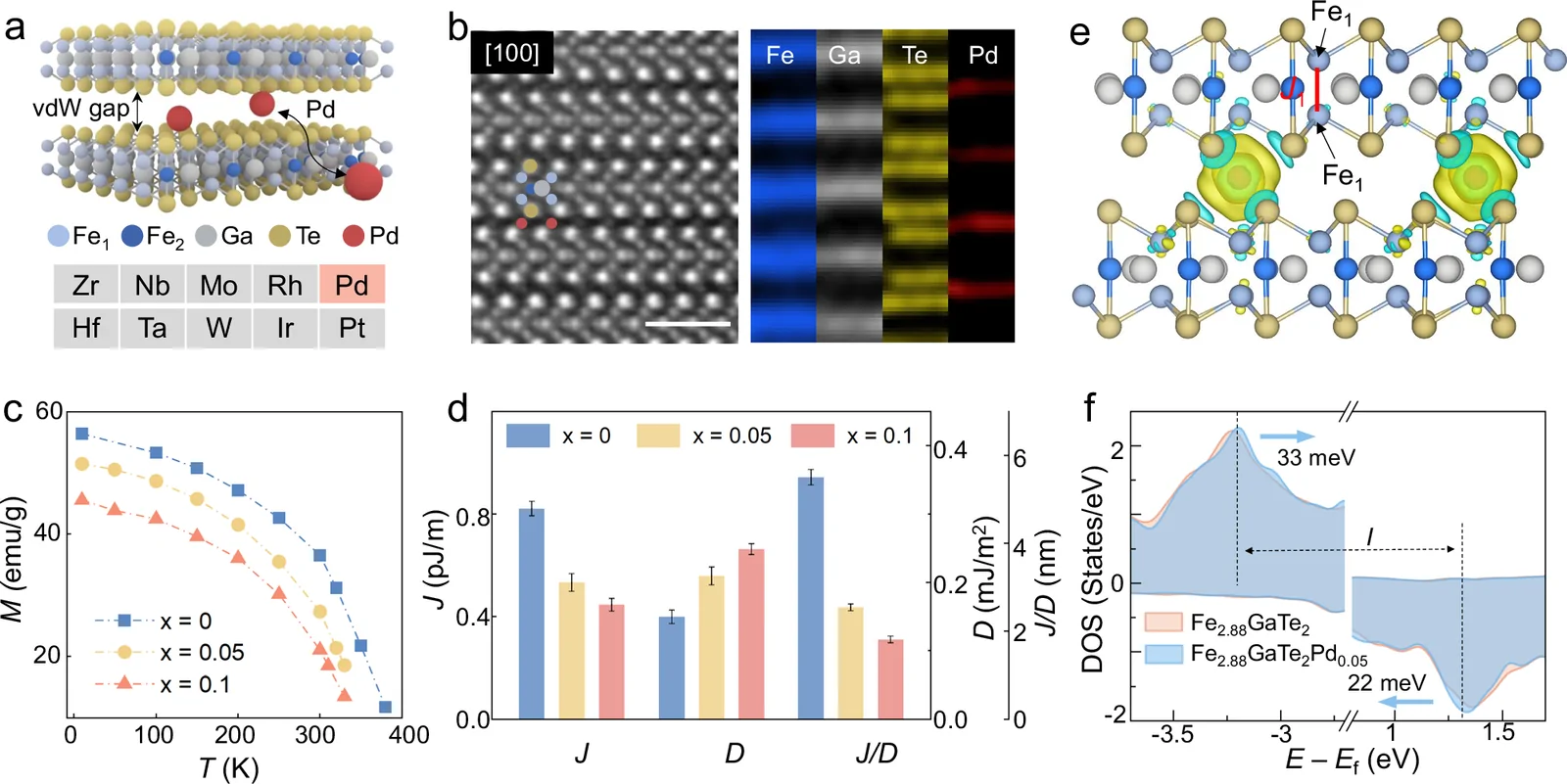
Structure and Magnetic Properties of Pd-Intercalated Fe_3-δ_GaTe_2_. **a** Schematic illustration of Pd atoms intercalated into the vdW gap of Fe_3-δ_GaTe_2_. The table below summarizes the elements used in our intercalation experiments; elements successfully intercalated are marked in pink, while those unsuccessfully intercalated are marked in gray. **b** Left panel: Annular bright-field (ABF) image of Fe_3-δ_GaTe_2_Pd_x_ (x = 0.05) taken along the [100] zone axis. Scale bars represent 1 nm. Right panel: atomic-scale energy-dispersive X-ray spectroscopy (EDX) mapping for Fe (blue), Ga (gray), Te (yellow), Pd (red). **c** Temperature-dependent saturation magnetization (*M*ₛ) for Fe_3-δ_GaTe_2_Pd_x_ (x = 0, 0.05 and 0.1). **d** Exchange constant (*J*), DMI constant (*D*), and the *J*/*D* ratio for Fe_3-δ_GaTe_2_Pd_x_ at room-temperature. The error bar presents the standard deviation. **e** Differential charge density (DCD) map for Pd-intercalated Fe_3-δ_GaTe_2_. Yellow and blue regions indicate charge accumulation and depletion, respectively. Double-sided arrows between Fe_1_ atoms represent the ferromagnetic exchange interaction (*J*_1_). **f** Spin-resolved density of states (DOS) of Fe_1_ atoms for x = 0 (light red) and x = 0.05 (blue). Positive and negative DOS values correspond to spin-up and spin-down states, respectively. Dashed lines mark the DOS peak positions.

To experimentally validate our theoretical predictions, we employ atomic intercalation to engineer the magnetic interactions in the Fe_3-δ_GaTe_2_, as schematically illustrated in Fig. 2a. Recognizing that the DMI scales positively with spin-orbit coupling (SOC), we selected heavy transition metals (HTM) from periods 5 and 6 of the element periodic table (HTM = Zr, Hf, Nb, Ta, Mo, W, Ir, Pt, Rh, Pd) as potential intercalants, aiming to enhance the DMI in the parent compound. The targeted Fe_3-δ_GaTe_2_HTMₓ single crystals were synthesized via a Te-flux method (Methods). Energy-dispersive X-ray spectroscopy (EDX) measurements on their exfoliated flakes revealed that, despite systematic attempts, only Pd was successfully intercalated (Suppl. Fig. S4 and Table S1), reaching a maximum atomic concentration of ~2% for x = 0.2 (x represents Pd’s nominal content). First-principle calculations attribute this selective intercalation to Pd’s lower cohesive energy, migration energy (*E*_m_), and formation energy within the vdW gap (Methods, Suppl. Note 2, Table S2, S3, Figs. S5–S9), which arises from its smaller atomic radius compared to other HTMs. To investigate the structural impact of Pd intercalation, atomic-scale scanning transmission electron microscopy (STEM) performed on the Fe_3-δ_GaTe_2_Pd_x_ (x = 0.05). Annular bright-field (ABF) imaging along the [001] zone axis reveals a six-fold Fe coordination surrounding each Te atom, agreeing with the *ab*-plane atomic arrangement of the pristine Fe_3-δ_GaTe_2_, thereby indicating that Pd intercalation preserves the crystal’s in-plane hexagonal symmetry (Suppl. Fig. S10). To further resolve the structure along the *c*-axis, ABF images were taken along the [100] zone axis. In this view, Te-Fe_3_Ga-Te monolayers are orderly stacked along the *c*-axis (Fig. 2b), consistent with the layered structure of Fe_3-δ_GaTe_2_. Moreover, Fe_2_ atoms (dark blue circles) exhibit an out-of-plane displacement relative to the Ga atoms (gray circles), which breaks the spatial symmetry and induces a pronounced DMI^31^. Importantly, a discernible imaging contrast, significantly weaker than that of the host atoms, is observed within the vdW sites under the Fe_1_ atoms, a feature that is absent in the pristine Fe_3-δ_GaTe_2_^30,31^. Atomic-scale EDX mapping (Fig. 2b), together with electron energy loss spectroscopy (EELS, Suppl. Fig. S11) and X-Ray Diffraction spectrum (XRD, Suppl. Fig. S12), further confirms that these contrast sites are occupied by Pd atoms (red circles). Notably, we observe no evidence of Fe self-intercalation, which is reported in Fe-rich Fe_3+δ_GaTe_2_^56^. This behavior can be attributed to the lower formation energy of Pd atoms occupying the vdW gaps of Fe-deficient Fe_3-δ_GaTe_2_ compared with Fe atoms, as shown in Suppl. Fig. S13.

We then calculated the total energy (*E*_t_) of Fe_3-δ_GaTe_2_Pd_x_ for three atomic configurations, including Pd substituting Fe_1_ (light blue circles), Pd substituting Fe_2_, and Pd intercalated in the vdW gap (Supplementary Fig. S14). The results indicate that Pd-intercalated configuration is energetically most favorable, confirming that Pd preferentially occupies the vdW gap under the equilibrium growth conditions provided by the Te-flux method. However, despite the Pd intercalation, the measured lattice constants (*a* = *b* = 4.05 nm; *c* = 16.15 nm) are nearly identical to those of the Fe_3-δ_GaTe_2_ (*a* = *b* = 4.08 nm; *c* = 16.13 nm)^31^. This minimal change in lattice parameters is further corroborated by structural relaxation calculations (Supplementary Table S4), thus validating our experimental observations.

To illustrate the effect of Pd intercalation on the magnetic interactions in the Fe_3-δ_GaTe_2_, we investigated the variations of *J*, *D*, and *J*/*D* as a function of Pd concentration for x = 0, 0.05, 0.1 at room temperature. First, we extracted *J* by fitting the temperature (*T*)-dependent *M*_s_ curves using the Bloch’s Law^57^ (Fig. 2c and Suppl. Fig. S15). The results reveal a progressive decrease in *J* from 0.82 pJ/m (x = 0) to 0.45 pJ/m (x = 0.1) (Fig. 2d), indicating that Pd intercalation weakens the ferromagnetic exchange coupling. We then established *D* based on the domain wall energy (*σ*) formula^58^:

1 $$\sigma=4\sqrt{J{K}_{{{\rm{eff}}}}}-{{{\rm{\pi }}}}|D|$$

where *K*_eff_ is retrieved by calculating the area difference between the in-plane and out-of-plane magnetization curves (Suppl. Fig. S16); *σ* is calculated using the domain spacing model^58^:

2 $$\,\frac{\sigma }{{{\mu }}_{0}{{{\rm{M}}}}_{{{\rm{s}}}}^{2}{{\rm{h}}}}=\frac{{{{\rm{w}}}}^{2}}{{{{\rm{h}}}}^{2}}{\sum }_{{{{\rm{odd}}}}\,{n}=1}^{\infty }\left(\frac{1}{{({{{\rm{\pi }}}}{{\rm{n}}})}^{3}}\right)\left[1-(1-2{{{\rm{\pi }}}}{{\rm{nh}}}/{{\rm{w}}})\exp \left(-2{{{\rm{\pi }}}}{{\rm{nh}}}/{{\rm{w}}}\right)\right],$$

where *h* is the sample thickness and *w* is the low-field domain period (detailed procedures for establishing *σ* are provided in Suppl. Fig. S17). By combining Eqs. (1) and (2), D for Fe_3-δ_GaTe_2_Pd_x_ with x = 0, 0.05, 0.1 were calculated (Fig. 2d). We observed a significant enhancement in *D* from ~0.15 mJ/m² for x = 0 to ~0.25 mJ/m² for x = 0.1. More importantly, the corresponding *J*/*D* ratio drops sharply from 5.5 nm to 1.8 nm (Fig. 2d), creating favorable conditions for stabilizing high-density, miniaturized skyrmions.

We further clarified the physical origin of the weakened ferromagnetic exchange coupling and enhanced DMI. In Fe_3-δ_GaTe_2_, the exchange coupling is predominantly governed by the coupling between Fe_1_ atoms along *c*-axis (*J*_1_), as schematically shown in Fig. 2e (calculations details are presented in Supplementary Note 2, Fig. S5 and Table S5). Since Pd intercalation induces negligible lattice changes, the observed reduction in exchange strength is unlikely due to variations in the Fe_1_-Fe_1_ bond length. Instead, it is attributed to the charge redistribution of Fe_1_ atoms triggered by Pd intercalation. Differential charge density (DCD) calculations for Pd-intercalated Fe_3-δ_GaTe_2_ (Fig. 2e and Suppl. Note 2) show that Pd atoms, due to their higher electronegativity (2.2 for Pd vs. 2.0 for Te), gain electrons (yellow regions) from neighboring Te atoms, resulting in a charge redistribution throughout the material system. This redistribution significantly affects the ferromagnetic exchange coupling between Fe₁ atoms, as described by the Stoner model^59^, where the coupling strength scales with the band exchange splitting (*I*)^59^, defined as the energy difference between the spin-up and spin-down density of states (DOS) peaks, as indicated by the dashed line in Fig. 2f. The calculations reveal that the Pd-induced charge transfer shifts the spin-up channel of the Fe_1_-3d bands (positive DOS) to a higher energy level by ~33 meV and the spin-down channel (negative DOS) to a lower energy level by ~22 meV (Fig. 2f). This process leads to a net reduction in exchange splitting of ~55 meV, thus accounting for the observed weakening of ferromagnetic exchange coupling. Furthermore, our calculations confirm that Pd intercalation increases the DMI (Suppl. Fig. S18 and Tables S6, S7), agreeing with the experimental observations. This enhancement arises from two main factors: (i) the intercalation of Pd atoms with relatively strong SOC amplifies the overall SOC of the system; and (ii) Pd intercalation modifies the local atomic arrangement, which increases structural inversion asymmetry and opens additional chiral exchange pathways, thus further strengthening the DMI.

### Real-space observation of densely packed, sub-50 nm skyrmions using LTEM

**Fig. 3:**
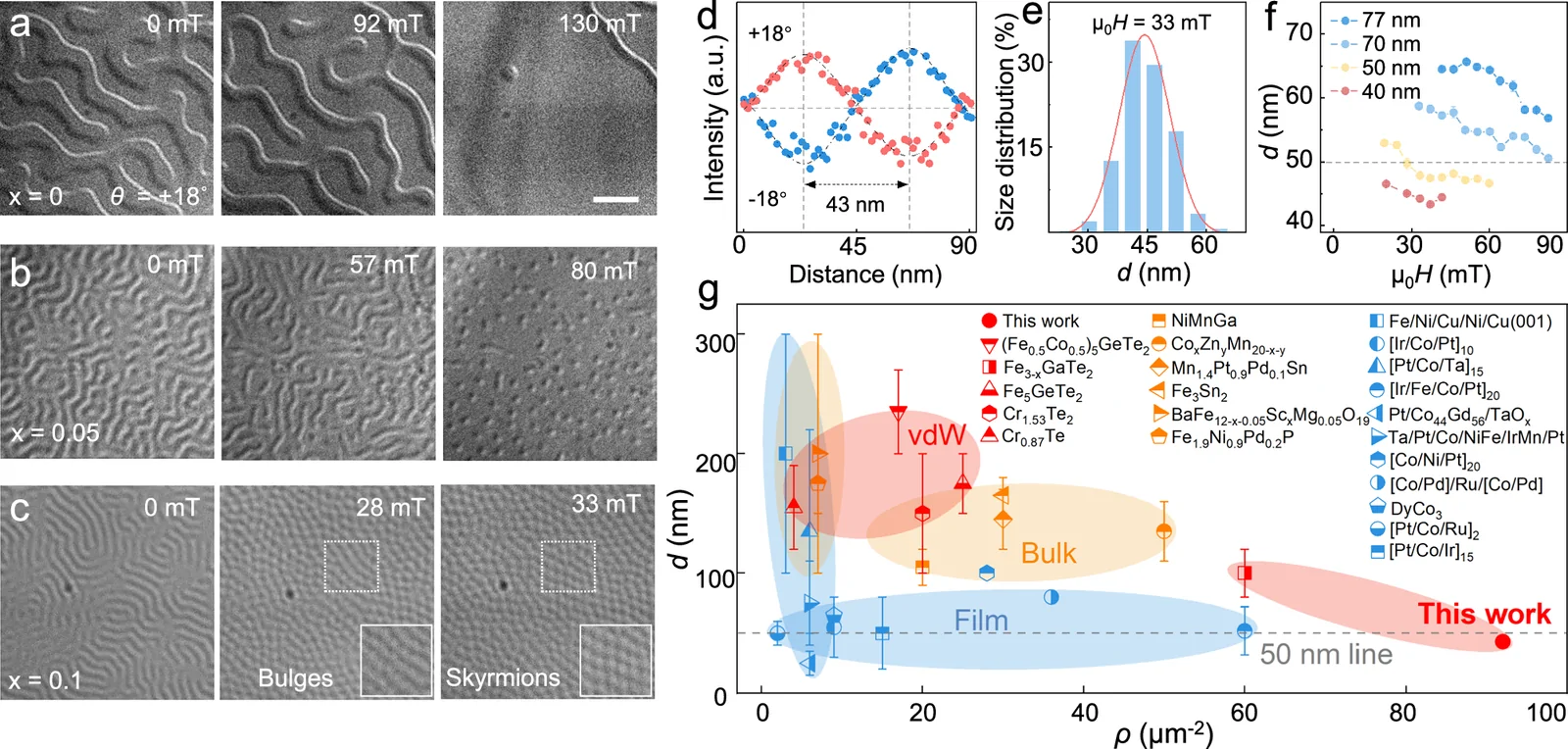
LTEM images of densely packed miniaturized skyrmions. Evolution of magnetic domains with increasing magnetic field in Fe_3-δ_GaTe_2_Pdₓ with (**a**) x = 0, (**b**) 0.05, (**c**) 0.1. The scale bar is 500 nm. The images were acquired with the sample titled at *θ* of 18° relative to the beam axis. Insets show magnified views of the regions enclosed by white dashed boxes. **d** Line profile of a typical skyrmion image extracted from the right panel of (**c**) at *θ* = ±18°, yielding a diameter of 43 nm. **e** Size distribution of skyrmions for x = 0.1 sample at μ_0_*H* = 33 mT. **f** Thickness dependence of skyrmion diameter for x = 0.1 sample. The error bar presents the standard deviation. g, Comparison of skyrmion densities and diameters at room temperature for different ferromagnetic materials, including vdW ferromagnetic metals (red markers), including (Fe_0.5_Co_0.5_)GeTe_2_^28^, Fe_3-x_GaTe_2_^31^, Cr_1.53_Te_2_^36^, Cr_0.87_Te^37^, Fe_5_GeTe_2_^38^, bulk ferromagnetic alloys (orange markers), including Fe_3_Sn_2_^46^, Co_x_Zn_y_Mn_20-x-y_^65^, Mn_1.4_Pt_0.9_Pd_0.1_Sn^66^, Fe_1.9_Ni_0.9_Pd_0.2_P^67^, NiMnGa^68^, BaFe_12-x-0.05_Sc_x_Mg_0.05_O_19_^69^, and ferromagnetic multilayers (blue markers), including [Ir/Fe/Co/Pt]_20_^64^, [Ir/Co/Pt]_10_^70^, [Pt/Co/Ta]_15_^71^, Pt/Co_44_Gd_56_/TaO_x_^72^, [Pt/Co/Ru]_2_^73^, [Pt/Co/Ir]_15_^74^, Fe/Ni/Cu/Ni/Cu^75^, Ta/Pt/Co/NiFe/IrMn/Pt^76^, [Co/Ni/Pt]_20_^77^, [Co/Pd]/Ru/[Co/Pd]^78^, DyCo_3_^79^. The error bar presents the size range of skyrmions.

To directly image the optimized skyrmion characteristics, we conducted Lorentz transmission electron microscopy (LTEM) observations on the Fe_3-δ_GaTe_2_Pd_x_ (x = 0, 0.05, 0.1) flakes under varying out-of-plane magnetic fields at 300 K (Fig. 3a–c). These flakes, prepared via mechanical exfoliation, exhibit thickness (*t*) of ~40 nm (Suppl. Fig. S19). Initially, with the electron beam perpendicular to the sample plane, no discernible magnetic contrast is observed. However, tilting the sample by an angle (*θ*) of 18° relative to the beam axis induces the emergence of stripes (Fig. 3a–c). This angular dependence feature suggests that the observed magnetic domains are Néel-type, consistent with the DMI-stabilized spin textures reported in previous vdW magnets^28,30–35,60^.

We then examined the magnetic field-dependent skyrmion size, density, and evolution process of the Fe_3-δ_GaTe_2_Pd_x_. For x = 0, skyrmion formation follows the one-to-one stripe contraction mechanism, yielding only few isolated skyrmions with relatively large diameter of ~134 nm (Fig. 3a). As Pd concentration increases, corresponding to a reduced *J*/*D* ratio, the stripe width at μ_0_*H* = 0 mT decreases significantly from ~180 nm for x = 0 to ~52 nm for x = 0.1. Since skyrmions nucleate from stripes, narrowing their width is crucial for achieving smaller skyrmion sizes. Beyond size reduction, Pd intercalation also modifies the skyrmion formation mechanism. At low Pd concentrations, the transition is primarily governed by stripe contraction (Fig. 3a, b). As the Pd content increases, in contrast, the mechanism shifts toward stripe fragmentation (Fig. 3c and insets), where increasing μ_0_*H* induces the stripes to form bulges along their length, which subsequently fragment into individual skyrmions. This transition from contraction to fragmentation mechanism corresponds to the reduction of *J*/*D* ratio, in line with our theoretical predictions (Fig. 1). Crucially, the fragmentation mechanism enables a single stripe to break into multiple skyrmions, thereby driving a substantial enhancement in skyrmion density, reaching a peak of ~92 μm^−^^2^ for x = 0.1 at μ_0_*H* = 33 mT.

The diameter of a Néel-type skyrmion is typically defined as the distance between two in-plane spins oriented 180° apart^27,29,61,62^. In LTEM images, this corresponds to the distance between the darkest and brightest points, as schematically illustrated in Suppl. Fig. S20. Since LTEM imaging is conducted under defocused conditions, the measured skyrmion diameter may appear either underestimated (under-focus) or overestimated (over-focus) relative to the actual size. To correct this distortion, we first acquire an in-focus LTEM image of a marker for scale calibration. The corresponding under-focused image is then rescaled by applying a 1.2× correction factor to the marker size, ensuring accurate diameter measurements (see Suppl. Fig. S21 for detailed procedures to establish the correction factor). After this distortion correction, the skyrmion diameter (*d*) is extracted from the peak-to-valley distance of the line profile (Fig. 3d). To evaluate the dependence of *d* on out-of-plane magnetic field, we measured nearly all the skyrmions in each LTEM image and calculated their average values under varying fields (Fig. 3e). For x = 0.1, at the critical magnetic field for skyrmion formation (μ_0_*H* = 28 mT), the average skyrmion diameter is ~45 nm. As μ_0_*H* increases, *d* decreases slightly, reaching a minimum of ~43 nm at μ_0_*H* = 33 mT. These results are highly reproducible, as confirmed by similar measurements in three different x = 0.1 flakes (Suppl. Fig. S22). Thickness-dependent measurements show that when the sample is thinned to ~30 nm, the magnetic contrast vanishes completely (Suppl. Figs. S23–25). The disappearance of magnetic contrast is attributed to the thickness-induced reduction of *T*_C_ (Suppl. Fig. S26)^53,63^. In contrast, increasing the thickness enlarges the skyrmion diameter, which exceeds ~55 nm at ~77 nm (Fig. 3f and Suppl. Figs. S23–25). This size increase is attributed to the strengthened stray field, which broadens the skyrmion’s spin-swirl region. Compared with previously reported skyrmion diameters in other vdW magnets at room temperature^28,30–32,34,36–38^, the skyrmions in Pd-intercalated Fe_3-δ_GaTe₂ exhibit much smaller diameters (~43 nm), nearly two times smaller than those values (Fig. 3g). While sub-50 nm skyrmions with relatively high density (~60 μm^−^^2^) have been achieved in [Ir/Fe/Pt/Co/]_20_ multilayers^64^, our system reaches a density of 92 μm^−2^, exceeding the previous record by more than 1.5 times and representing the highest value across all room-temperature skyrmion-hosting materials (Fig. 3g)^28,30–32,34,36–38,46,64–79^. This simultaneous optimization of both skyrmion size and density within a single vdW represents a significant advancement towards skyrmion-based spintronic device applications.

### Ultrafast laser-induced multi-level generation of sub-50 nm skyrmions

High-speed, energy-efficient generation of densely packed nanoscale skyrmions is crucial for advancing high-performance spintronic devices. Fs laser excitation, leveraging ultrafast optical-thermal effect, offers a promising pathway. Generally, upon fs laser excitation, the system undergoes sequential phase transitions: the electron temperature (*T*_elec_) rapidly exceeds surpassing the Curie temperature (*T*_C_), leading to the melting of magnetic order within the fs regime; this is followed by a picosecond-scale thermal quenching of the paramagnetic state mediated by electron-phonon coupling^13–21^. Such dynamics enables the generation of metastable skyrmions on picosecond timescales^13–21^, providing a viable strategy for high-speed skyrmion nucleation.

**Fig. 4:**
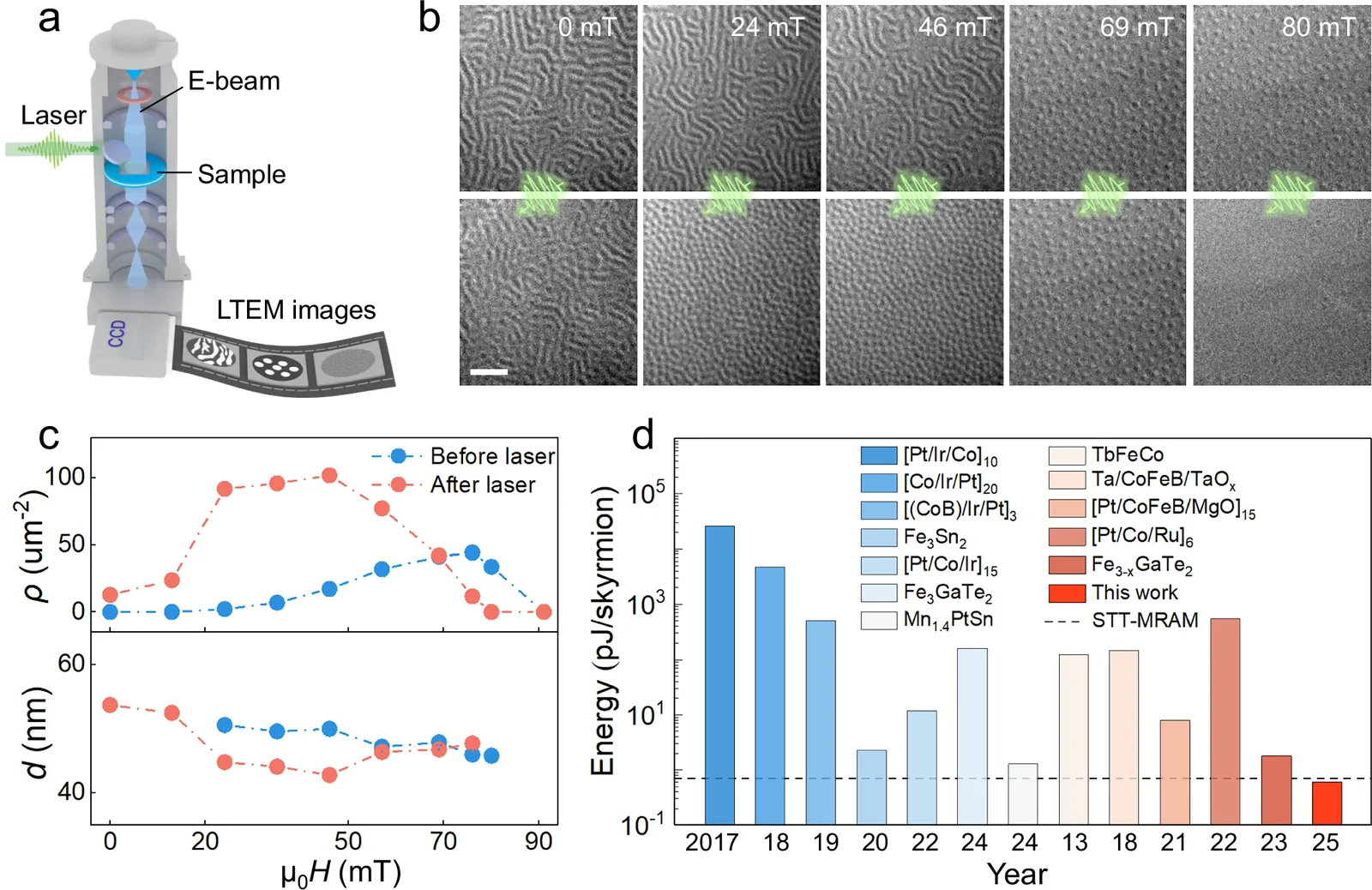
Skyrmion Generation Induced by Femtosecond Laser Excitation. **a** Schematic illustration of the custom-built in situ LTEM setup enabling single-shot fs laser pulse excitation. The sample is irradiated with a green femtosecond laser pulse (wavelength: 520 nm, pulse duration: 240 fs) under an out-of-plane magnetic field (μ_0_*H*). **b** LTEM images of Fe_3-δ_GaTe_2_Pd_x_ (x = 0.05) taken before (upper panel) and after (lower panel) femtosecond laser excitation under varying out-of-plane magnetic fields. The scale bar is 500 nm. **c** Statistical analysis of skyrmion density (*ρ*) and diameter (*d*) as a function of magnetic field (μ_0_*H*) before (blue circles) and after (red circles) fs laser irradiation. **d** Comparison of energy consumption between current-driven (blue markers), including Fe_3_Sn_2_^46^, Fe_3_GaTe_2_^60^, [Pt/Co/Ir]_15_^74^, Mn_1.4_PtSn^82^, [Pt/Ir/Co]_10_^83^, [Co/Ir/Pt]_20_^84^, [(CoB)/Ir/Pt]_3_^85^, and femtosecond laser-induced (red markers) skyrmion generation, including TbFeCo^13^, Ta/CoFeB/TaO_x_^15^, [Pt/CoFeB/MgO]_15_^17^, [Pt/Co/Ru]_6_^18^, Fe_3-x_GaTe_2_^31^. The dashed line indicates the energy threshold for industry STT-MRAM devices^80,81^.

To evaluate the feasibility of ultrafast laser-induced generation of sub-50 nm skyrmions in the Pd-intercalated Fe_3-δ_GaTe_2_, we performed LTEM measurements under fs laser excitation using a home-built in situ LTEM setup, as schematically illustrated in Fig. 4a. This system enables single-shot fs laser pulse excitation on the micrometer scale (520 nm wavelength, ~50 μm focal spot size, and ~240 fs pulse duration). During laser exposure, the electron beam was blanked to avoid unintended sample damage. Figure 4b presents LTEM images of the x = 0.05 sample acquired before and after laser exposure at a fluence of ~6.0 mJ/cm² under varying out-of-plane magnetic fields. All these images were taken from the same sample area, with a thermal demagnetization step applied before varying the magnetic field to remove remanent magnetization. These results reveal that the external magnetic field is crucial for fs laser-induced skyrmion generation. At zero magnetic field (μ_0_*H* = 0 mT), the sample maintains stripes with no skyrmion formation after the laser excitation. When μ_0_*H* increases to 13 mT, laser excitation induces a mixed phase containing both stripes and skyrmions (Suppl. Fig. S27). Further increasing μ_0_*H* to a critical threshold of 24 mT eliminates the stripes, leaving a densely packed skyrmion phase. In contrast to the conventional magnetic field method (μ_0_*H* = 69 mT, upper panel of Fig. 4b), the required stabilization field for laser-assisted skyrmion writing is dramatically reduced, indicating that the laser pulse effectively provides an energy that is equivalent to an effective field, which enables the transition from lower-energy stripe states to higher-energy skyrmion states. However, this trend reverses at elevated magnetic fields above 69 mT (Fig. 4b and Suppl. Fig. S27). In this regime, laser excitation reduces skyrmion density, eventually achieving complete out-of-plane magnetization saturation at 80 mT. In contrast to the single domain state stabilized purely by a high magnetic field of μ_0_*H* = 90 mT (Suppl. Fig. S27), the substantial reduction in required field further confirms the laser’s effectiveness in reshaping the magnetic energy landscape for spin texture control.

Following fs laser excitation, a minimum skyrmion diameter of ~43 nm together with a maximum density of ~101 μm^−2^ is achieved (Fig. 4c). Both values are remarkably optimized, compared to those achieved in the x = 0.05 sample under magnetic field alone. This pronounced enhancement underscores the effectiveness of fs laser excitation in tuning skyrmion properties. The underlying mechanism can be attributed to the “freezing” effect of the ultrafast laser. In ferromagnetic systems exhibiting DMI, *J* generally decreases with increasing temperature due to thermal fluctuations, and eventually approaches zero near the Curie temperature (Suppl. Fig. S15). In contrast, *D* remains relatively insensitive to temperature changes, and thus, the *J*/*D* ratio decreases as the temperature rises. During laser-induced field cooling, the system undergoes rapid thermal excitation followed by ultrafast cooling at rates exceeding 10^6^–10^8^ K/s^14^. This process kinetically freezes the high-temperature skyrmion phase into a metastable state at room temperature. Owing to a lower *J*/*D* ratio associated with the high-temperature phase, the resulting room-temperature skyrmions exhibit smaller diameters and higher densities for x = 0.05 sample (Fig. 3b). Notably, the average energy dissipation for generating a single skyrmion is only 0.6 pJ, comparable to that of the spin-transfer torque magnetoresistive random-access memory (STT-MRAM), which typically ranges from 0.7 to 1.4 pJ/bit^80,81^, and over an order of magnitude lower than that required for the current-induced skyrmion manipulation and motion^46,60,74,82–85^ (Fig. 4d). Additionally, the energy dissipation is approximately one third of that required in pristine Fe_3-δ_GaTe_2_. This significant reduction arises from two combined effects introduced by Pd intercalation: (i) a lower Curie temperature and (ii) a significantly higher skyrmion density. A detailed explanation is provided in Supplementary Note 3. By achieving such low-energy operation, our approach enables a technically energy-efficient means for high-speed skyrmion generation.

**Fig. 5:**
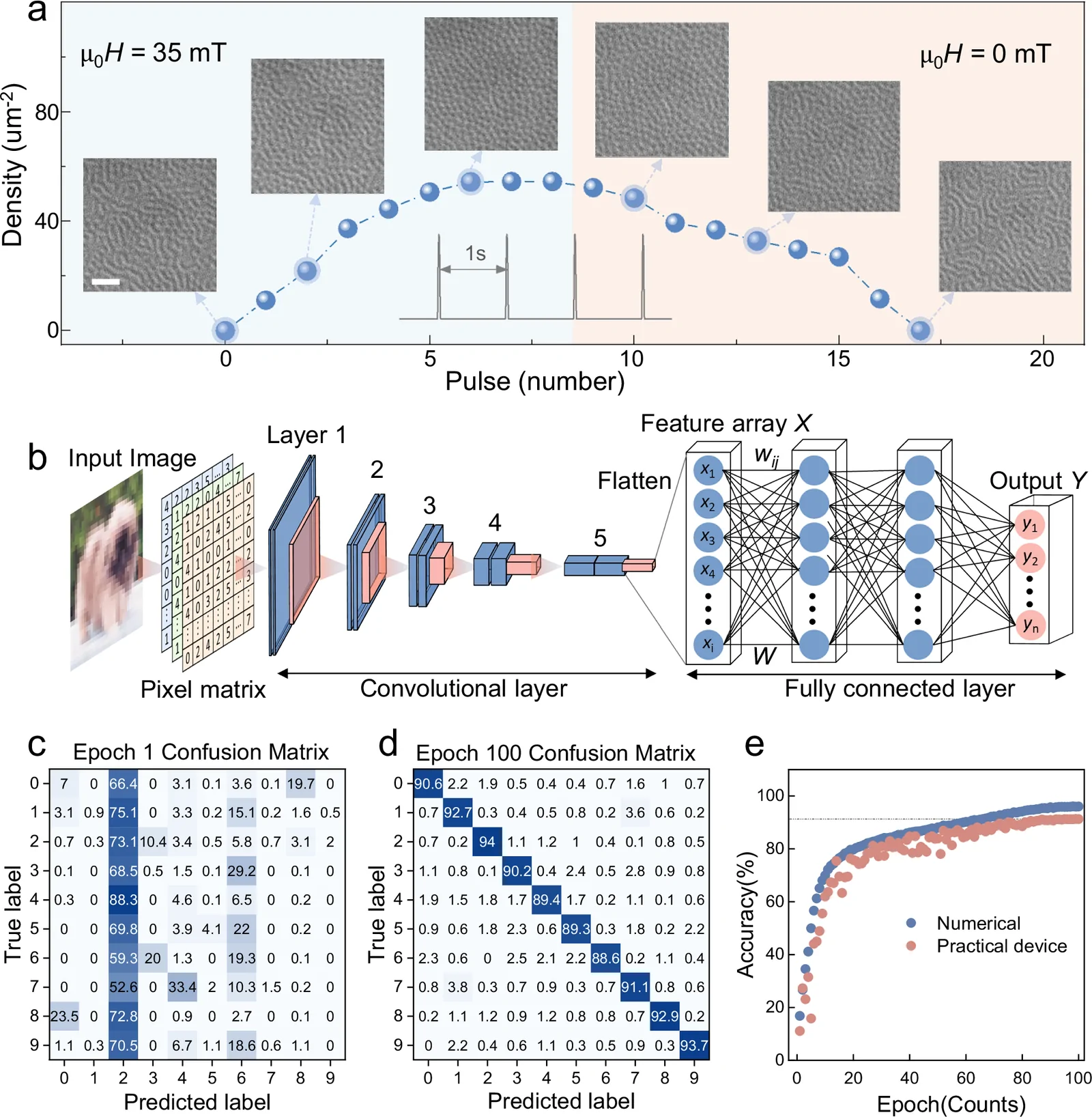
Laser-Induced Multi-Level Manipulation of Skyrmion Density Towards Neuromorphic Computing. **a** Dependence of skyrmion density (*ρ*) on laser pulse number (*N*) at μ_0_*H* = 35 mT (blue region) and μ_0_*H* = 0 mT (pink region), with a laser influence of 4.9 mJ/cm^2^ and a duration of 1 s. Scale bar is 500 nm. **b** Network structure of VGG network-16 for CIFAR−10 image classification. The network is composed of convolutional layers (layers 1–5) and fully connected layers (cuboid with blue and red circles). Each convolutional block comprises two convolution operations (blue cuboid) with a kernel size of 3 × 3 (stride = 1, padding = 1), followed by a max-pooling operation (red cuboid) with a 2 × 2 kernel (stride = 2). The convolutional layers are responsible for feature extraction, while the pooling layers reduce spatial dimensionality and mitigate overfitting. The input is a 32 × 32-pixel RGB image. Feature maps are progressively transformed as follows: 64 feature maps with a size 64@16 × 16 (layer 1), 128@8 × 8 (layer 2), 256@ 4 × 4 (layer 3), 512@2 × 2 (layer 4), and 512@1 × 1 (layer 5). Finally, the features are flattened to 512 one-dimensional vector and fed into the full connection layer. The black lines denote weight factors (*w*_ij_) in the weight matrix *W*, reflecting the transformation between successive fully connected layers. **c** The confusion matrix of predicted and actual values for image recognition task before training for 100 epochs. **d** The confusion matrix of predicted and actual values for image recognition task after training for 100 epochs. **e** Comparison of recognition accuracy between the device and the ideal network.

We further demonstrate that fs laser excitation enables multi-level control over skyrmion density through a pulse-number-dependent process. Figure 5a shows a series of LTEM images captured after laser exposure at a reduced fluence of ~4.9 mJ/cm^2^. Initially, the sample exhibits only stripes, with no visible skyrmions. Upon application of the first laser pulse, isolated skyrmions begin to nucleate from the stripe background. As the number of pulses increases, the skyrmion density rises monotonically and reaches saturation after 7 pulses, indicating a clear non-linear accumulative effect of optical excitation (light blue background in Fig. 5a; Suppl. Fig. S28 for more details). Interestingly, once the skyrmions are formed, reducing the external magnetic field to zero does not trigger a reversion to the stripe domains. Instead, they persist and expand in size, forming a stable, field-free skyrmion configuration (Fig. 5a and Supplementary Fig. S28). The robustness of the skyrmions can be attributed to their topological protection and the sample’s remanent magnetization, which together provide the internal energy landscape necessary to sustain skyrmions even in the absence of an external magnetic field. However, when laser pulses (the fluence is ~4.9 mJ/cm^2^) were further applied at μ_0_*H* = 0 mT, a reverse multi-level transformation was observed (pink background in Fig. 5a). Specifically, the skyrmions gradually merged back into stripes as the pulse number increased, and the skyrmion density decreased continuously until it vanished after 9 pulses. This pulse-number-controlled, multi-level transformation between skyrmions and stripes can be reproducible across different samples and circled in the same sample (Suppl. Fig. S29), demonstrating the reliability of optically driven multi-level skyrmion modulation.

Building on this functionality, we demonstrate that such multi-level optical control over skyrmion density is well-suited for implementing neuromorphic computing based on the synapse-neuron architectures. Circuit-level performance is examined using a simulated artificial neural network chip based on the Visual Geometry Group (VGG) architecture. As shown in Fig. 5b, the input image is converted into a numerical matrix based on its pixel arrangement. Key features of the matrix are then extracted through convolutional layers, and are transformed into a feature array *X*, where each element *x*_i_ represents a specific feature of the image. A detailed description of the convolutional layers is presented in Suppl. Note 4 and Suppl. Fig. S30. For image classification, the feature array *X* is weighted and summed to produce an output probability *y*_n_ for each image category. The category with the highest probability is selected as the classification result. In this process, each weight factor *w*_*ij*_ (represented by black lines in Fig. 5b) in the weight matrix *W* denotes the relationship between image features and categories, and is a multivalued parameter, as illustrated in Suppl. Note 4 and Suppl. Fig. S31. The parameter of neural network needs to be implemented by multistate physical states of the device in practical applications. In this work, the number of skyrmions encodes synaptic weight, and the pulse-number-dependent evolution mimics the gradual potentiation (increasing the number of skyrmions) and depression (decreasing the number of skyrmions), analogous to biological learning behavior^46,49^. Specifically, the pulse-dependent number of skyrmions are first normalized to match the weight range of the neural network and then used to replace the weight parameters of the convolutional layers and fully connected layers. In practical applications, laser-driven skyrmion devices are proposed to be assembled into arrays, where each weight factor can be mapped onto the skyrmion number, thereby completing the hardware implementation of the neural network.

The image classification and recognition tasks are trained on Canadian Institute for Advanced Research-10 (CIFAR-10) dataset, which includes 10,000 images and consists of 10 image categories labeled from “0” to “9”. A confusion matrix is used to evaluate the classification performance of the network in both the convolutional and fully connected layers using the proposed skyrmion-based device, where the diagonal values indicate the proportion of correctly classified samples, and the off-diagonal values represent misclassifications. Figure 5c shows the confusion matrix after a single training epoch, reflecting the network’s initial untrained state with substantial classification errors across all categories. In contrast, after 100 epochs, the matrix evolves into a sharp diagonal pattern (Fig. 5d), indicating effective learning and accurate class discrimination, with most predictions correctly matching the true labels. The system achieves a training accuracy of ~91% (Fig. 5e), approaching the 96% benchmark of an ideal network. The accuracy discretization can be attributed to the fact that the experimentally obtained number of skyrmion states is limited, which makes certain neighboring weight values mapped onto the same experimentally measured state. These results demonstrate that optical skyrmion-based neuromorphic architectures can deliver performance levels comparable to state-of-the-art CMOS-based approaches while offering the inherent ultrafast, energy-efficient advantages.

## Discussion

In summary, we achieve ultrafast multi-level control of sub-50 nm skyrmions at room temperature using a dual-control strategy that combines Pd intercalation into the vdW gap of Fe_3-δ_GaTe_2_ with ultrafast laser excitation. Through Pd atomic intercalation, we enhance the DMI and suppress Heisenberg exchange coupling in the vdW magnet Fe_3-δ_GaTe_2_, enabling magnetic field-stabilized skyrmions with an average diameter of ~43 nm at room temperature, the smallest skyrmions reported in vdW magnets to date. On this tailored platform, we further demonstrate the fs laser-induced ultrafast generation of ~43 nm skyrmions with a record-high density of ~101 μm^−^^2^ and an ultra-low energy consumption of 0.6 pJ per skyrmion. Crucially, by tuning the laser pulse number, we achieve pulse-number-dependent, multi-level modulation of the skyrmion density. These results lay the foundation for the development of high-speed, low-power skyrmion-based neuromorphic computing.

## Methods

### Micromagnetic simulation

Micromagnetic simulations were performed using the GPU-accelerated simulation package MuMax^3^. The simulated geometry was a slab with dimensions of 512 × 512 × 64 nm^3^ and a mesh size of 2.0 × 2.0 × 2.0 nm^3^. Periodic boundary conditions were applied to emulate large-scale simulations. The total energy of the system includes contributions from exchange interaction, first-order uniaxial magnetic anisotropy, Dzyaloshinskii-Moriya interaction (DMI), demagnetization energy, and Zeeman energy. The initial magnetization process is simulated using magnetic parameters guided by experimental measurements^31^, including ferromagnetic exchange constant (*J*), effective magnetic anisotropy constant (*K*_eff_), DMI constant (*D*), and saturation magnetization (*M*_s_). The specific values used are: *J* = 1.1 pJ/m; *K*_eff_ = 1.5 × 10^5^ J/m^3^; *D* = 0.25 mJ/m^2^; *M*_s_ = 2.5 × 10^5^ A/m. Gilbert damping (*α*) is set to be 0.1, following the experimentally reported value of 0.075 for Fe_3_GaTe_2_ at 300 K^86^.

### Single crystal growth and structure characterization

Single crystals of Fe_3-δ_GaTe_2_HTM_x_ (HTM = Zr, Hf, Nb, Ta, Mo, W, Ir, Pt, Rh, Pd) were grown using a Te-flux method. Fe (99.99%), HTM (99.99%), Ga (99.99%), and Te (99.99%) elements were mixed in molar ratio 1: x: 1: 2 (x for Pd is 0, 0.05, 0.1, 0.2; x for other HTMs is 0.1). To prevent oxidation, all weighing and mixing processes were carried out in an argon-filled glovebox. The mixture was then loaded into an alumina crucible, which was subsequently enclosed in a quartz tube and sealed under high vacuum. Prior to sealing, the quartz tube was purged twice under low vacuum (< 10 Pa) and once under high vacuum (10^−^^4^ Pa). The sealed ampoule was placed in a muffle furnace, with its position close to the furnace thermocouple to ensure accurate temperature control. The ampoule was heated to 1273 K over 10 h and held for 24 h. During the holding stage, the ampoule was gently shaken to promote melt homogeneity. The temperature was then reduced to 1153 K within 1 h, followed by slow cooling to 1033 K over 120 h, and subsequently maintained at this temperature for an additional 167 h. Finally, the excess molten flux was removed by centrifugation to obtain Fe_3-δ_GaTe_2_Pd_x_ single crystals, with typical lateral size on the order of 0.5–1 mm. During centrifugation, the sample undergoes natural cooling, enabling the formation of a thermodynamically stable phase. According to the Ga-Te phase diagram, the centrifugation temperature of 1033 K is slightly below the formation temperature of Ga-Te binary phases (*T*_Ga-Te_ ~ 1049 K)^87^. Therefore, before centrifugation, Ga-Te secondary phases have already formed together with the target Fe_3-δ_GaTe_2_Pd_x_ crystals in the crucible. After centrifugation, these solid phases were retained in the crucible, while only the liquid flux was removed. To evaluate the extent of these secondary phases, we performed energy-dispersive X-ray spectroscopy (EDX, Quattro S) measurements on 23 randomly selected crystals collected from the retained solid products. The results indicate that approximately 70% of the crystals correspond to the target Fe_3-δ_GaTe_2_Pd_x_, while the remaining ~30% are Ga-Te secondary phases. Therefore, although secondary phases are present, Fe_3-δ_GaTe_2_Pd_x_ remains the dominant crystalline product under our growth conditions.

### First-principle calculations

Based on density functional theory (DFT), we employed the Vienna ab initio simulation package (VASP) to calculate the forces, electronic structures, and magnetic properties of the non-stoichiometric Fe_3-δ_GaTe_2_ and Pd-intercalated Fe_3-δ_GaTe_2_ (that is, Fe_3-δ_GaTe_2_Pd_x_). The exchange-correlation interaction was described by the Perdew-Burke-Ernzerhof (PBE) functional under the generalized gradient approximation (GGA). The strong correlation between the localized *d* orbitals was taken into account by including a Hubbard U term through the GGA + U formulation^88^. An effective U value of 2.5 eV was applied. The interaction between valence electrons and ionic cores was described within the framework of the projector augmented wave (PAW) method. The energy cutoff of the plane-wave basis was set to 500 eV, and the Brillouin Zone was sampled by Γ-centered k-point mesh of 0.02 Å^−^^1^. During geometry optimization, both lattice constants and atomic positions were fully relaxed until the force on each atom was less than 0.01 eV/Å and the energy convergence criterion was less than 10^−6 ^eV. Moreover, vdW interactions were included using the DFT-D3 in all calculations.

### LTEM measurements

The samples for LTEM imaging were prepared using an all-dry mechanical transfer method inside an argon-filled glovebox. Their thickness was measured using atomic force microscopy (MFP-3D, Asylum Research). Magnetic domain imaging was conducted using a Thermo Fisher Talos F200i Transmission Electron Microscope (TEM) operated at an acceleration voltage of 200 kV. A magnetic field was applied along the electron beam direction by adjusting the excitation current of the objective lens. Three types of images, namely under-focus, over-focus, and in-focus, were captured using a charge-coupled device (CCD) camera. The scale bars of the under-focus and over-focus images were calibrated based on the in-focus images. In-situ ultrafast laser LTEM measurements were performed in a Thermo Fisher Talos F200i TEM integrated with a near-infrared (1030 nm) fs laser system (YF-FL-50-200-IR, YactoFiber) coupled with a second harmonic generation. The laser pulses were controlled by a digital-delay-generator-controlled shutter and delivered as single shots with a central wavelength of 520 nm and a pulse duration of ~240 fs. The laser spot size was focused to ~50 μm to ensure uniform excitation across the sample area.

## Supplementary information

Supplementary Information

Transparent Peer Review file

## Data Availability

The data supporting the findings of this study are available in the paper and its Supplementary Information.
