# Supplementary material for: Ultrafast multi-level control of sub-50 nm skyrmions in a Pd-intercalated van der Waals magnet
Source: Nat Commun. 2026 Jul 21;17:8907. doi: 10.1038/s41467-026-75870-4 (PMC13503746; doi:10.1038/s41467-026-75870-4)
Supplement: Supplementary file 1 [file 41467_2026_75870_MOESM1_ESM.pdf]

Supplementary information for

Ultrafast Multi-level Control of Sub-50 nm Skyrmions in a Pd-Intercalated van der Waals Magnet

Authors: Huai Zhang[1#], Bei Ding[1#], Bo Zhao[2,3#], Ke Pei[4#], Yue Hu[5], Runhang Zhang[6], Rui Su[7], Minghao Zheng[1], Zefang Li[5], Qing Zhou[8], Guoping Zhao[9], Xingsen Gao[1], Yangfan Hu[6], Xue Jiang[2*], Jijun Zhao[2], Renchao Che[4*], Xuewen Fu[5,10*], Zhipeng Hou[1*], Junming Liu[1,11]

Affiliations:

[1]Guangdong Provincial Key Laboratory of Optical Information Materials and Technology, Institute for Advanced Materials, South China Academy of Advanced Optoelectronics, South China Normal University, Guangzhou 510006, China;

[2]Guangdong Basic Research Center of Excellence for Structure and Fundamental Interactions of Matter, Guangdong Provincial Key Laboratory of Quantum Engineering and Quantum Materials, School of Physics, South China Normal University, Guangzhou 510006, China

[3]Key Laboratory of Material Modification by Laser, Ion and Electron Beams, (Dalian University of Technology), Ministry of Education, Dalian 116024, China;

[4]College of Smart Materials and Future Energy, Fudan University, Shanghai 200438, China

[5]Ultrafast Electron Microscopy Laboratory, The MOE Key Laboratory of Weak-Light Nonlinear Photonics, School of Physics, Nankai University, Tianjin 300071, China;

[6]School of Materials Science and Engineering & Research Institute of Interdisciplinary Science, Dongguan University of Technology, Dongguan, 523808, China;

[7]School of Materials and Microelectronics, Wuhan University of Technology, Wuhan 430070, China;

[8]Guangdong Provincial Key Laboratory of Rare Earth Development and Application, Guangdong Academy of Sciences, Guangzhou 510650, PR China;

[9]College of Physics and Electronic Engineering, Sichuan Normal University, Chengdu 610068, China;

[10]Academy for Advanced Interdisciplinary Studies, Nankai University, Tianjin 300071, China;

[11]Laboratory of Solid State Microstructures and Innovation Center of Advanced Microstructures, Nanjing University, Nanjing 211102, China.

*Corresponding author E-mail: jiangx@scnu.edu.cn; rcche@fudan.edu.cn; xwfu@nankai.edu.cn; houzp@m.scnu.edu.cn.

Supplementary Note 1 for Micromagnetic simulations

Micromagnetic simulations were performed using the GPU-accelerated simulation package MuMax^3. The simulated geometry was a slab with dimensions of 512 × 512 × 64 nm^3 and a mesh size of 2.0 × 2.0 × 2.0 nm^3. Periodic boundary conditions are applied to emulate large-scale simulations. The total energy of the system includes contributions from exchange interaction, first-order uniaxial magnetic anisotropy, Dzyaloshinskii-Moriya interaction (DMI), demagnetization energy, and Zeeman energy. The initial magnetization process is simulated using magnetic parameters guided by experimental measurements [*Nat. Commun.* 15, 1017 (2024)], including ferromagnetic exchange constant (J), effective magnetic anisotropy constant (K_{eff}), DMI constant (D), and saturation magnetization (M_s). The specific values used are: J = 1.1 pJ/m; K_{eff} = 1.5 × 10^5 J/m^3; D = 0.25 mJ/m^2; M_s= 2.5 × 10^5 A/m. Gilbert damping (α) is set to be 0.1, following the experimentally reported value of 0.075 for Fe_3GaTe_2 at 300 K [*Nano Lett.*, 24, 12204 (2024)]. After confirming consistency between simulation results and experimental observations, we explored how variations in the four parameters affect skyrmion diameter, density, and evolution dynamics (Supplementary Figs. S1-S3).

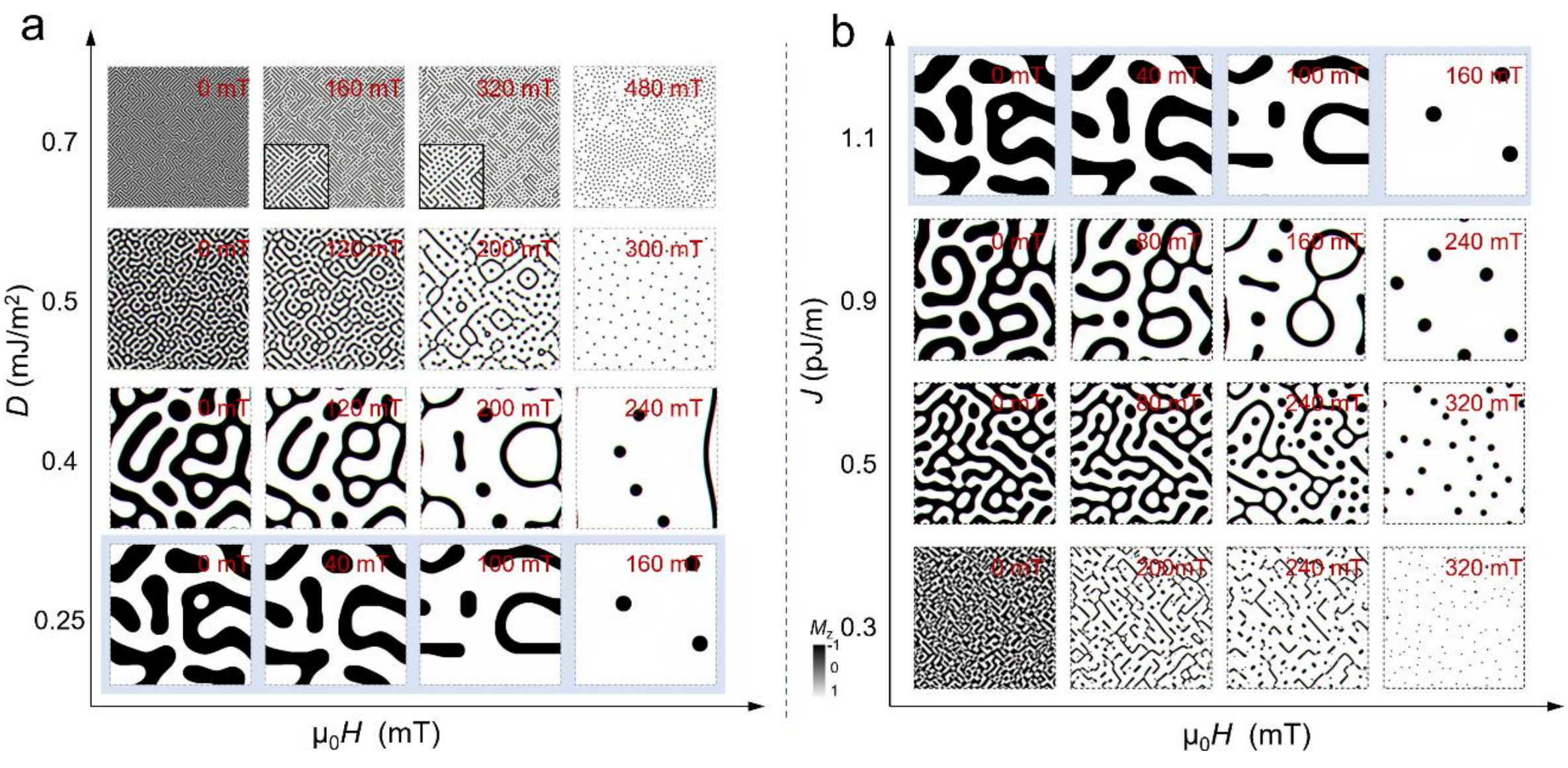

Figure S1. Simulated magnetic field-dependent domain evolution process for varied *D* and *J*. a, Field-dependent domain evolution process for varied D with other parameters fixed. **b,** Field-dependent domain evolution process for varied J with other parameters fixed. The images that are blue marked represent the initial magnetization process simulated using magnetic parameters guided by experimental measurements of $Fe_{3-\delta}GaTe_2$. Magnetization along z-axis (M_z) is represented by regions in white ($+M_z$) and black ($-M_z$).

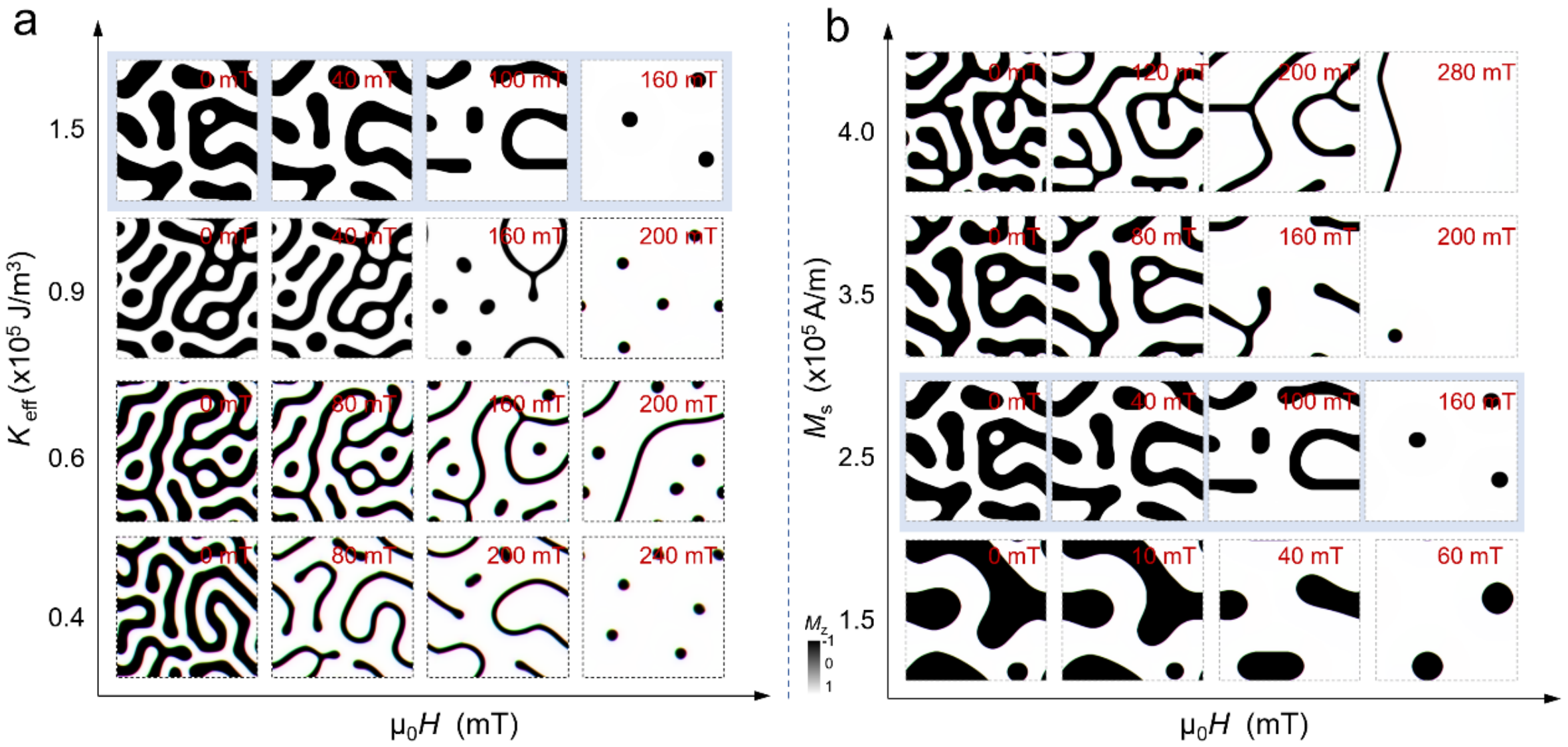

Figure S2. Simulated magnetic field-dependent domain evolution process for varied K_{eff} and M_s. a, Field-dependent domain evolution process for varied K_{eff} with other parameters fixed. **b,** Field-dependent domain evolution process for varied M_s with other parameters fixed. The images that are blue marked represent the initial magnetization process simulated using magnetic parameters guided by experimental measurements of $Fe_{3-\delta}GaTe_2$.

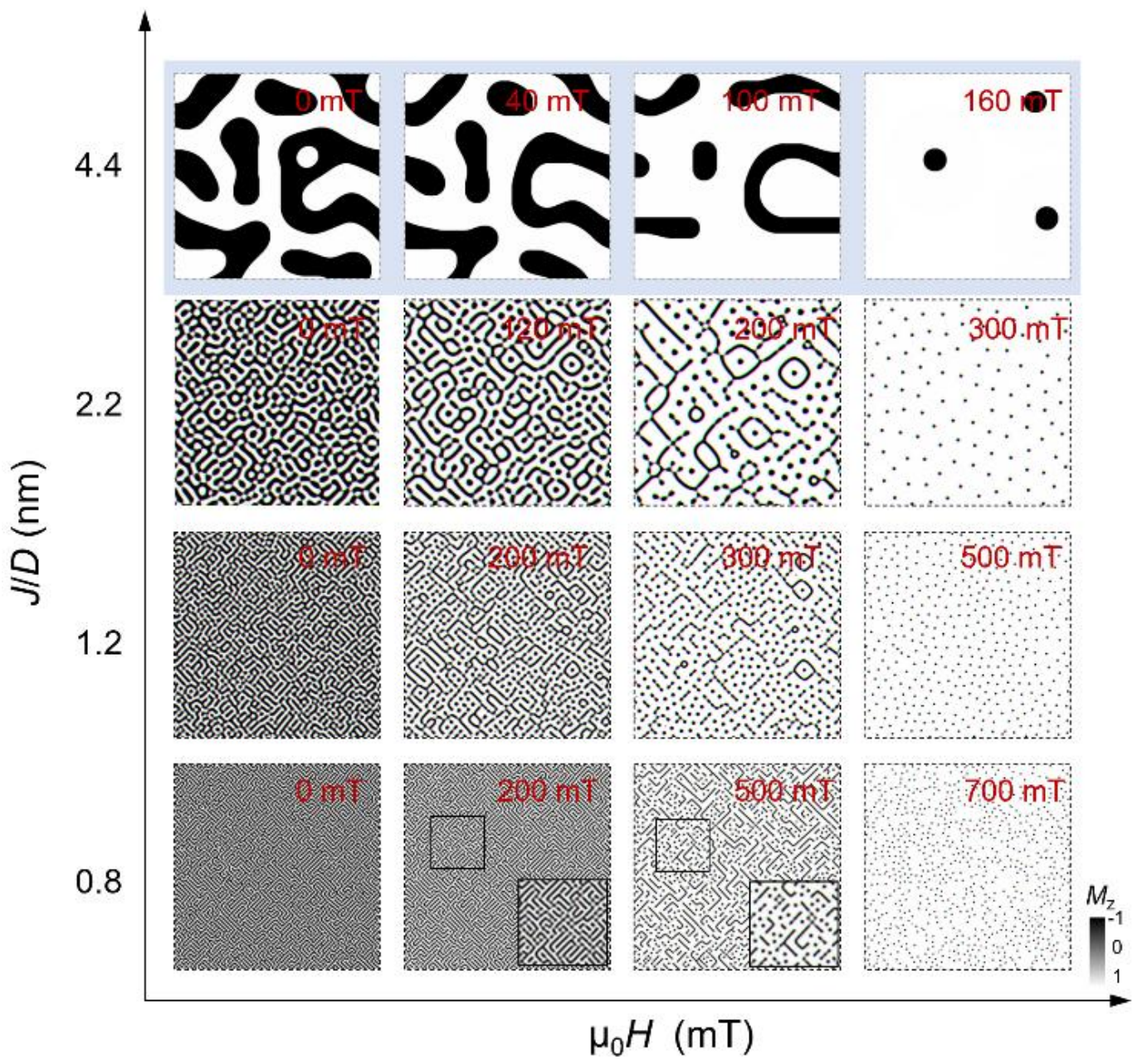

Figure S3. Magnetic field driven evolution of magnetic domains under various J/D ratio. M_s and K_{eff} are fixed to be 2.5×10^5 A/m and 1.5×10^5 J/m^3, respectively. For $J/D = 4.4$ nm, $J = 1.1$ pJ/m, $D = 0.25$ mJ/m^2; $J/D = 2.2$ nm, $J = 1.1$ pJ/m, $D = 0.5$ mJ/m^2; $J/D = 1.4$, $J = 0.7$ pJ/m, $D = 0.5$ mJ/m^2; $J/D = 0.8$ nm, $J = 0.4$ pJ/m, $D = 0.5$ mJ/m^2.

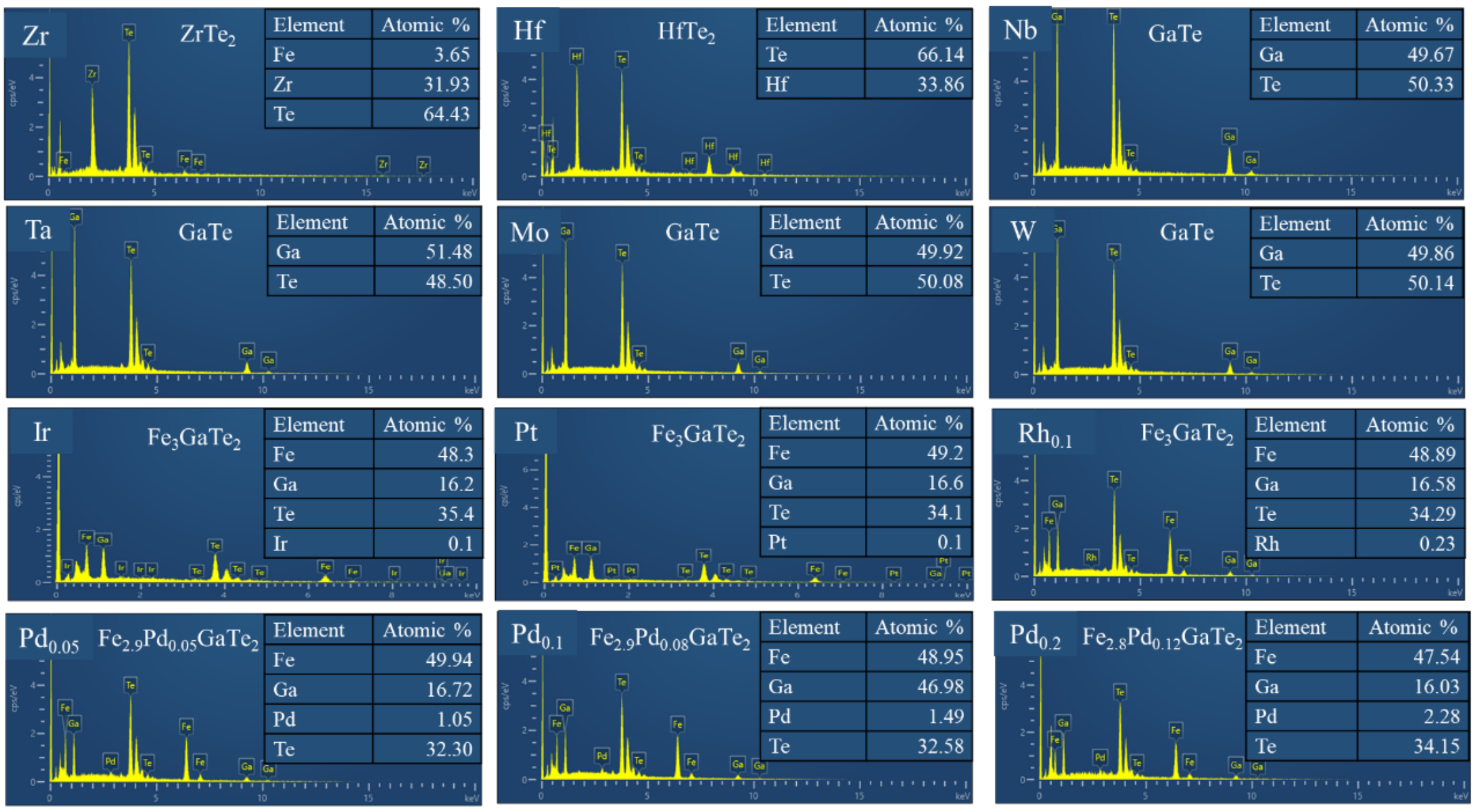

Figure S4. EDX measurements of single crystals. Typical EDX spectrum and quantification results of $Fe_{3-\delta}GaTe_2HTM_x$ (HTM = Zr, Hf, Nb, Ta, Mo, W, Ir, Pt, Rh, Pd) single crystals grown using a Te-flux method.

Molar ratio of Fe: Ga: Te: HTM	HTM	Product
1 : 1 : 2 : 0.1	Zr	$Zr_{0.51}Te$
1 : 1 : 2 : 0.1	Hf	$Hf_{0.51}Te$
1 : 1 : 2 : 0.1	Nb	$Ga_{1.00}Te$
1 : 1 : 2 : 0.1	Ta	$Ga_{1.06}Te$
1 : 1 : 2 : 0.1	Mo	$Ga_{0.99}Te$
1 : 1 : 2 : 0.1	W	$Ga_{0.99}Te$
1 : 1 : 2 : 0.1	Ir	$Fe_{2.98}Ir_{0.006}GaTe_{2.18}$
1 : 1 : 2 : 0.1	Pt	$Fe_{2.96}Pt_{0.006}GaTe_{2.05}$
1 : 1 : 2 : 0.1	Rh	$Fe_{3.05}Rh_{0.013}GaTe_{2.08}$
1 : 1 : 2 : 0.05	Pd	$Fe_{2.90\pm0.04}Pd_{0.05\pm0.010}GaTe_{1.95\pm0.02}$
1 : 1 : 2 : 0.1	Pd	$Fe_{2.93\pm0.04}Pd_{0.08\pm0.009}GaTe_{2.05\pm0.14}$
1 : 1 : 2 : 0.2	Pd	$Fe_{2.83\pm0.02}Pd_{0.12\pm0.011}GaTe_{1.99\pm0.03}$

Table S1. Summary of raw material compositions and the final products of $Fe_{3-\delta}GaTe_2HTM_x$ single crystals grown using a Te-flux method. The listed compositions represent average values obtained by EDX measurements at five distinct positions on each cleaved flake.

Supplementary Note 2 for first-principle calculations

Based on density functional theory (DFT), the Vienna ab initio simulation package (VASP) is used to calculate the forces, electronic structures, and magnetic properties of the non-stoichiometric $Fe_{3-\delta}GaTe_2$ and Pd-intercalated $Fe_{3-\delta}GaTe_2$ (that is, $Fe_{3-\delta}GaTe_2Pd_x$). Exchange-correlation functionals are described by the Perdew-Burke-Ernzerhof (PBE) functional under the generalized gradient approximation (GGA). The strong correlation between the localized *d* orbitals was taken into account by including a Hubbard U term through the GGA + U formulation [*Phys. Rev. B* 74, 125106 (2006)]. An effective U value of 2.5 eV was applied. The interaction between valence electrons and ionic cores is described within the framework of the projector augmented wave (PAW) method. Moreover, vdW interactions are included using the DFT-D3 in all calculations.

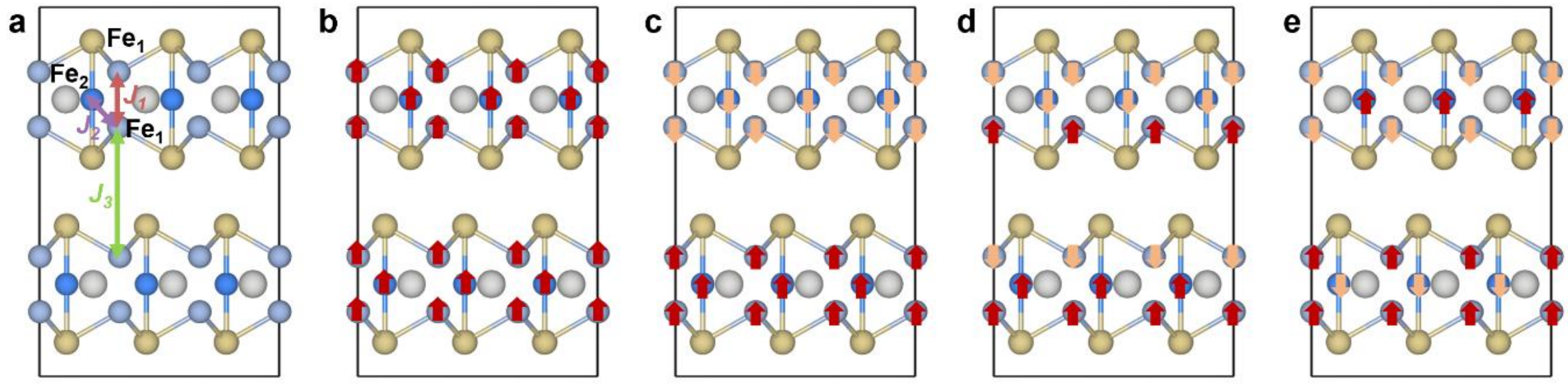

Figure S5. a, Defined exchange parameter. **b-e** Schematic illustration of four magnetic configurations. Red arrows indicate spins pointing upward, whereas pink arrows indicate spins pointing downward. The total energies of the four magnetic states correspond to E_1, E_2, E_3, E_4.

First, the magnetic ground state of $Fe_{2.88}GaTe_2$ and $Fe_{2.88}GaTe_2Pd_{0.05}$ was determined by comparing the energies of four magnetic configurations, as displayed in Figs. S5a-e. We find that FM state (Fig. S5b) is always more stable than the other three states (Figs. S5c-e), suggesting that these compounds exhibit ferromagnetism. Then, we analyzed all the symmetry-inequivalent intralayer Fe_1-Fe_1 bond lengths in both $Fe_{2.88}GaTe_2$ and $Fe_{2.88}GaTe_2Pd_{0.05}$. Specifically, in $Fe_{2.88}GaTe_2$ ($Fe_{2.88}GaTe_2Pd_{0.05}$), Fe_1-Fe_1 distances associated with the nearest-neighbor exchange coupling constants (J_1) range from 2.370 Å to 2.448 Å (from 2.369 Å to 2.455 Å), corresponding to variations of ~0.078 Å (~0.086 Å). To further quantify the impact of these bond-length variations, we calculated J_1 for the shortest and longest Fe_1-Fe_1 distances (denoted as $J_{1\text{-shortest}}$ and $J_{1\text{-longest}}$) in both systems based on four-state energy-mapping method [*Dalton Trans.* 42, 823 (2013)]. For $Fe_{2.88}GaTe_2$, $J_{1\text{-shortest}}$ and $J_{1\text{-longest}}$ are 37.889 meV and 35.040meV, respectively, whereas for $Fe_{2.88}GaTe_2Pd_{0.05}$, they

are 34.554 meV and 30.964 meV. The resulting differences (i.e., $\Delta J_1 = J_{1\text{-shortest}} - J_{1\text{-longest}}$) are 2.849 meV for $Fe_{2.88}GaTe_2$ and 3.590 meV for $Fe_{2.88}GaTe_2Pd_{0.05}$. The relatively small variations suggest that, although the systems lack high crystallographic symmetry, the intralayer Fe_1-Fe_1 bonding environment does not give rise to strongly inequivalent exchange parameters. Therefore, we group the dominant magnetic interactions into nearest-neighbor (J_1), next-nearest-neighbor (J_2), and interlayer (J_3) exchange constants, as schematically illustrated in Fig. S5a.

The Heisenberg Hamiltonian for our calculations is expressed as:

$$H = -J_1 \sum_{i,j} \overrightarrow{S_i} \cdot \overrightarrow{S_j} - J_2 \sum_{k,l} \overrightarrow{S_k} \cdot \overrightarrow{S_l} - J_3 \sum_{m,n} \overrightarrow{S_m} \cdot \overrightarrow{S_n} - K\left(S^Z\right)^2, (1)$$

where S denotes the magnetic moment at the corresponding Fe site, and K is the anisotropic energy parameter. J_1, J_2, and J_3 are obtained from the total energies of one ferromagnetic configuration (schematically illustrated in Fig. S5b, E_1) and three other states (schematically illustrated in Figs. S5c-e, E_2, E_3, E_4) following the classical Heisenberg Hamiltonian, as described below:

$$E_1 = -17J_1S_1^2 - 99J_2S_1S_2 - 17J_3S_1^2 + E_0, (2)$$

$$E_2 = -17J_1S_1^2 - 99J_2S_1S_2 + 17J_3S_1^2 + E_0, (3)$$

$$E_3 = 17J_1S_1^2 - 3J_2S_1S_2 + 17J_3S_1^2 + E_0, (4)$$

$$E_4 = -17J_1S_1^2 + 99J_2S_1S_2 + 17J_3S_1^2 + E_0, (5)$$

where E_0 represents the energy of the nonmagnetic state, S_1 and S_2 represent the spin magnetic moments of Fe_1 and Fe_2 atoms. By combing equations (2)-(5), we can deduce J_1, J_2, and J_3 to be:

$$J_1 = \left(E_3 - \frac{48}{99}E_4 - \frac{51}{99}E_2\right)/34S_1^2, (6)$$

$$J_2 = (E_4 - E_2)/198S_1S_2, (7)$$

$$J_3 = (E_2 - E_1)/34S_1^2. (8)$$

Establishing the structure details of $Fe_{3-\delta}GaTe_2Pd_x$

Previous studies have identified Fe_1 and Fe_2 vacancies in $Fe_{3-\delta}GeTe_2$, which induces a displacement of Fe_2 atoms along the c-axis [*Nat. Commun.* 15, 1017 (2024)]. Such deviations at the Fe_2 sites breaks the centro-symmetry and gives rise to a pronounced Dzyaloshinskii-Moriya interaction (DMI). Using experimentally determined structural parameters (a = 4.05; c = 16.15 nm; space group $P3m1$), we constructed a 3×3 supercell containing two Fe

vacancies and one Pd dopant, corresponding to a composition of $Fe_{2.88}GaTe_2Pd_{0.05}$. We first considered two distinct vacancy configurations located in different layers: (i) Fe_1↑ (upper) + Fe_2(lower), where Fe_1↑(upper) vacancy is located in the upper layer farther from the vdW gap and Fe_2(lower) is located in the lower layer (Fig. S6a) and (ii) Fe_1↓(upper) + Fe_2(lower), where Fe_1↓(upper) vacancy is located in the upper layer closer to the vdW gap and Fe_2 remains in the lower layer (Fig. S6b).

To determine the most favorable Pd doping site, we systematically examined five candidate positions (labeled as I, II, III, IV, and V in Figs. S6a-c) and fully relaxed each structure to its energy minima. Our calculations identified four energetically favorable configurations: Fe_1↑(upper) + Fe_2(lower) + Pd_{II}, Fe_1↑(upper) + Fe_2(lower)+ Pd_{III}, Fe_1↓(upper) + Fe_2(lower) + Pd_{II} and Fe_1↓(upper) + Fe_2(lower) + Pd_{III}. All these configurations placed Pd within the center of vdW gap, in agreement with TEM observations (Fig. 2b in the main text).

We then calculated the ferromagnetic exchange constant (J) and DMI constant (D) for both $Fe_{2.88}GaTe_2Pd_{0.05}$ and its corresponding host $Fe_{2.88}GaTe_2$ (that is, with Pd removed but the lattice kept identical to $Fe_{2.88}GaTe_2Pd_{0.05}$) for each of the four low-energy configurations. For Fe_1↑(upper) + Fe_2(lower) + Pd_{III} and Fe_1↓(upper) + Fe_2(lower) + Pd_{II} configurations, Pd intercalation raised J, inconsistent with our experimental observations. Thus, we excluded them from further consideration. In Fe_1↑(upper) + Fe_2(lower) + Pd_{II}, the calculated DMI vector pointed the out-of-plane direction, which contradicts the experimentally observed in-plane DMI direction, thus discarding this structure as well. As a result, we retained one configuration, that is, Fe_1↓(upper) + Fe_2(lower) + Pd_{III} (Supplementary Fig. S6c). The calculated Pd position is consistent with our atomic-scale scanning transmission electron microscopy (STEM) results in Fig. 2b in the main text, thus validating our calculations.

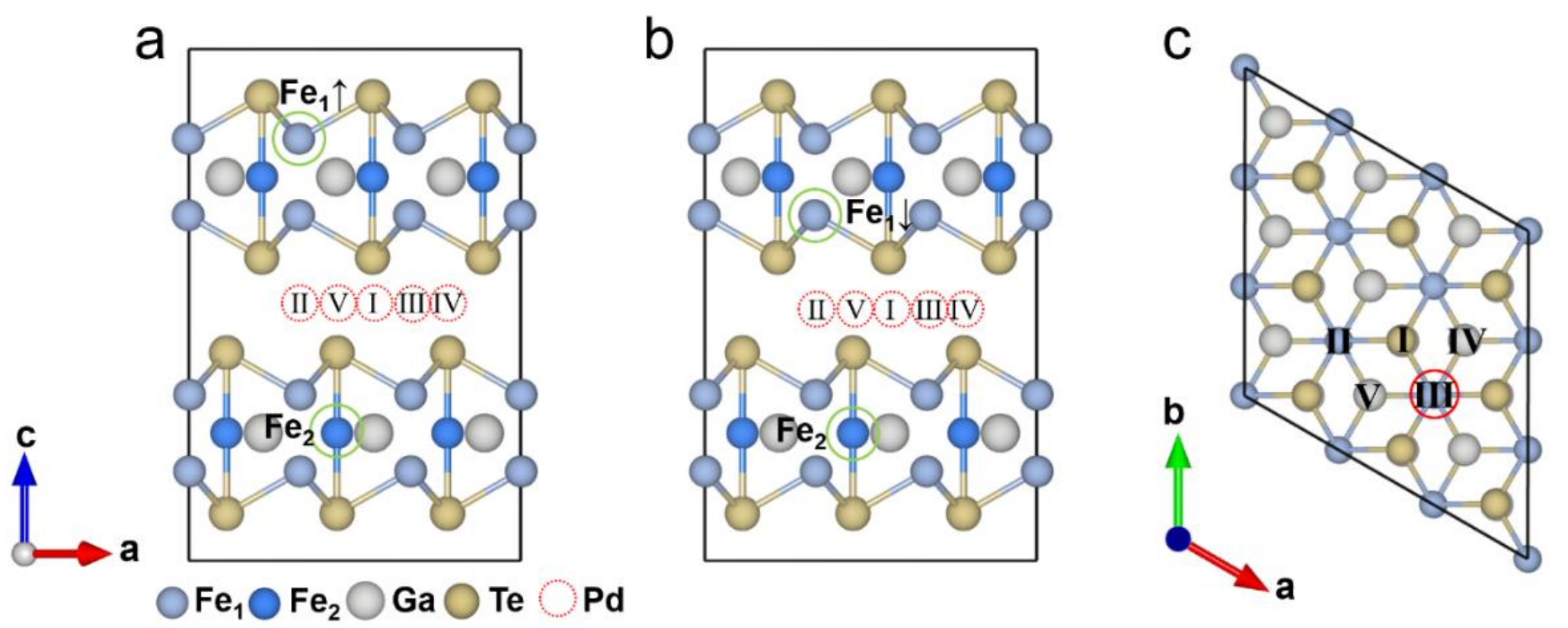

Figure S6. Structure configuration of $Fe_{2.88}GaTe_2$ and $Fe_{2.88}GaTe_2Pd_{0.05}$. Specifically, **a,** Fe_1↑(upper) + Fe_2(lower), in which Fe_1 is located in the upper layer farthest from the vdW

gap and Fe_2 in the lower layer. **b,** $Fe_1\downarrow$(upper) + Fe_2(lower), where Fe_1 is located in the upper layer closest to the vdW gap and Fe_2 remains in the lower layer. **c,** Five Pd candidate positions (labeled as I, II, III, IV, and V). Only $Fe_1\downarrow$(upper) + Fe_2(lower) + Pd_{III} is used for detailed study are enclosed by red dashed circles.

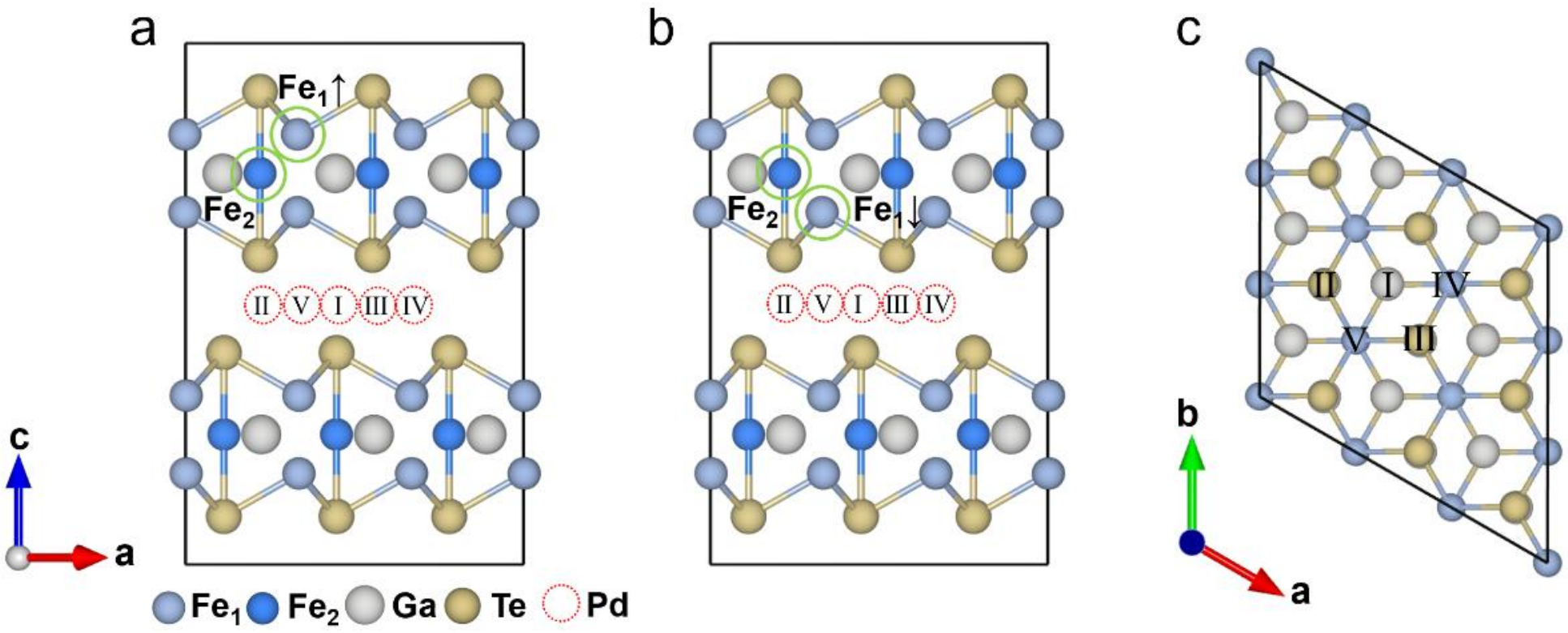

Figure S7. a, $Fe_1\uparrow$(upper) + Fe_2(upper), in which Fe_1 is located in the upper layer farthest from the vdW gap and Fe_2 in the upper layer. **b,** $Fe_1\downarrow$(upper) + Fe_2(upper), where Fe_1 is located in the upper layer closest to the vdW gap and Fe_2 remains in the upper layer. **c,** Five Pd candidate positions (labeled as I, II, III, IV, and V).

We further considered two distinct vacancy configurations located in the same layers: (i) $Fe_1\uparrow$(upper) + Fe_2(upper), where $Fe_1\uparrow$(upper) is located in the upper layer farther from the vdW gap and Fe_2 (upper) is located in the upper layer (Fig. S7a) and (ii) $Fe_1\downarrow$(upper) + Fe_2(upper), where $Fe_1\downarrow$(upper) is located in the upper layer closer to the vdW gap and Fe_2 (upper) is located in the upper layer (Fig. S7b). For each vacancy configuration, we also examined five possible Pd intercalation sites (denoted as I, II, III, IV, and V) (Fig. S7c). However, as discussed below, these configurations do not yield results consistent with experimental observations.

The optimized results show that, for both the $Fe_1\uparrow$(upper) + Fe_2(upper) and $Fe_1\downarrow$(upper) + Fe_2(upper) vacancy configurations, the Pd atom is stably located within the middle position of the vdW gap only when occupying site IV or V. In contrast, for site II, the Pd atom shifts towards a neighboring Te atom, while for sites I and III, it migrates into the lower Fe-containing layer rather than locating within the vdW gap. We then calculated the nearest-neighbor exchange coupling constants (J_1) for both $Fe_{2.88}GaTe_2$ and $Fe_{2.88}GaTe_2Pd_{0.05}$ in the $Fe_1\uparrow$(upper) + Fe_2(upper) + Pd_{IV}, $Fe_1\downarrow$(upper) + Fe_2(upper) + Pd_{IV}, $Fe_1\uparrow$(upper) + Fe_2(upper) + Pd_V, and $Fe_1\downarrow$(upper) + Fe_2(upper) + Pd_V configurations. Pd intercalation in these configurations leads to an enhancement of J_1, which is in contradiction with the experimentally observed reduction of exchange coupling.

Vacancy configuration	Pd site	Structure	J_1 (meV)
		$Fe_{2.88}GaTe_2$	27.541
Fe_1↑(upper) + Fe_2 (upper)	IV	$Fe_{2.88}GaTe_2Pd_{0.05}$	29.200
	V	$Fe_{2.88}GaTe_2Pd_{0.05}$	27.815
		$Fe_{2.88}GaTe_2$	27.541
Fe_1↓(upper) + Fe_2 (upper)	IV	$Fe_{2.88}GaTe_2Pd_{0.05}$	29.097
	V	$Fe_{2.88}GaTe_2Pd_{0.05}$	28.621

Table S2. Calculated nearest-neighbor exchange coupling constants (J_1) for both $Fe_{2.88}GaTe_2$ and $Fe_{2.88}GaTe_2Pd_{0.05}$ in the Fe_1↑(upper) + Fe_2(upper) + Pd_{IV}, Fe_1↓(upper) + Fe_2(upper) + Pd_{IV}, Fe_1↑(upper) + Fe_2(upper) + Pd_V, and Fe_1↓(upper) + Fe_2(upper) + Pd_V configurations.

Based on these results, we conclude that the energetically favorable Pd intercalation site is sensitive to the Fe-vacancy configuration, and only vacancy configurations in which Fe vacancies are distributed across different layers reproduce the experimentally observed Pd position and magnetic trends. In subsequent calculations, we focus on the Fe_1↓ + Fe_2 + Pd_{III} configuration. The calculations details are provided below. The plane-wave expansion of the energy cutoff was set to be 500 eV, and the Brillouin Zone was sampled by Γ-centered k-point mesh of 0.02 $Å^{-1}$ (0.03 $Å^{-1}$) for $Fe_{2.88}GaTe_2$ ($Fe_{2.88}GaTe_2Pd_{0.05}$). Lattice constants and atomic positions were fully relaxed until the force on each atom was less than 0.01 eV/Å (0.02 eV/Å) and the energy convergence criterion was less than 10^{-6} eV (10^{-5} eV) for $Fe_{2.88}GaTe_2$ ($Fe_{2.88}GaTe_2Pd_{0.05}$) structural optimization.

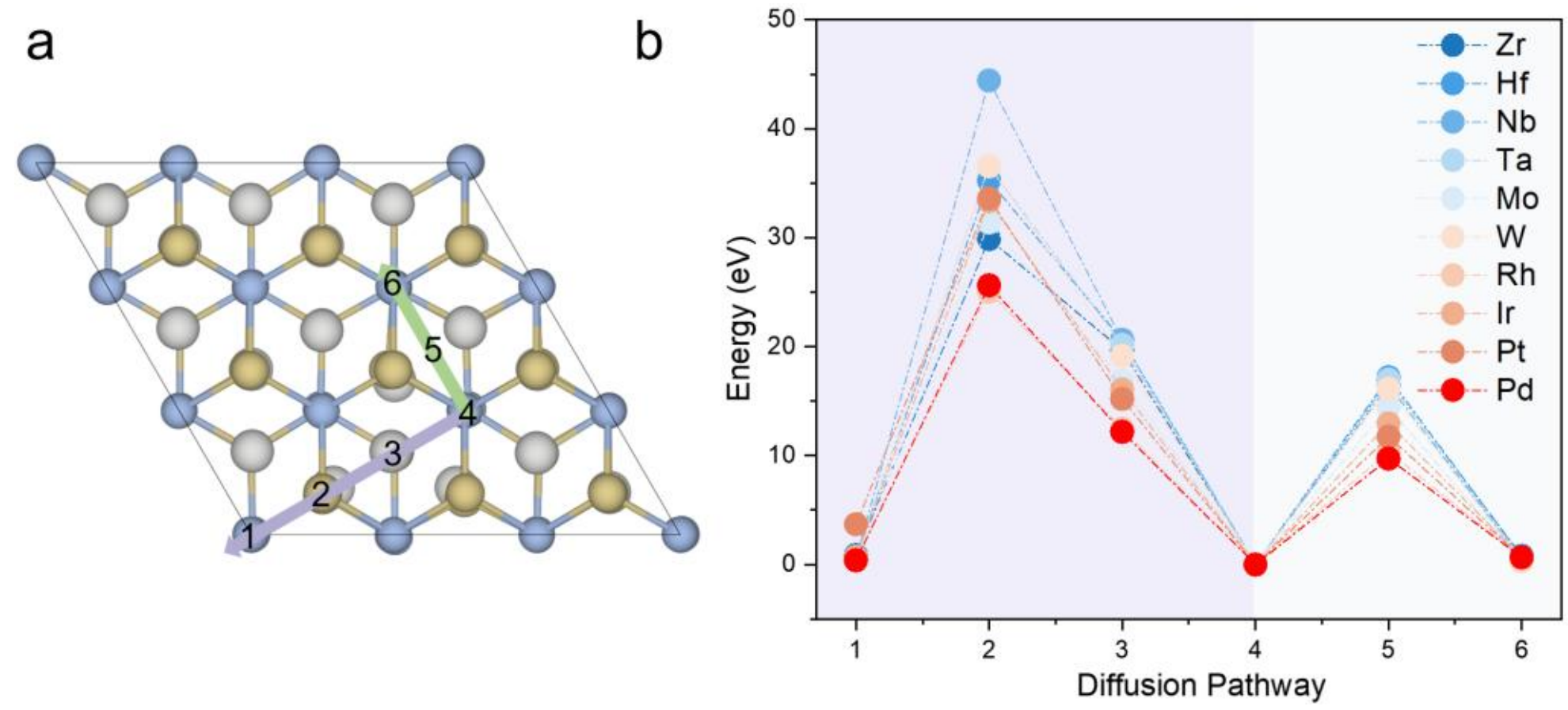

Figure S8. **Migration energy of various heavy transition metal atoms in the vdW gap of $Fe_{2.88}GaTe_2$. a,** Diffusion pathway of heavy metal atoms (Zr, Hf, Nb, Ta, Mo, W, Ir, Pt, Rh, Pd) within the vdW gap of FGT and **b,** the corresponding energy profile. The energy of the metal atom at the 4-site configuration is set to zero as the baseline reference.

Elements	Zr	Hf	Nb	Ta	Mo	W	Ir	Pt	Rh	Pd
Cohesive Energy (eV/atom)	6.25	6.44	7.57	8.10	6.82	8.90	6.94	5.84	5.75	3.89

Table S3. Cohesive energies of various heavy transition metal atoms (Zr, Hf, Nb, Ta, Mo, W, Ir, Pt, Rh, Pd). Among the examined heavy transition metal elements, Pd exhibits the lowest cohesive energy, suggesting that it can more easily dissociate into isolated atoms, thereby facilitating subsequent intercalation into the van der Waals (vdW) gaps.

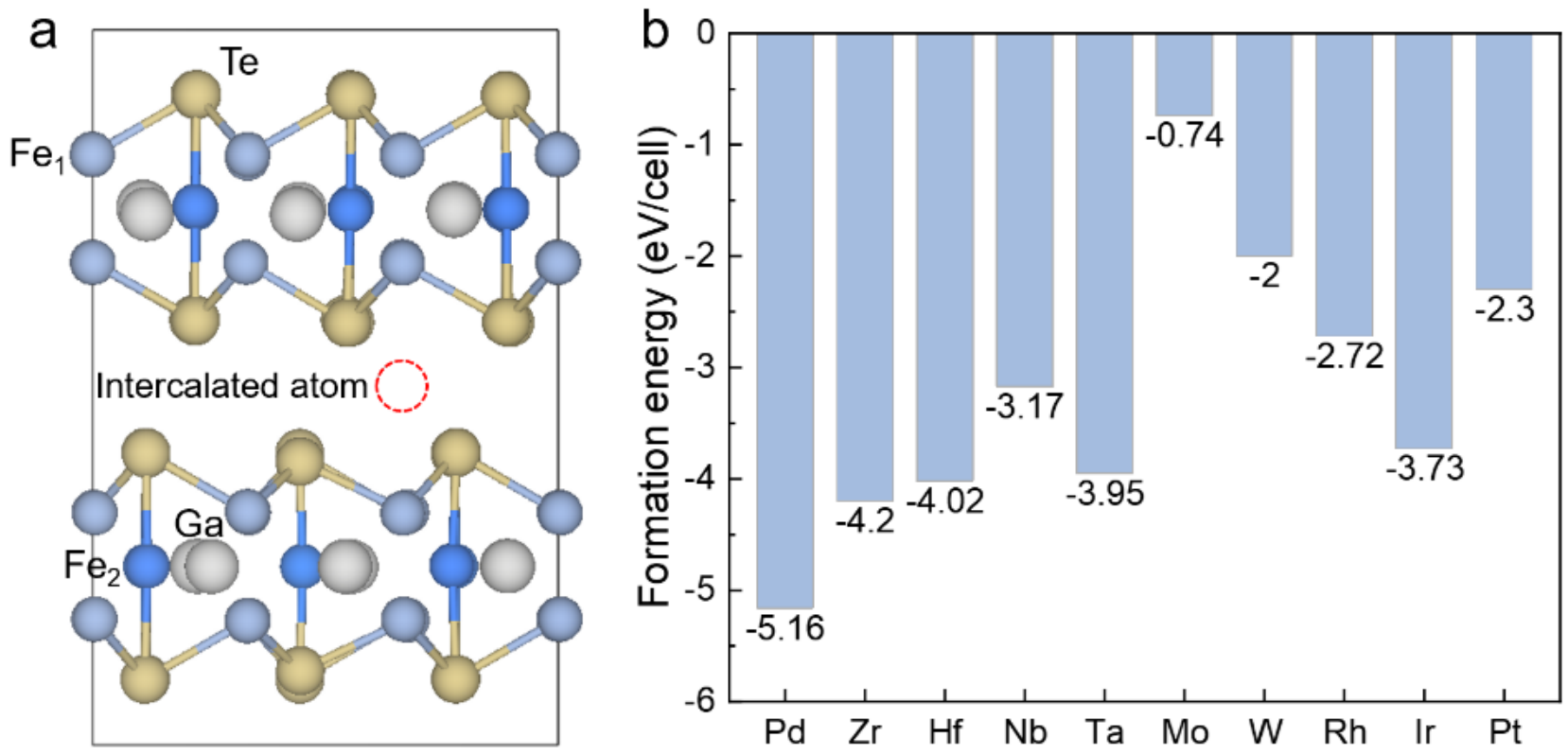

Figure S9. a, Structure configuration of intercalated atoms in the gaps of the $Fe_{2.88}GaTe_2$. **b,** The calculated formation energies of heavy metal atoms (Zr, Hf, Nb, Ta, Mo, W, Ir, Pt, Rh, Pd)-intercalated gaps of the $Fe_{2.88}GaTe_2$. Pd shows the lowest formation energy, indicating that its intercalation is the most thermodynamically favorable.

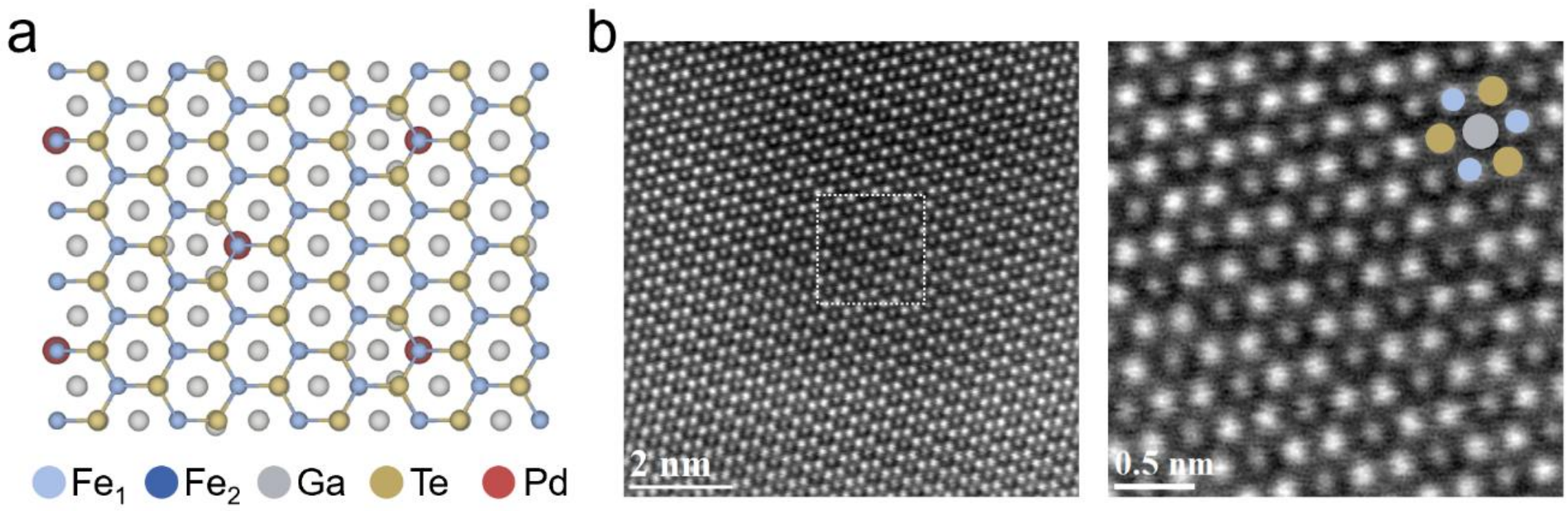

Figure S10. Structural characterization of Pd-doped $Fe_{3-\delta}GaTe_2$ along the [001] zone axis. **a,** Schematic diagram of the crystal structure of Pd-doped $Fe_{3-\delta}GaTe_2$ imaged along the [001] zone axis. Left panel of **b,** High-resolution STEM-HAADF image acquired along the [001] zone axis. The right panel shows a magnified view of the region outlined by the white dashed box in the left panel.

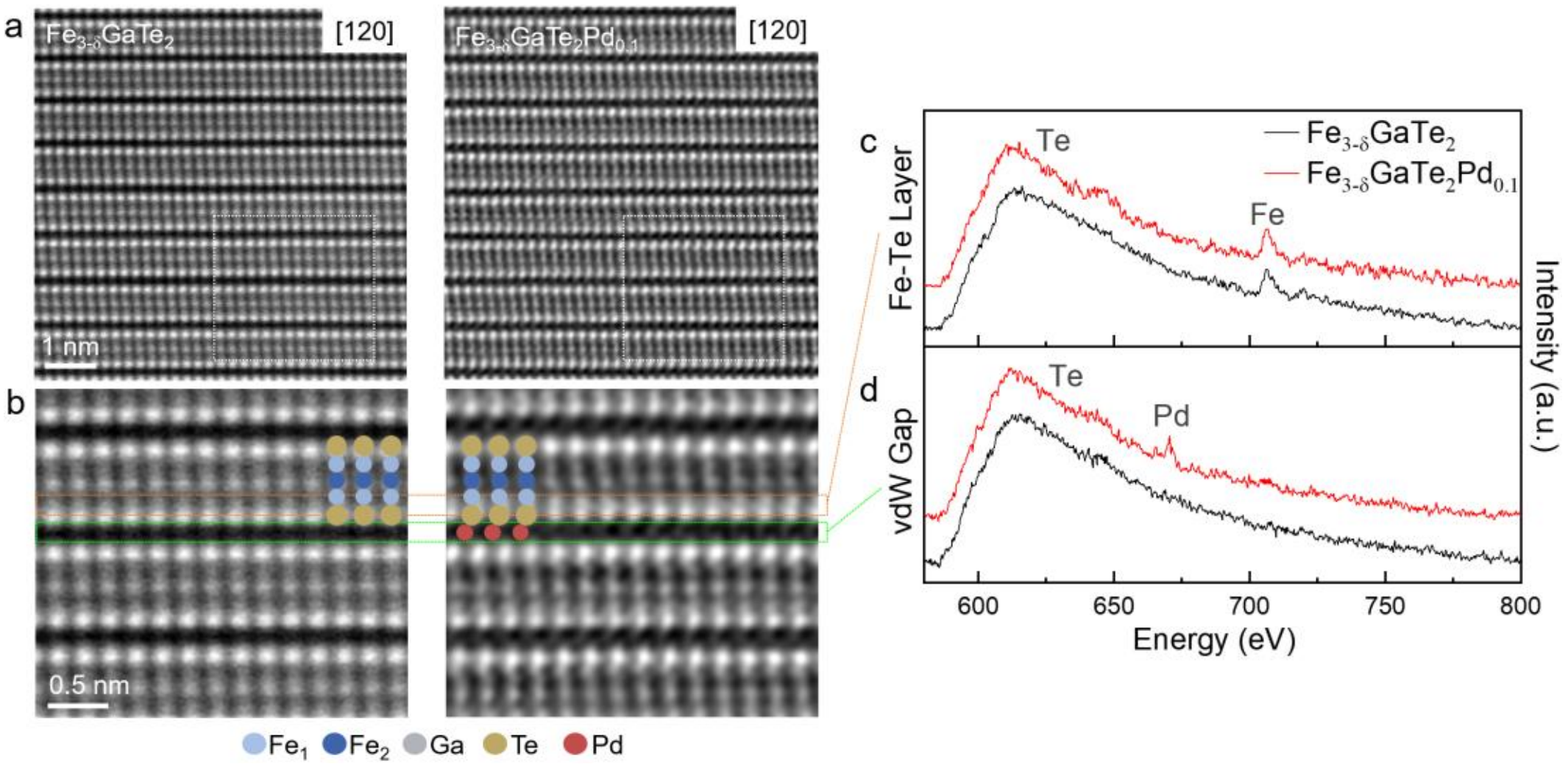

Figure S11. Atomic images and EELS spectrum of $Fe_{3-\delta}GaTe_2$ and $Fe_{3-\delta}GaTe_2Pd_{0.1}$. a, Annular bright-field (ABF) image of taken along the [120] zone axis. Scale bars represent 1 nm. **b**, Enlarged atomic images corresponding to the regions marked in panel (a). The elements are represented as follows: Fe_1 (light blue), Fe_2 (dark blue), Te (yellow), Pd (red). Scale bars represent 0.5 nm. **c**, **d** EELS spectrum obtained from different layers, as indicated by the boxes marked in panel **b**. The EELS spectra show distinct Fe-related signals from Fe-Te layer in both $Fe_{3-\delta}GaTe_2$ and $Fe_{3-\delta}GaTe_2Pd_{0.1}$ (Fig. S11c). In contrast, a distinct Pd-related signal is detected within the vdW gaps of $Fe_{3-\delta}GaTe_2Pd_{0.1}$, while no Fe-related signal is observed within the vdW gaps of either compound (Fig. S11d). These results demonstrate that the vdW gaps in $Fe_{3-\delta}GaTe_2Pd_{0.1}$ are occupied solely by Pd atoms, with no evidence of Fe self-intercalation.

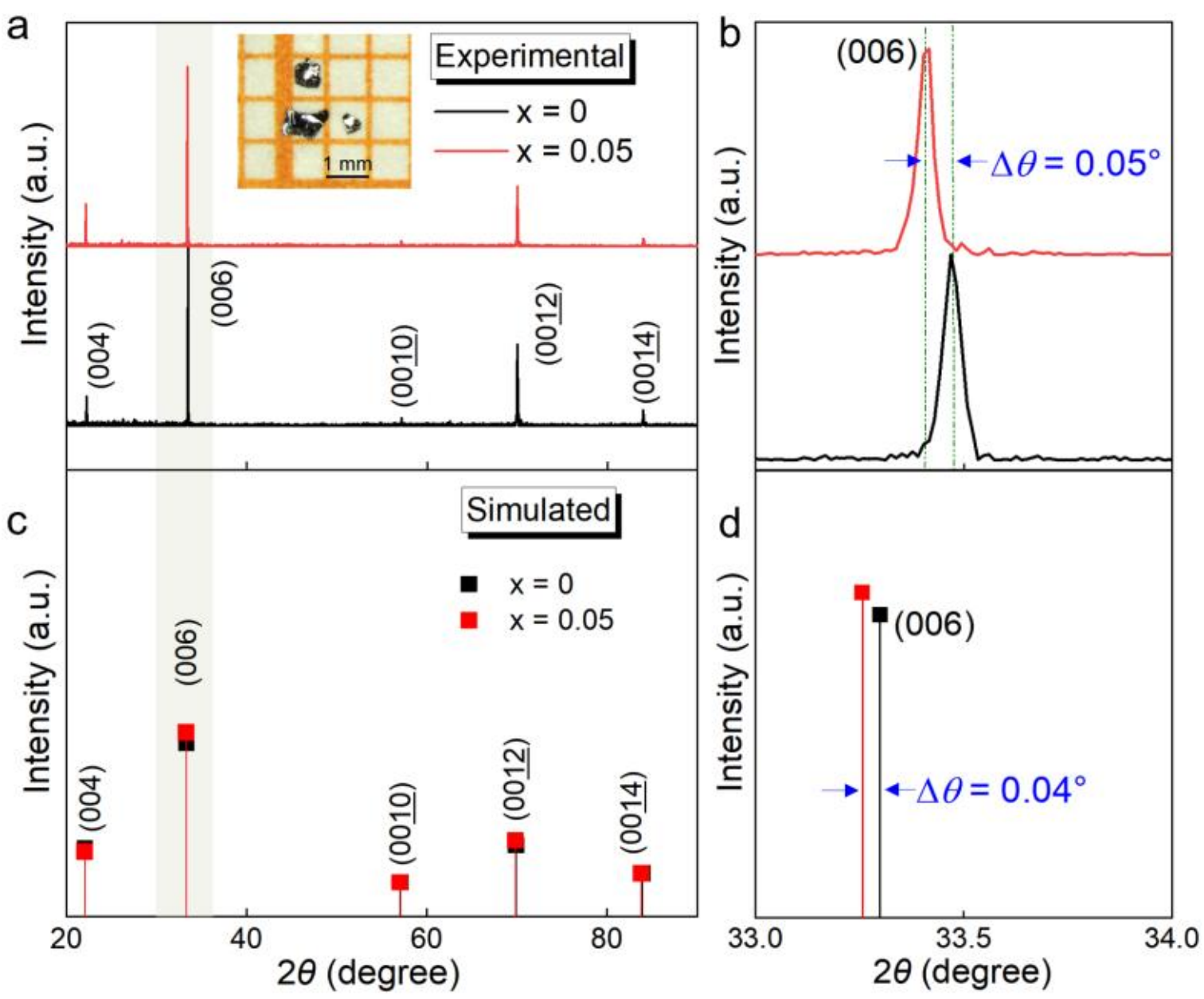

Figure S12. Experimental and simulated X-ray diffraction for $Fe_{3-\delta}GaTe_2$ and Fe_{3-}

$_{\delta}$**GaTe$_2$Pd$_{0.05}$.** After Pd intercalation, no additional diffraction peaks were observed along the (00L) direction, while the strongest Bragg peaks ($2\theta = 33.4°$) exhibited a slight shift of ~0.05° towards lower angles. This behavior is consistent with the XRD patterns simulated on the basis of the STEM-derived structural model, further confirming the reliability of the structural model established from the STEM results. Inset shows an optical image of single crystals of $Fe_{3-\delta}GaTe_2Pd_{0.05}$ with typical lateral size on the order of 0.5-1 mm.

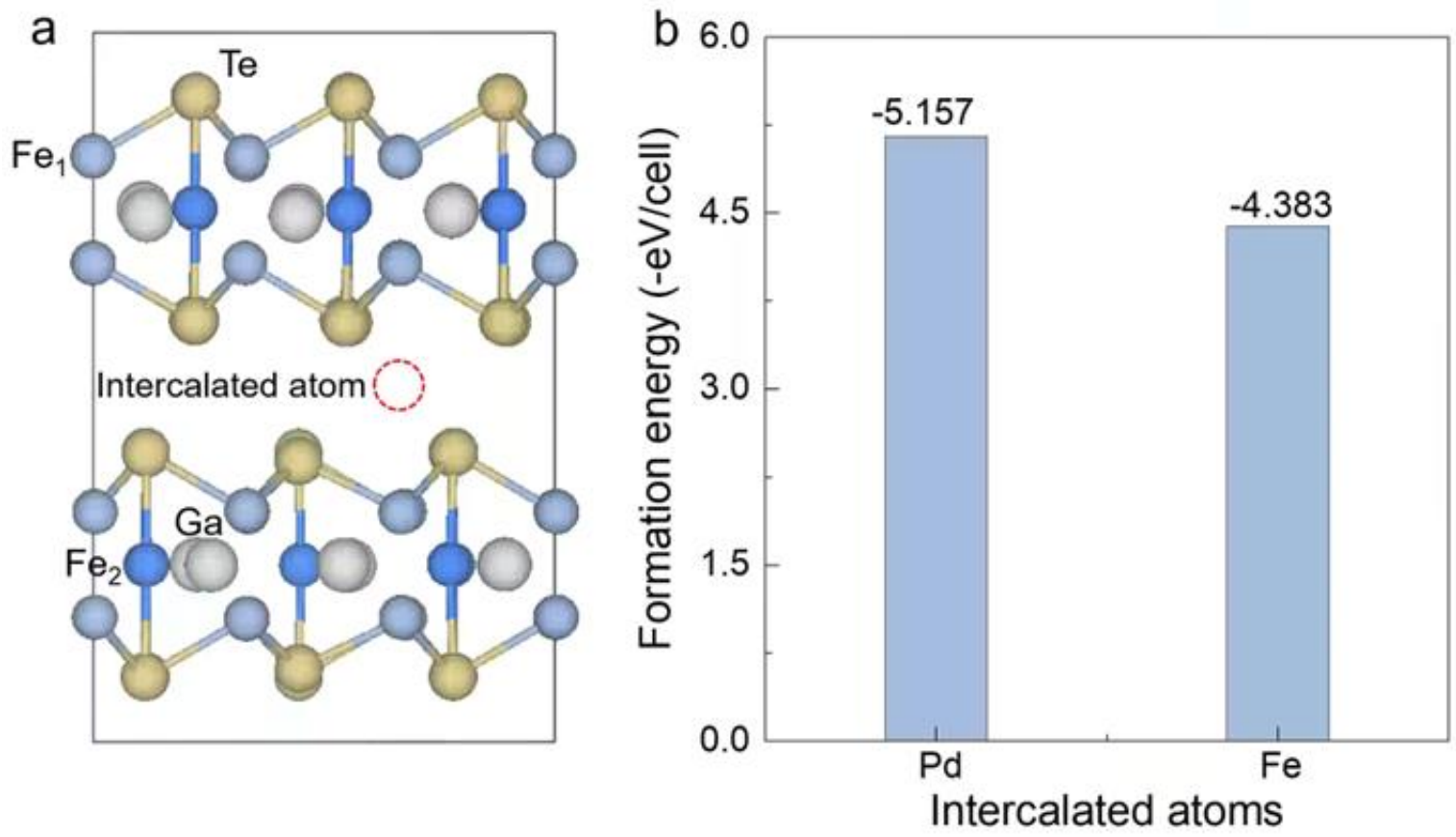

Figure S13. a, Structure configuration of intercalated atoms in the gaps of the $Fe_{2.88}GaTe_2$. **b,** The calculated formation energies of Fe- and Pd-intercalated gaps of the $Fe_{2.88}GaTe_2$. The calculated formation energies are -5.157 eV/cell for Pd intercalation and -4.383 eV/cell for Fe intercalation, indicating that Pd is energetically more favorable for occupying the vdW gaps of $Fe_{2.88}GaTe_2$. These results theoretically confirm that the intercalated atoms in $Fe_{3-\delta}GaTe_2Pd_{0.1}$ are Pd atom rather than Fe atom.

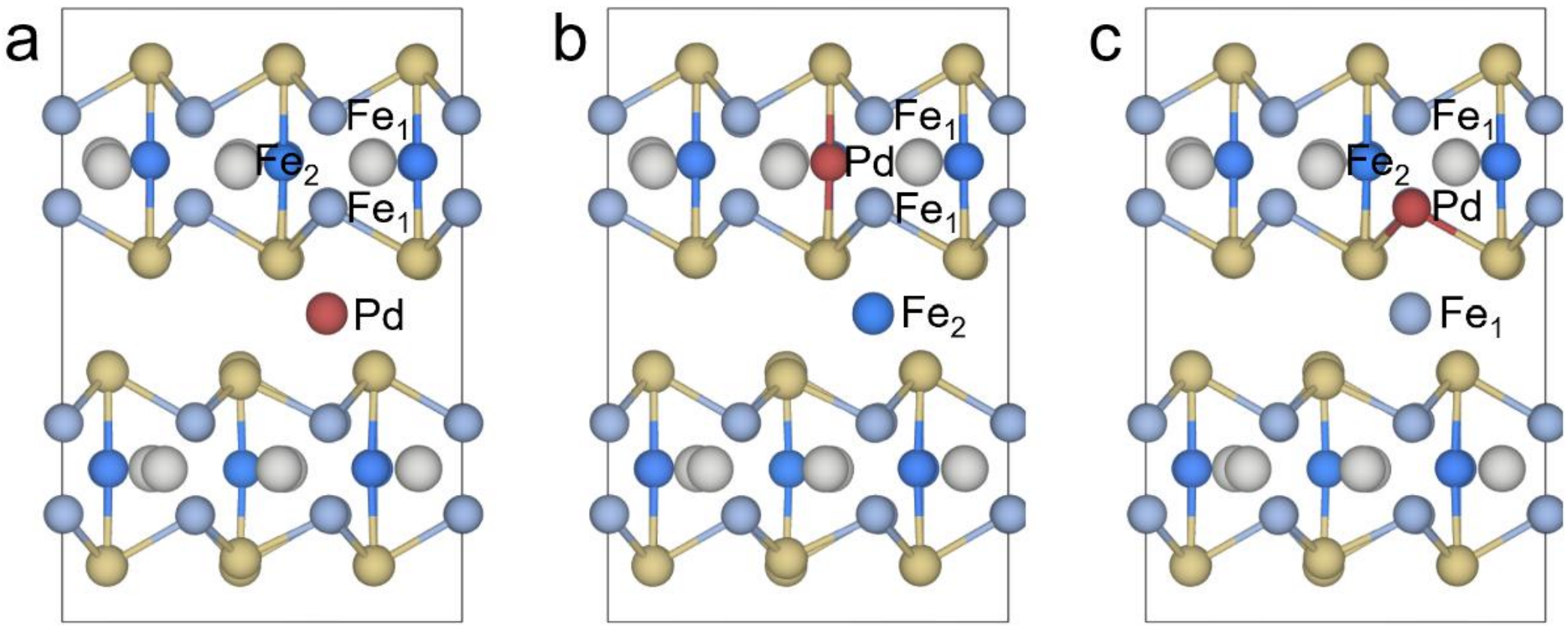

Figure S14. Schematic diagram of various atomic configurations in $Fe_{2.88}GaTe_2Pd_{0.05}$: **a,** Pd intercalated in the vdW gap. **b,** Pd substituting Fe_2 site, and **c,** Pd substituting Fe_1 site. The calculated total energies of these configurations are: -537.046 eV, -535.914 eV, -535.782 eV,

respectively. These results indicate that Pd prefers to intercalate into the vdW gap rather than substitute Fe atoms in $Fe_{2.88}GaTe_2Pd_{0.05}$.

Structure	Lattice (Å)		d (Å)					
	a* = *b	***c***	**Fe_1-Fe_1**	**Fe_1-Fe_2**	**Fe_2-Ga**	**Fe_2-Te**	**Fe_1-Te**	**Fe_1-Ga**
$Fe_{2.88}GaTe_2$	4.0145	16.0001	2.4098	2.606	2.3405	2.5293	2.6632	2.6683
$Fe_{2.88}GaTe_2Pd_{0.05}$	4.0165	16.0047	2.4060	2.6064	2.3412	2.5301	2.6686	2.6029

Table S4. After structural relaxation based on first-principle calculations, the optimized lattice constants, average bond lengths of Fe_1-Fe_1, Fe_1-Fe_2, Fe_2-Ga, Fe_2-Te, Fe_1-Te, and Fe_1-Ga in $Fe_{2.88}GaTe_2$ and $Fe_{2.88}GaTe_2Pd_{0.05}$, respectively. The results demonstrate that Pd-intercalation slightly changes the structure parameters of $Fe_{2.88}GaTe_2$.

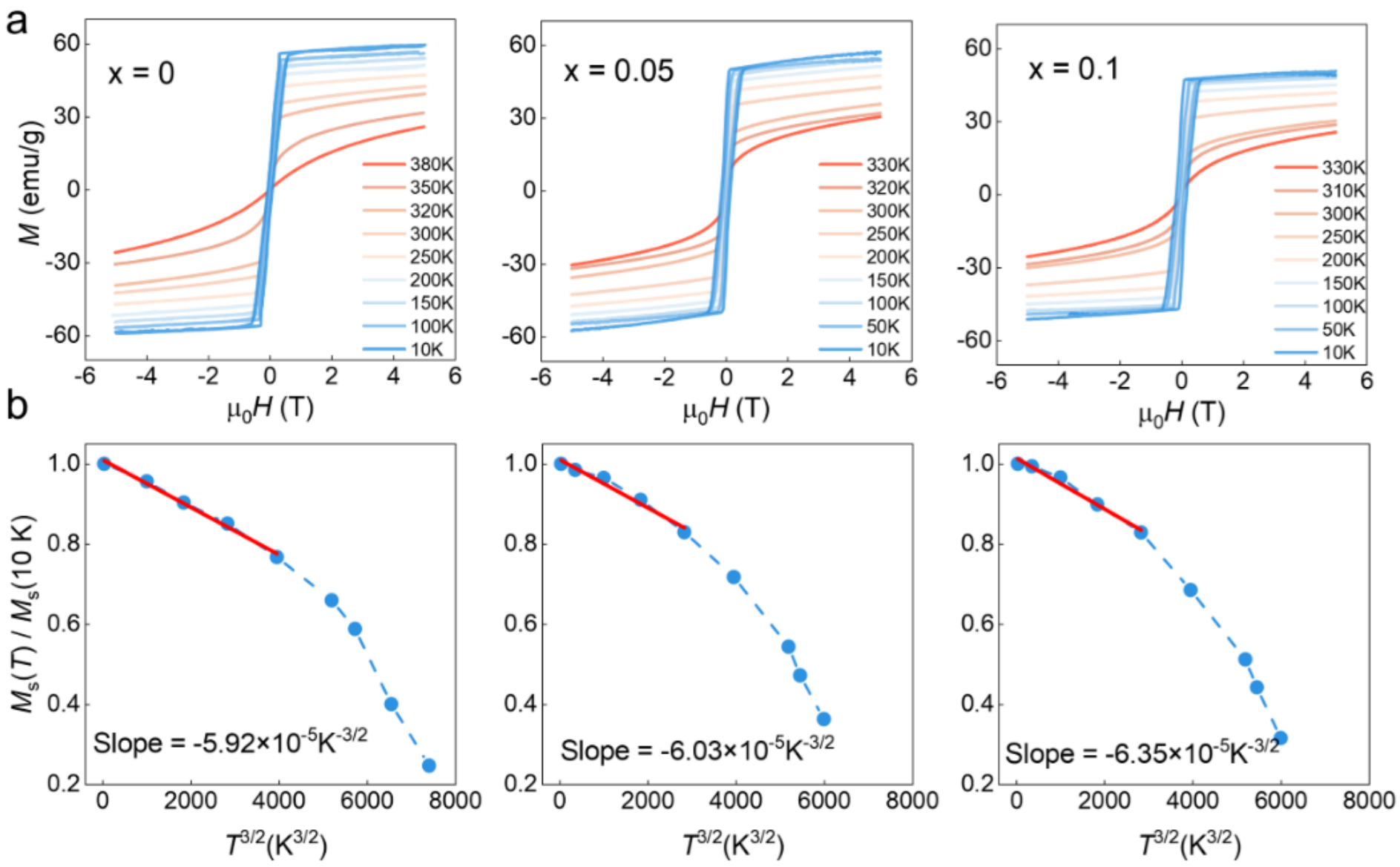

Figure S15. **Establishing saturation magnetization (M_s) and exchange constant (J). a,** Magnetic field dependence of magnetization (M) at various temperatures (T) ranging from 380 K to 10 K for $Fe_{3-\delta}GaTe_2Pd_x$ with x = 0, 0.05 and 0.1. **b,** Normalized saturation magnetization $M_s(T)$ / M_s(10 K) versus $T^{3/2}$ for $Fe_{3-\delta}GaTe_2Pd_x$ with x = 0, 0.05 and 0.1. The red line indicates the linear fitting using the Bloch law [*Nat. Phys.* 11, 825-829 (2015); *Phys. Rev. B*. 99, 094409 (2019); *Nat. Commun*. 15 4472 (2024)]:

$$\frac{M_s(T)}{M_s(10\ \mathrm{K})} = 1\text{-}bT^{3/2}, \qquad (9)$$

where $M_s\,(10\ \mathrm{K})$ is the saturation magnetization at 10 K; b is a spin-wave related coefficient that is defined as:

$$b = \zeta\left(\frac{3}{2}\right)\frac{g u_B}{M_s(10\ \mathrm{K})}\left(\frac{k_B}{4\pi\lambda_{spin}(10\ \mathrm{K})}\right)^{3/2}, \qquad (10)$$

where $\zeta(\frac{3}{2})$ is the Riemann zeta function and is equal to 2.612; g is the Landé g-factor and is equal to 2; u_B is the Bohr magneton and is equal to 9.274×10^{-24} J/T; k_B is the Boltzmann constant and is equal to 1.38×10^{-23} J/K; $\lambda_{spin}(10\ K)$ is the spin-wave stiffness constant at 10 K. By combining the equations (9) and (10), we can obtain b and $\lambda_{spin}(10\ K)$ for different Pd contents. For x = 0, 0.05, and 0.1, the values of b are $5.92\times10^{-5}\ K^{-3/2}$, $6.03\times10^{-5}\ K^{-3/2}$, and $6.35\times10^{-5}\ K^{-3/2}$; $\lambda_{spin}(10\ K)$ takes the values 1.81×10^{-22} J nm^2, 1.93×10^{-22} J nm^2, and 1.94×10^{-22} J nm^2. Moreover, the exchange constant at 10 K, namely J (10 K), is calculated to be 1.89 pJ/m, 1.79 pJ/m, and 1.70 pJ/m through the expression below:

$$J\,(10\ K) = \frac{M_s(10\ K)\,\lambda_{spin}\,(10\ K)}{2gu_B}. \qquad (11)$$

The temperature-dependent exchange stiffness constant $J\,(T)$ can be further derived using the following relation:

$$J\,(T) \cong J\,(10\ K)\left[\frac{Ms\,(T)}{Ms\,(10\ K)}\right]^{1+\gamma}, \qquad (12)$$

where the exponent γ is equal to 1 under the mean field regime. Accordingly, at 300 K, the exchange stiffness constant $J\,(300\ K)$ is estimate to be 0.82 pJ/m, 0.53 pJ/m, and 0.45 pJ/m for x = 0, 0.05, and 0.1, respectively.

According to equation (12), we can further find that $J\,(T)$ scales with the temperature-dependent magnetization $M_s\,(T)$. Since $M_s\,(T)$ decreases monotonically with increasing T and vanishes at Curie temperature (T_C), $J\,(T)$ also reduces accordingly, reaching zero at T_C.

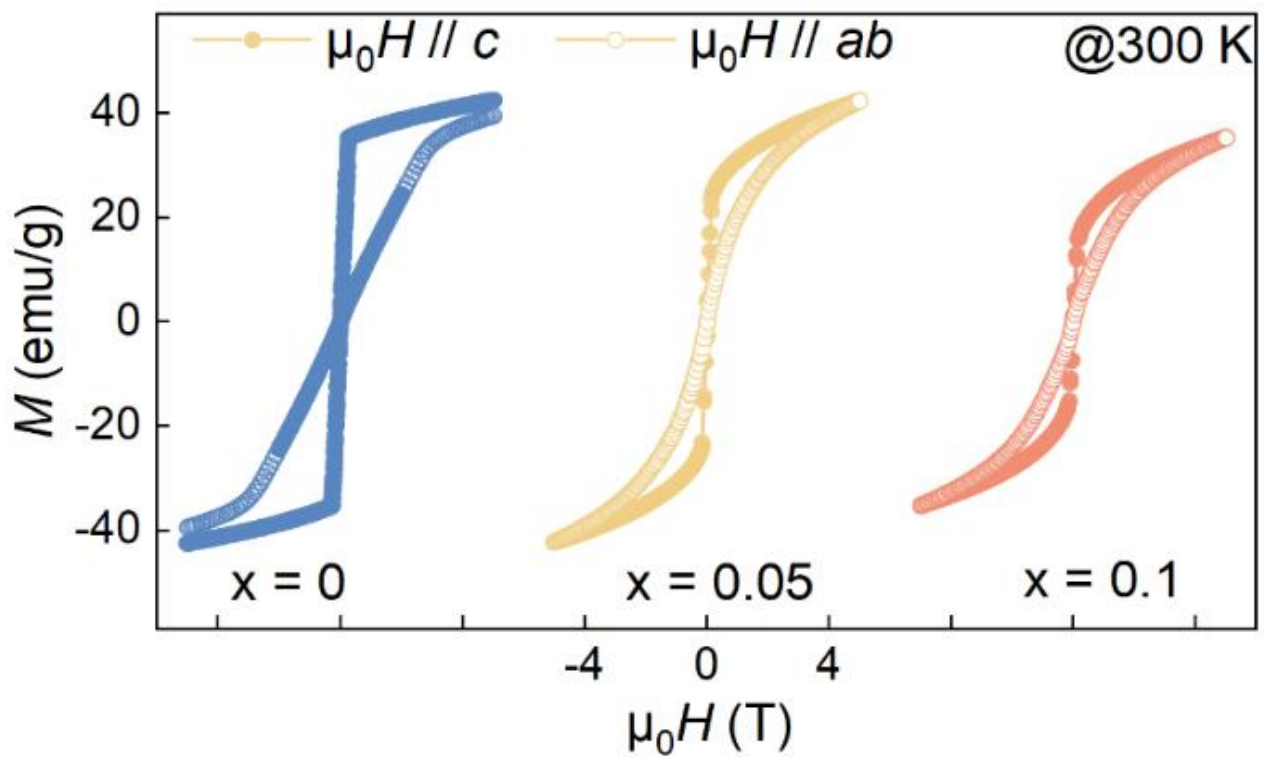

Figure S16. Establishing magnetic anisotropy (K_{eff}). Magnetic field-dependent magnetization (M) curves of $Fe_{3-\delta}GaTe_2Pd_x$ single crystals with x = 0 (blue), 0.05 (yellow), 0.1 (red) for $\mu_0H//c$ (filled circles) and μ_0H // ab (empty circles) at 300 K. The magnetic anisotropy (K_{eff}) was established by calculating the area difference between magnetization curves along the c axis and ab plane:

$$K_{\mathrm{eff}} = \int_0^{M_s} [H_{\perp}(M) - H_{\parallel}(M)]\, \mathrm{d}M \ , \qquad (13)$$

where $H_{\parallel}$ and $H_{\perp}$ represent the out-of-plane and in-plane magnetic field, respectively.

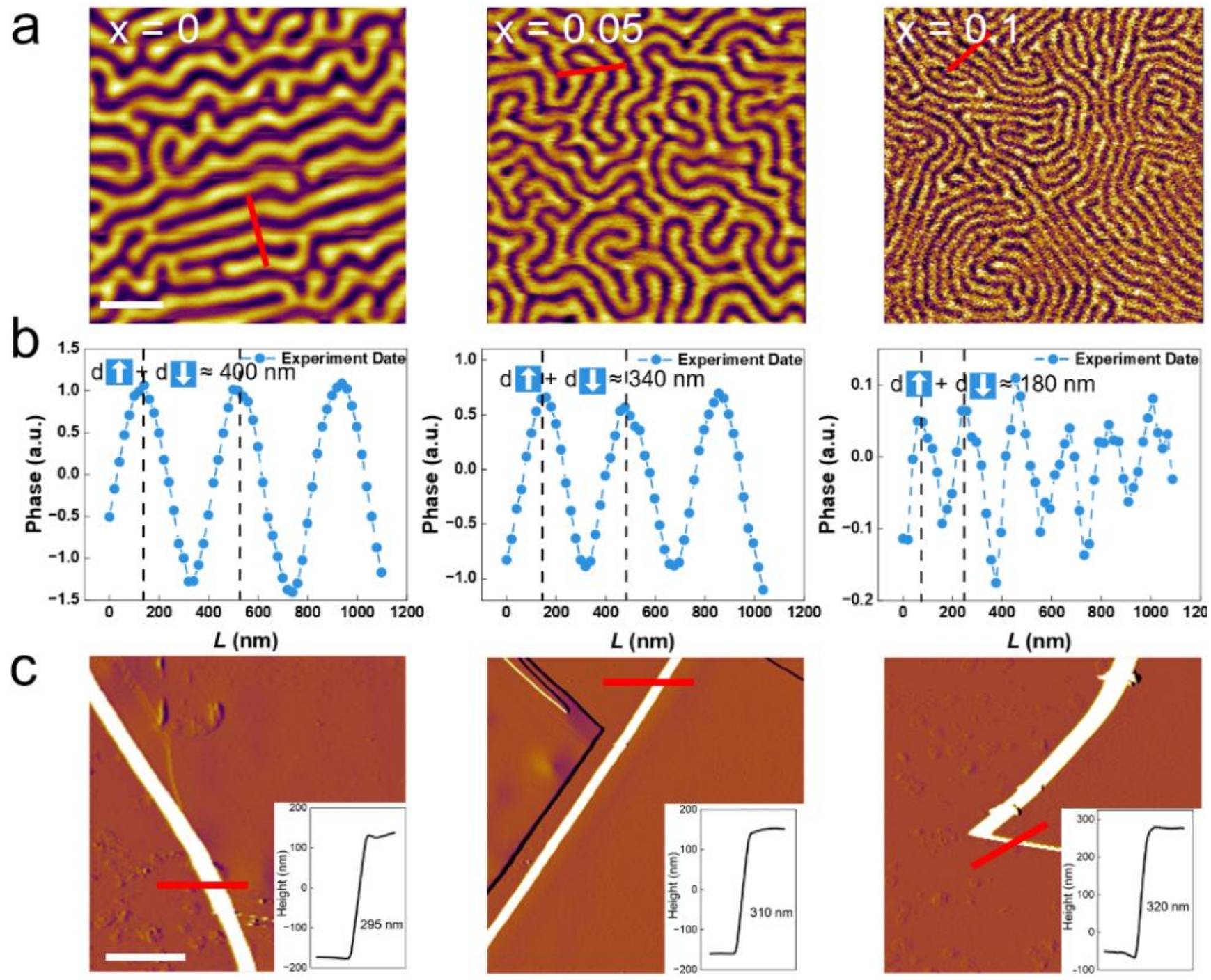

Figure S17. Establishing domain period (*w*) and domain wall energy (*σ*). a, Stripe domains of $Fe_{3-\delta}GaTe_2Pd_x$ with x = 0, 0.05 and 0.1 measured at room temperature and zero out-of-plane magnetic field using Magnetic Force Microscope (MFM). Scale bar is 1 um. **b,** Contrast variation extracted from the regions marked by red lines in MFM images. The distance between two neighboring contrast peaks is proposed to be a domain period (*w*). *w* for x = 0, 0.05, and 0.1 is 400 nm, 340 nm, and 180 nm, respectively.

The domain wall energy (σ) is calculated using the domain spacing model:

$$\frac{\sigma}{\mu_0 M_s^2 h} = \frac{w^2}{h^2} \sum_{\mathrm{odd}\ n=1}^{\infty} \left(\frac{1}{(\pi n)^3}\right) [1-(1-2\pi nh/w)\exp(-2\pi nh/w)], \qquad (14)$$

where h is the thickness of the film, and the thickness of the samples was 295 nm, 310 nm, and 320 nm for x = 0, 0.05, 0.1, respectively, as established by atomic force microscope in **c** (Scale bar in c is 5 um); M_s is the saturated magnetization and corresponds to 2.55×10^5 A/m, 1.9×10^5 A/m, and 1.66×10^5 A/m for x = 0, 0.05, 0.1, respectively (Supplementary Fig. S16). The corresponding value of σ is 1.6 mJ/m^3, 0.58 mJ/m^3, and 0.1 mJ/m^3 for x = 0, 0.05, 0.1, respectively. Notably, we measured w based on samples thicker than 300 nm, ensuring that the values of K_{eff} and M_s, which were established from bulk samples, remain valid for the subsequent DMI constant (D) calculation (Supplementary Fig. S17).

Functionals	Structure	J_1 (meV)	J_2 (meV)	J_3 (meV)
GGA	$Fe_{2.88}GaTe_2$	140.019	2.135	8.123
	$Fe_{2.88}GaTe_2Pd_{0.05}$	132.148	1.534	5.860
GGA + U (U = 1 eV)	$Fe_{2.88}GaTe_2$	149.568	0.011	0.047
	$Fe_{2.88}GaTe_2Pd_{0.05}$	142.261	0.206	0.825
GGA + U (U = 2 eV)	$Fe_{2.88}GaTe_2$	48.937	32.344	2.939
	$Fe_{2.88}GaTe_2Pd_{0.05}$	43.878	32.111	2.555
GGA + U (U = 2.5 eV)	$Fe_{2.88}GaTe_2$	36.431	30.513	4.267
	$Fe_{2.88}GaTe_2Pd_{0.05}$	31.732	28.196	3.827
GGA + U (U = 3 eV)	$Fe_{2.88}GaTe_2$	Difficult to converge		
	$Fe_{2.88}GaTe_2Pd_{0.05}$			
LDA	$Fe_{2.88}GaTe_2$	139.105	2.415	7.969
	$Fe_{2.88}GaTe_2Pd_{0.05}$	88.476	1.991	6.472
LDA + U (U = 1 eV)	$Fe_{2.88}GaTe_2$	163.917	0.808	2.277
	$Fe_{2.88}GaTe_2Pd_{0.05}$	124.925	1.499	4.980
LDA + U (U = 2 eV)	$Fe_{2.88}GaTe_2$	136.590	0.051	0.213
	$Fe_{2.88}GaTe_2Pd_{0.05}$	92.181	5.265	4.706
LDA + U (U = 2.5 eV)	$Fe_{2.88}GaTe_2$	118.923	0.457	2.527
	$Fe_{2.88}GaTe_2Pd_{0.05}$	76.690	2.609	4.348

Table S5. Calculated values of J_1, J_2, and J_3 for $Fe_{2.88}GaTe_2$ and Pd-intercalated $Fe_{2.88}GaTe_2$ based on first-principle calculations using GGA, GGA + U, LDA, and LDA + U functionals, where U is the on-site Coulomb interaction. We find that the reduction of ferromagnetic exchange induced by Pd intercalation is robust for all these methods, confirming the reliability of our theoretical model. In contrast, introducing a U significantly reduces the magnitude of J, bringing the calculated values closer to the experimental estimates. In particular, when a moderate U = 2.5 eV is applied within the GGA model, J_1 decreases to 31.732 meV for Pd-intercalated $Fe_{2.88}GaTe_2$. Further increasing U up to 3.0 eV, the self-consistent calculations become difficult to converge. Therefore, GGA + U approach with U = 2.5 eV is adopted in subsequent calculations.

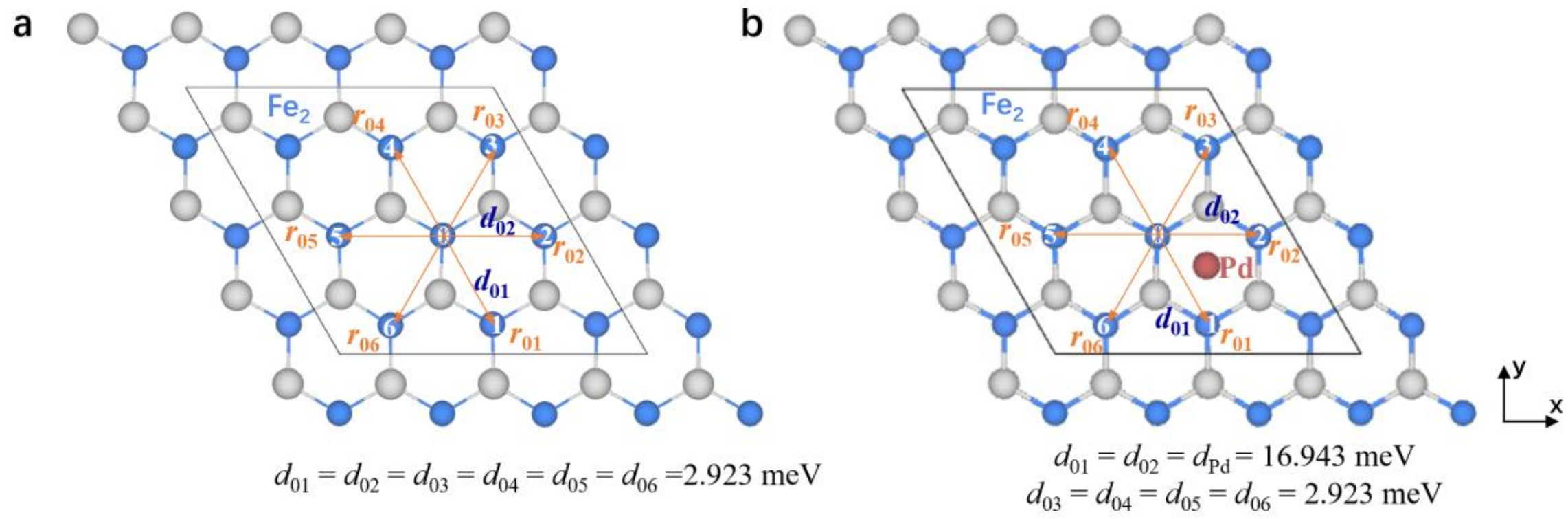

Figure S18. Schematic diagram of the DMI parameters d_{0j} and direction vector r_{0j} between different Fe_2-Fe_2 pairs in a $Fe_{2.88}GaTe_2$ and b $Fe_{2.88}GaTe_2Pd_{0.05}$. For the non-stoichiometric $Fe_{3-\delta}GaTe_2$ and $Fe_{3-\delta}GaTe_2Pd_x$, the Fe deficiency leads to a deviation of Fe_2 atoms towards *c*-axis direction, which breaks the inversion symmetry and results in the DMI [*Nat. Commun.* 15, 1017 (2024)]. Therefore, the DMI between the Fe_1 atom pairs and Te atoms are neglected, and that between the Fe_2 atom pairs and Te atom in $Fe_{2.88}GaTe_2$ and $Fe_{2.88}GaTe_2Pd_{0.05}$ is calculated based on a 3×3 supercell.

In our study, D is obtained via the equation [*Nat. Rev. Phys.* 5, 43 (2022)]:

$$D = \sqrt{D_x^2 + D_y^2 + D_z^2}\ , \qquad (15)$$

where D_x, D_y, D_z are the Cartesian components of D and are given by

$$D_{i\ (i = x,\ y,\ z)} = \frac{1}{V}\sum_j |(\boldsymbol{r}_{0j})_i| d_{0j}\ . \qquad (16)$$

Here, V represent the volume of the 3×3 supercell and is 2.01×10^{-27} m^3 for both $Fe_{2.88}GaTe_2$ and $Fe_{2.88}GaTe_2Pd_{0.05}$; $\boldsymbol{r}_{0j} = \boldsymbol{r}_j - \boldsymbol{r}_0$ is the direction vector from the reference Fe_2 atom at site 0 to its neighboring Fe_2 atom at site j (Fig. S18), and $(\boldsymbol{r}_{0j})_i$ (i = x, y, z) denotes the component of direction vector along x-, y-, z-axis, respectively; d_{0j} represents the DMI energy between reference Fe_2 atom at site 0 and the nearest-neighbor Fe_2 atoms at site j (Fig. S18). The magnitude of $\boldsymbol{r}_{0j}$ and their Cartesian components are listed in Table S6.

As shown in Table S6, the distances between nearest-neighbor Fe_2 pairs in $Fe_{2.88}GaTe_2$ are nearly the same. Therefore, for simplicity, we assume a uniform DMI energy $d_{0j} = d$ for all nearest-neighbor Fe_2 pairs in $Fe_{2.88}GaTe_2$. Based on the four-state method and GGA + U (U = 2.5 eV) model, d is calculated to be 2.923 meV [*Dalton Trans.* 42, 823 (2013); *Phys. Rev. B* 84, 224429 (2011)].

Atom	$\lvert\boldsymbol{r}_{0j}\rvert$ (Å)	$\lvert(\boldsymbol{r}_{0j})_x\rvert$ (Å)	$\lvert(\boldsymbol{r}_{0j})_y\rvert$ (Å)	$\lvert(\boldsymbol{r}_{0j})_z\rvert$ (Å)

$Fe_{2.88}GaTe_2$	1	3.99	1.96	3.47	0.04
	2	3.98	2.02	3.43	0.04
	3	3.99	1.97	3.47	0.03
	4	4.05	4.05	0.00	0.00
	5	4.05	2.02	3.51	0.00
	6	3.99	3.99	0.02	0.02
$Fe_{2.88}GaTe_2Pd_{0.05}$	1	3.98	1.96	3.47	0.07
	2	3.99	2.03	3.44	0.06
	3	4.00	1.96	3.48	0.04
	4	3.98	3.98	0.00	0.00
	5	4.05	2.03	3.51	0.00
	6	3.98	0.02	0.06	3.98

Table S6. Direction vectors $\boldsymbol{r}_{0j}$ and their components along the x-, y-, z-axis for $Fe_{2.88}GaTe_2$ and $Fe_{2.88}GaTe_2Pd_{0.05}$

For $Fe_{2.88}GaTe_2Pd_{0.05}$, the nearest-neighbor Fe_2-Fe_2 distances remain almost unchanged upon Pd intercalation. However, the presence of Pd provides additional spin-orbit coupling to nearby Fe_2 pairs, leading to an enhancement of the DMI energies. Accordingly, we assume that the DMI energy for Fe_2- Fe_2 pairs farther from the Pd atoms remian unchanged ($d_{03} = d_{04} = d_{05} = d_{06} = 2.923$ meV), while the DMI energy for Fe_2 pairs adjacent to the Pd atoms is enhanced, with $d_{01} = d_{02} = d_{Pd} = 16.943$ meV.

Based on equations (15) and (16), and using the DMI energies together with the corresponding direction vectors, the DMI constant D can are calculated to be 0.495 mJ/m^2 for $Fe_{2.88}GaTe_2$ and 1.368 mJ/m^2 for $Fe_{2.88}GaTe_2Pd_{0.05}$. These results demonstrate that Pd intercalation enhances the DMI, consistent with the experimentally extracted values. Detailed numerical results are summarized in Table S7.

	$Fe_{2.88}GaTe_2$	$Fe_{2.88}GaTe_2Pd_{0.05}$
d_{0j} (meV)	$d_{01} = d_{02} = d_{03} = d_{04}$ $= d_{05} = d_{06} = 2.923$	$d_{01} = d_{02} = d_{Pd} = 16.943$ $d_{03} = d_{04} = d_{05} = d_{06} = 2.923$
D_x (mJ/m^2)	0.374	0.818
D_y (mJ/m^2)	0.372	1.097
D_z (mJ/m^2)	0.003	0.002
D (mJ/m^2)	0.495	1.368

Table S7. Calculated total DMI constant *d* and *D* and the components of *D* along the x-, y-, z-axis in $Fe_{2.88}GaTe_2$ and $Fe_{2.88}GaTe_2Pd_{0.05}$.

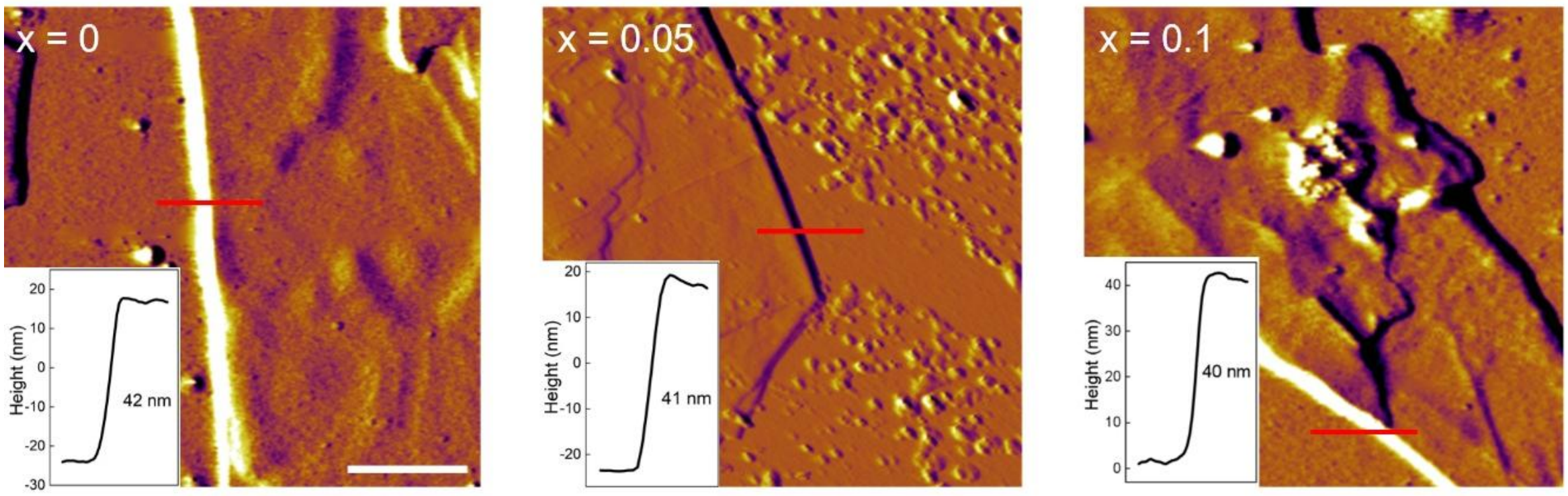

Figure S19. Atomic Force Microscopy (AFM) analysis of the sample morphology and thickness of $Fe_{3-\delta}GaTe_2Pd_x$ with x = 0, 0.05, and 0.1. Scale bar is 2 μm.

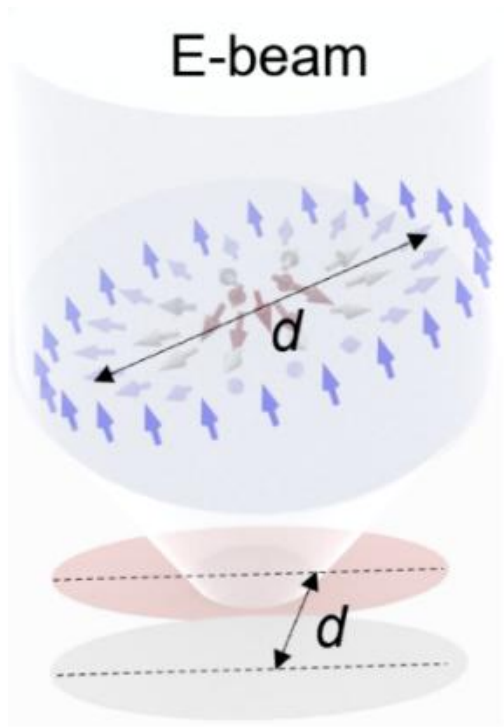

Figure S20. Schematic diagram of a titled Néel-type skyrmion as observed in LTEM. The skyrmion diameter (*d*) is defined as the distance between two in-plane spins that are oriented 180° (indicated by a black double-sided arrow). The corresponding skyrmion diameter observed in LTEM is represented by the distance between the dashed lines within the pink and gray shaded regions.

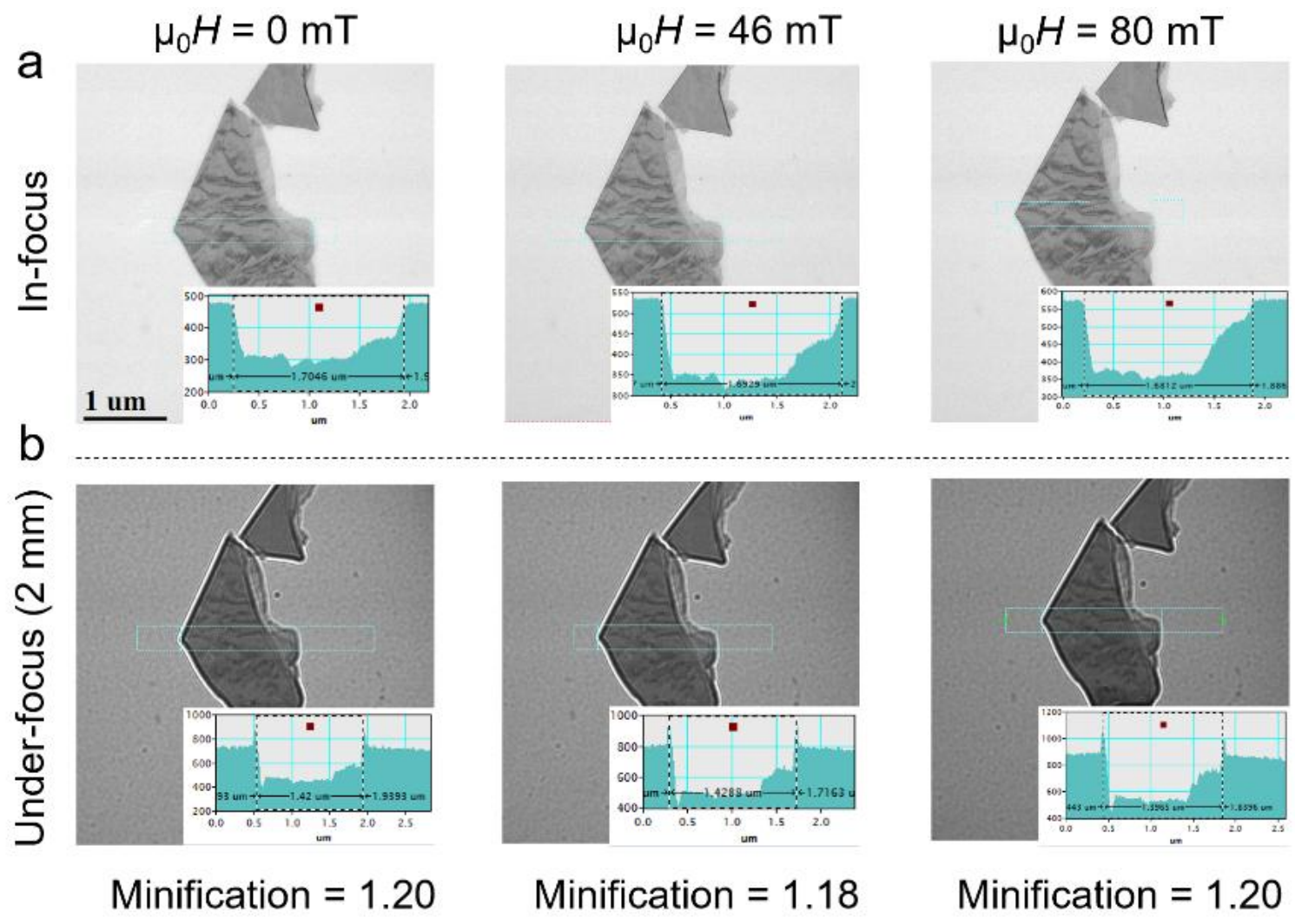

Figure S21. Correction of image size under varying magnetic fields in under-focused conditions. To quantify the impact of magnetic field application on image scaling, we used the in-focus image at $\mu_0 H$ = 0 mT as a reference (left panel of **a**). The application of a magnetic field exerts negligible influence on the size of in-focus images (middle and right panels of **a**). In contrast, images acquired at under-focused conditions exhibit noticeable size deviations (**b**). Specifically, a reduction in image size by approximately a factor of 1.2 was observed relative to the in-focus reference, highlighting the necessity of correcting for focus-induced distortions during image analysis.

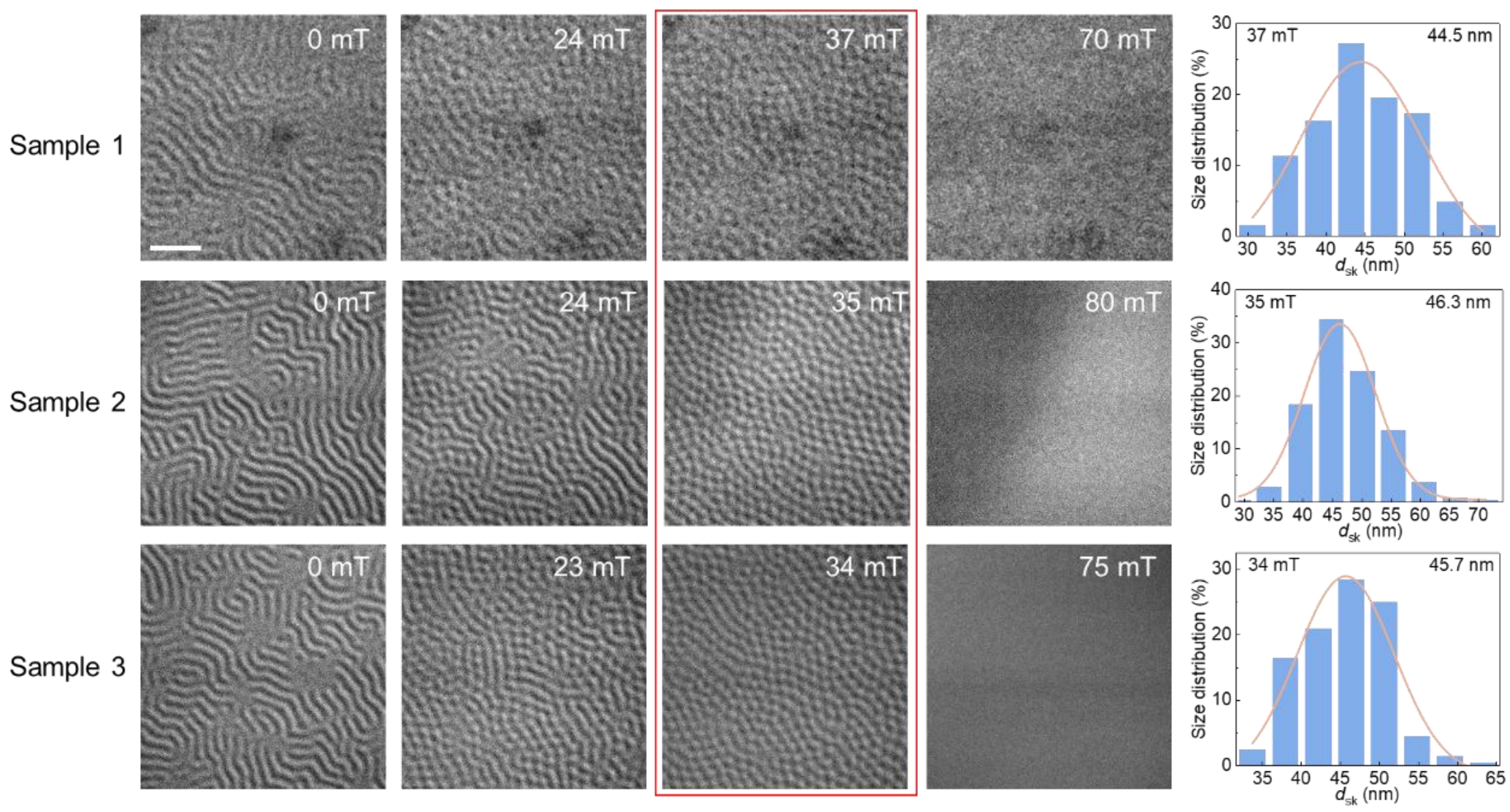

Figure S22. Magnetic-dependent domain evolution process. Evolution of magnetic domains with increasing magnetic field for three different $Fe_{3-\delta}GaTe_2Pd_{0.1}$ samples at room temperature. Size distribution of red marked skyrmions. The scale bar in each panel is 500 nm. The defocus value of the image is 2 mm.

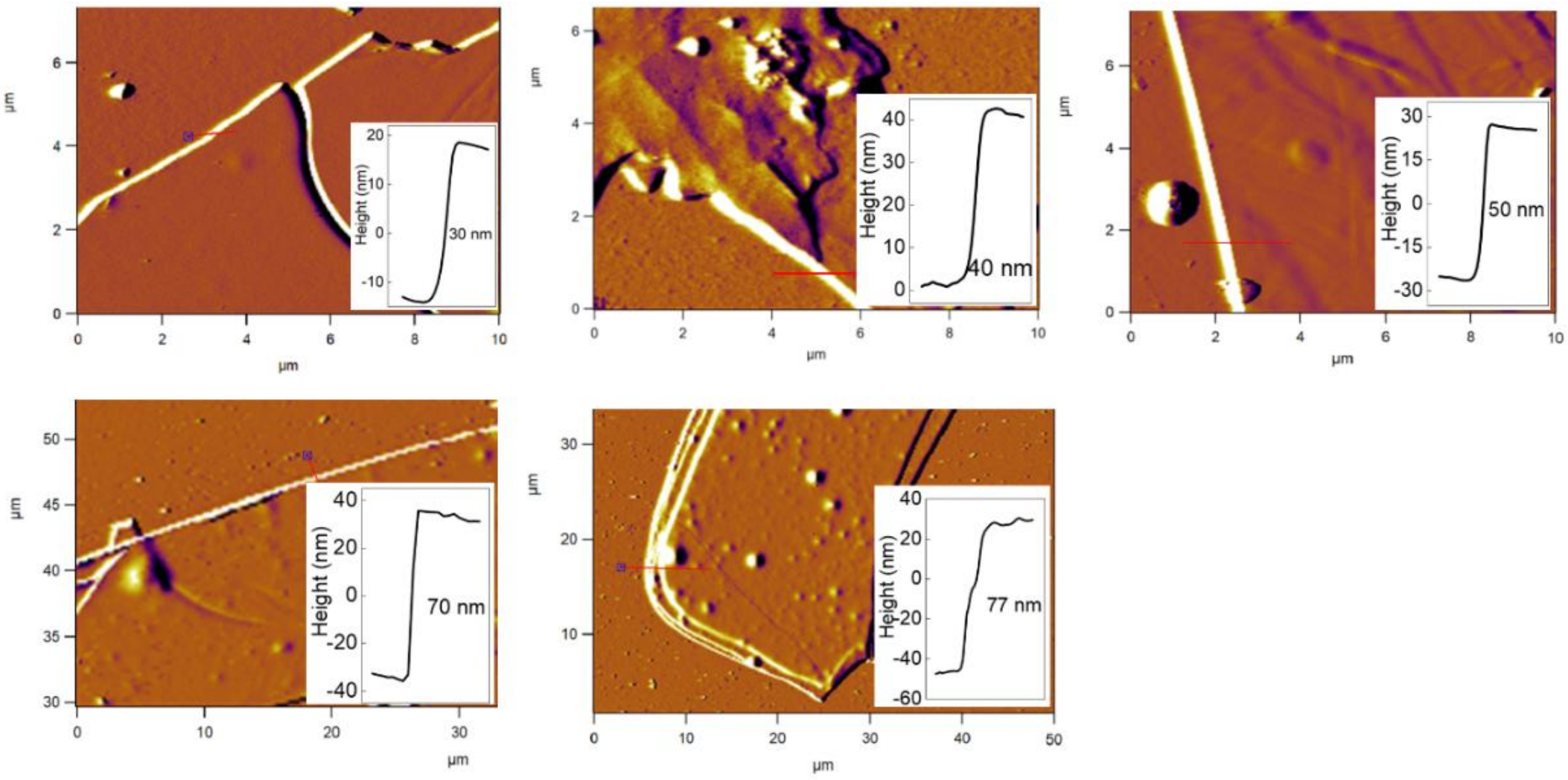

Figure S23. AFM image $Fe_{3-\delta}GaTe_2Pd_x$ for x = 0.1 with different thickness. Morphology and thickness measured using atomic force microscopy (AFM) for $Fe_{3-\delta}GaTe_2Pd_{0.1}$.

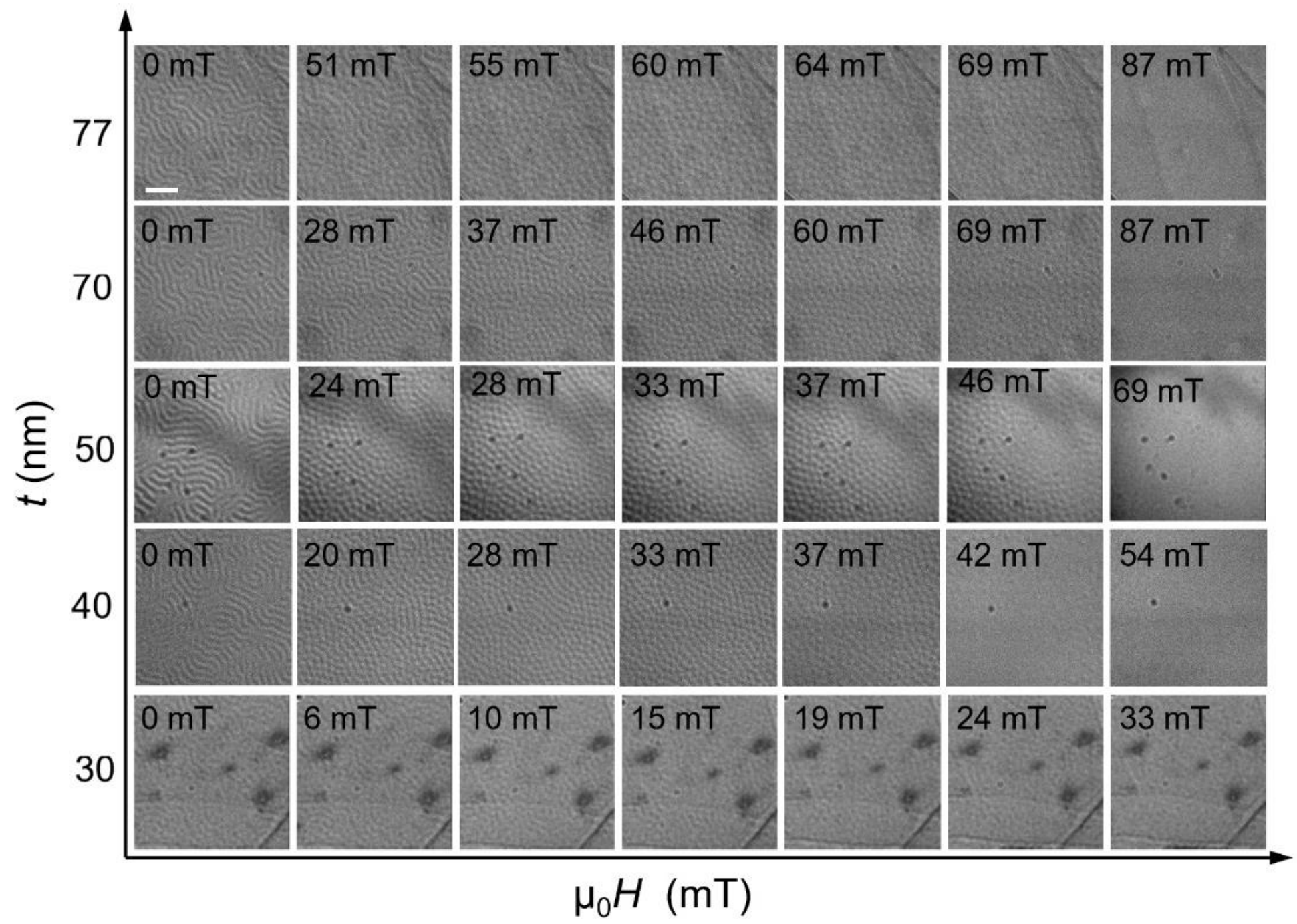

Figure S24. Evolution of magnetic domains under various thicknesses and magnetic field at $Fe_{3-\delta}GaTe_2Pd_x$ samples with x = 0.1. Scale bar is 500 nm. With decreasing thickness, the magnetic field for generation and annihilation of skyrmion decreases accordingly.

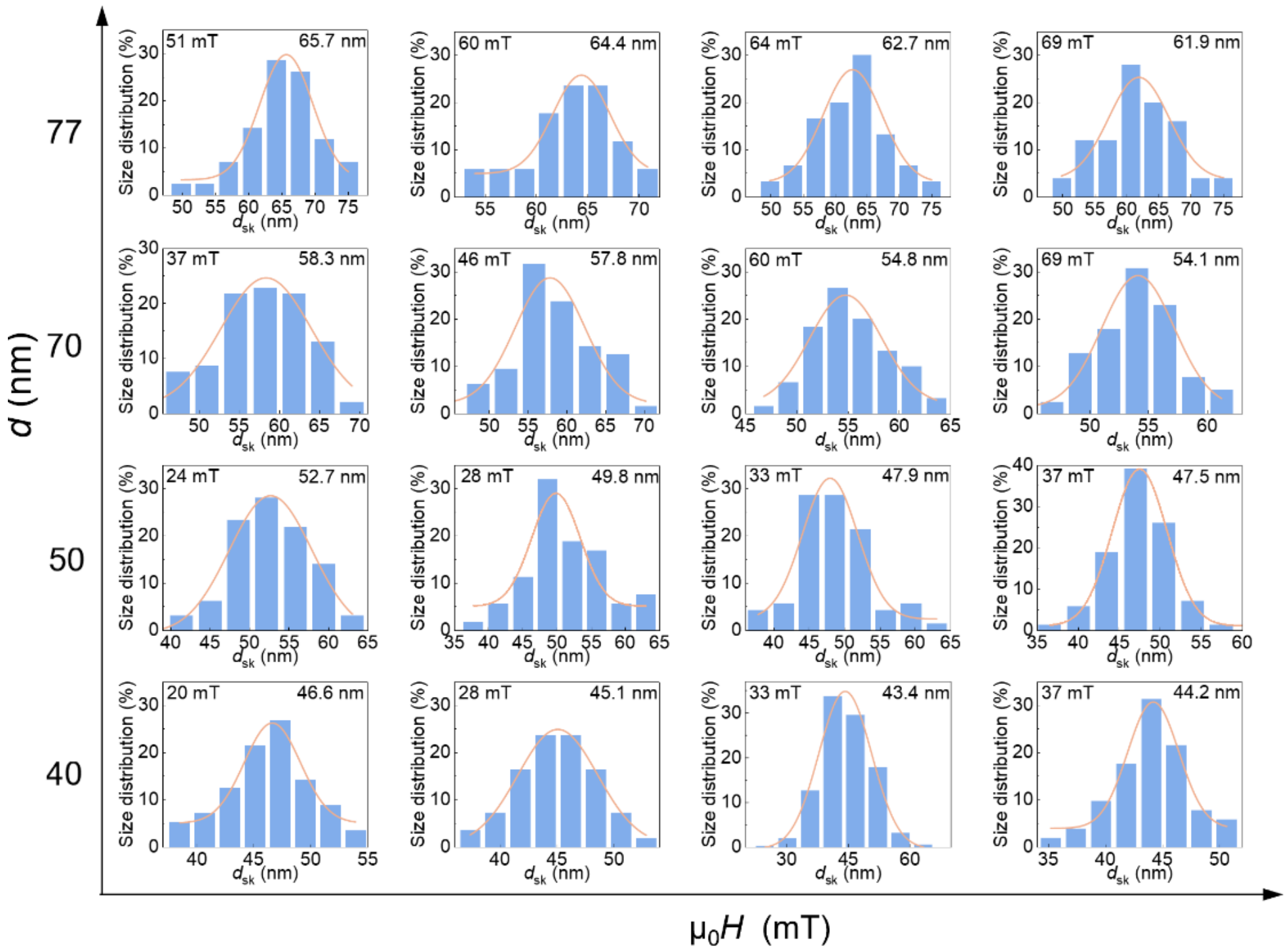

Figure S25. Size distribution of skyrmions under different magnetic fields ($\mu_0 H$) for x = 0.1 sample with different thickness (t). The skyrmion size is extracted from the LTEM images displayed in Figure S24.

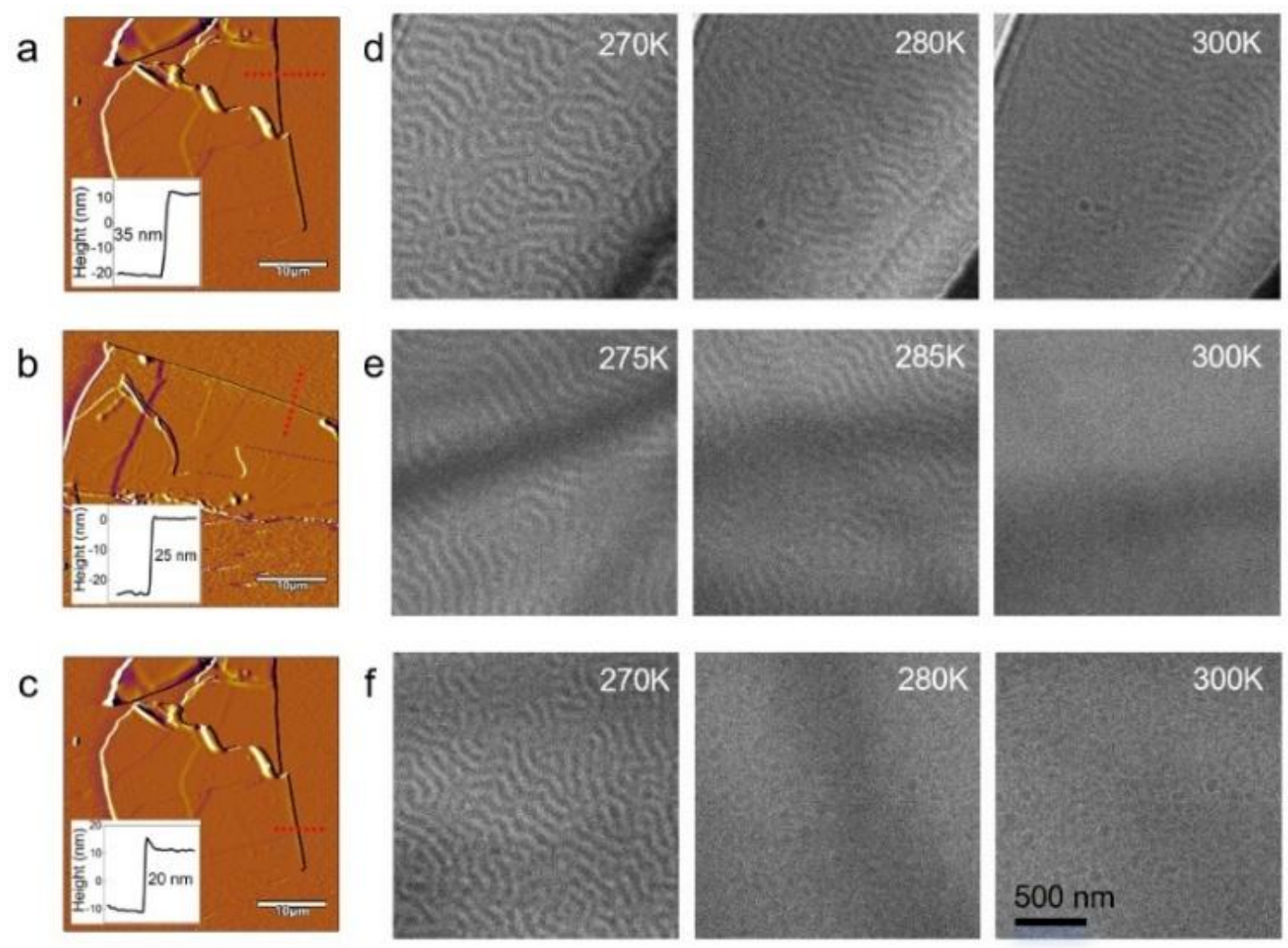

Figure S26. Evolution of magnetic domains under various thicknesses (*t*) and temperature (*T*) at $Fe_{3-\delta}GaTe_2Pd_{0.1}$. a-c, Atomic Force Microscopy (AFM) analysis of the sample morphology and thickness of $Fe_{3-\delta}GaTe_2Pd_{0.1}$. Scale bar is 10 μm. **d-f,** The corresponding evolution of magnetic domains with decreasing temperature for three distinct sample thicknesses. Scale bar is 500 nm. For vdW magnets, the Curie temperature (T_C) is known to decrease with reducing thickness, especially when the thickness falls below ~100 nm [*Nat. Commun*. 13, 5067 (2022); *Nano Lett.* 24, 993-1000 (2024)]. This behavior is

generally attributed to enhanced thermal spin fluctuations in reduced dimensions, which weaken the ferromagnetic exchange interactions. Previous studies have reported a thickness-induced reduction of T_C in Fe_3GaTe_2 flakes, decreasing from 380 K at a thickness of 112 nm to 360 K at 23 nm [*Nat. Commun*. 13, 5067 (2022)]. Therefore, a similar thickness dependence is expected in $Fe_{3-\delta}GaTe_2Pd_{0.1}$. For bulk $Fe_{3-\delta}GaTe_2Pd_{0.1}$, T_C is approximately 310 K. As the flake thickness decreases, T_C is expected to be reduced. Once T_C falls below room temperature, long-range ferromagnetic order is lost at 300 K and thus magnetic contrast is no longer visible in LTEM. To verify this scenario, we performed temperature-dependent LTEM experiments on $Fe_{3-\delta}GaTe_2Pd_{0.1}$ lamellae with t range of 35 nm-20 nm established using AFM analysis. For a t ~35 nm lamella, magnetic stripe domains are clearly visible at 300 K (Fig. S26d). In contrast, as t is reduced to 25 nm, no magnetic contrast is observed at 300 K. Upon cooling, magnetic stripe domains gradually emerge at 285 K (Fig. S26e). For a thinner flake (t ~20 nm), the appearance temperature of magnetic contrast shifts down to ~270 K (Fig. S26f). These results clearly confirm that the disappearance of magnetic contrast at room temperature for samples thinner than ~30 nm originates from the reduced T_C below room temperature.

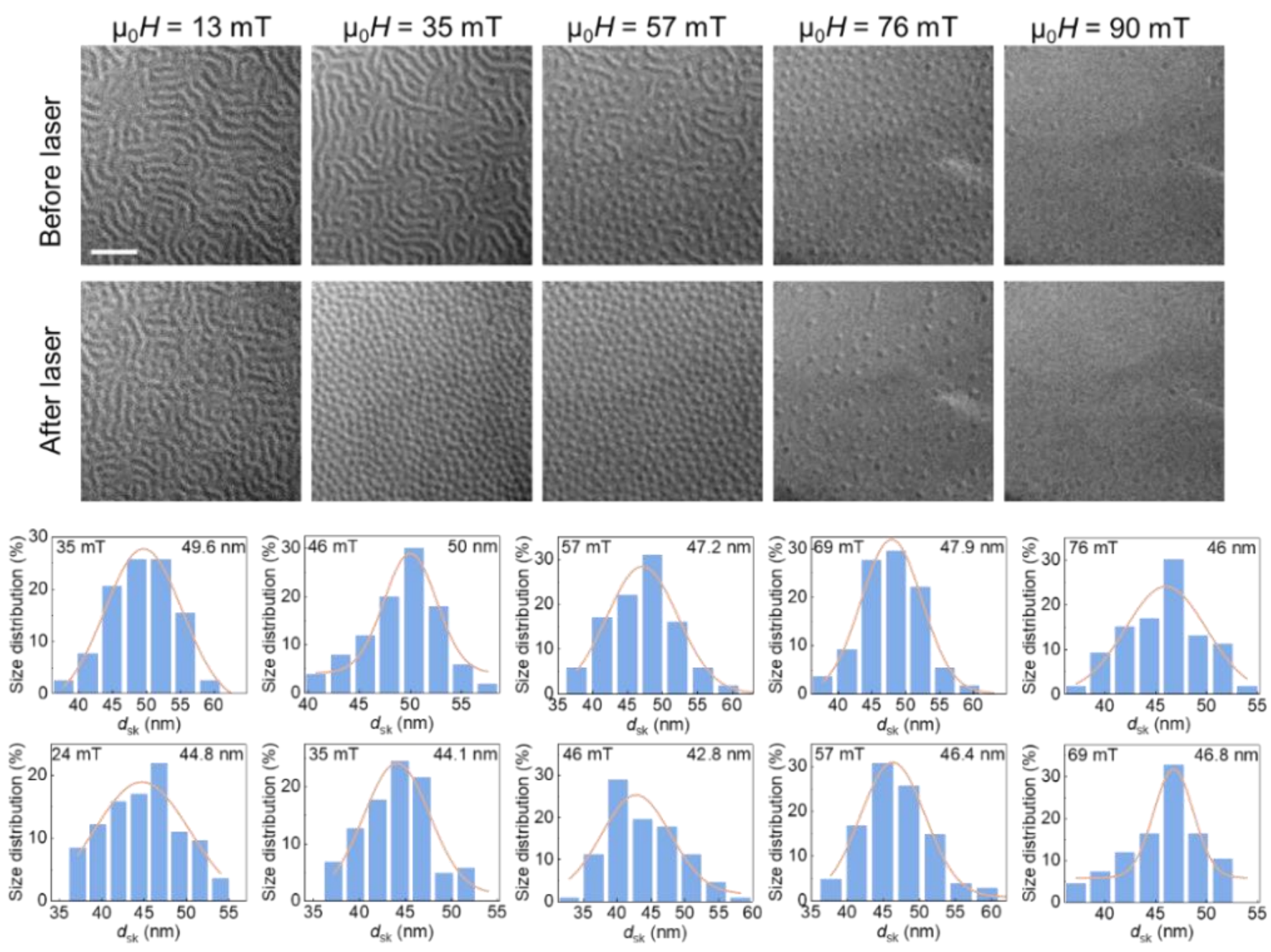

Figure S27. LTEM images taken before and after fs-laser pulse $Fe_{3-\delta}GaTe_2Pd_{0.5}$. The magnetic field ranges from 13 mT to 90 mT with a fluence of 6 mJ/cm². The scale bar is 500 nm. Insets show the size distribution of skyrmions.

Supplementary Note 3 for reduction of energy cost in Pd-intercalated $Fe_{3-\delta}GaTe_2$

In our study, the energy cost for per skyrmion is defined as:

$$E_{per} = E_{laser} / \rho, \qquad (17)$$

where E_{laser} is the total laser energy and ρ is the resulting skyrmion density. Accordingly, a lowing E_{per} can be achieved through a reduction of the laser energy (lower E_{laser}) and/or an increase in the skyrmion density (higher ρ). As described in the main text, femtosecond-laser-induced skyrmion generation operates through an ultrafast demagnetization process, where the laser pulse rapidly heats the sample to a temperature close to or above T_C, followed by skyrmion nucleation during the subsequent relaxation process. Because the required heating temperature is closely related to T_C, a reduced T_C naturally lowers the laser power required for skyrmion generation. In Pd-intercalated sample, the introduction of Pd induces a pronounced charge redistribution throughout the material system, which weakens the exchange interaction and suppresses T_C, thereby enabling skyrmion generation at reduced energy. In addition, Pd intercalation reshapes the magnetic interaction landscape by simultaneously reducing J and enhancing D. This leads to a smaller J/D ratio, favoring the stabilization of smaller skyrmions with a much higher density. Under femtosecond-laser excitation, the system experiences an ultrafast quenching process (>10^6-10^8 K/s), kinetically freezing the high-temperature magnetic texture into a metastable room-temperature configuration. Because the high-temperature phase in the Pd-intercalated sample corresponds to a lower J/D ratio than that of pristine $Fe_{3-\delta}GaTe_2$, the quenched state naturally hosts denser skyrmions, which in turn reduces the energy cost for manipulating an individual skyrmion.

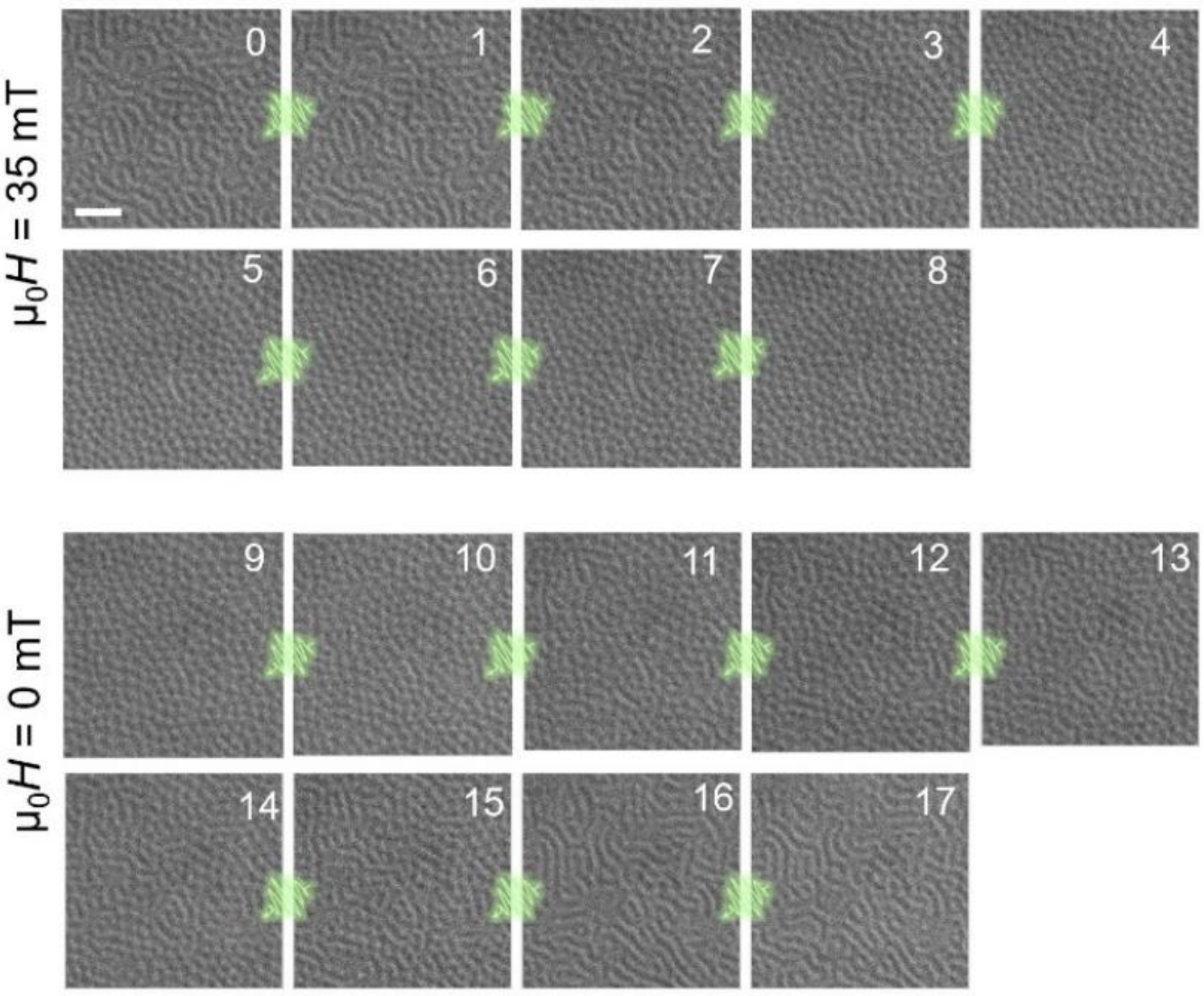

Figure S28. Laser induced nucleation and annihilation of skyrmions. Nucleation and annihilation of skyrmions under a series of laser pulses (520 nm wavelength, 50 µm focal spot size, and 240 fs pulse duration). The magnetic field for skyrmion nucleation and annihilation is 35 mT and 0 mT, respectively, with a fluence of 4.9 mJ/cm^2. The scale bar is 500 nm.

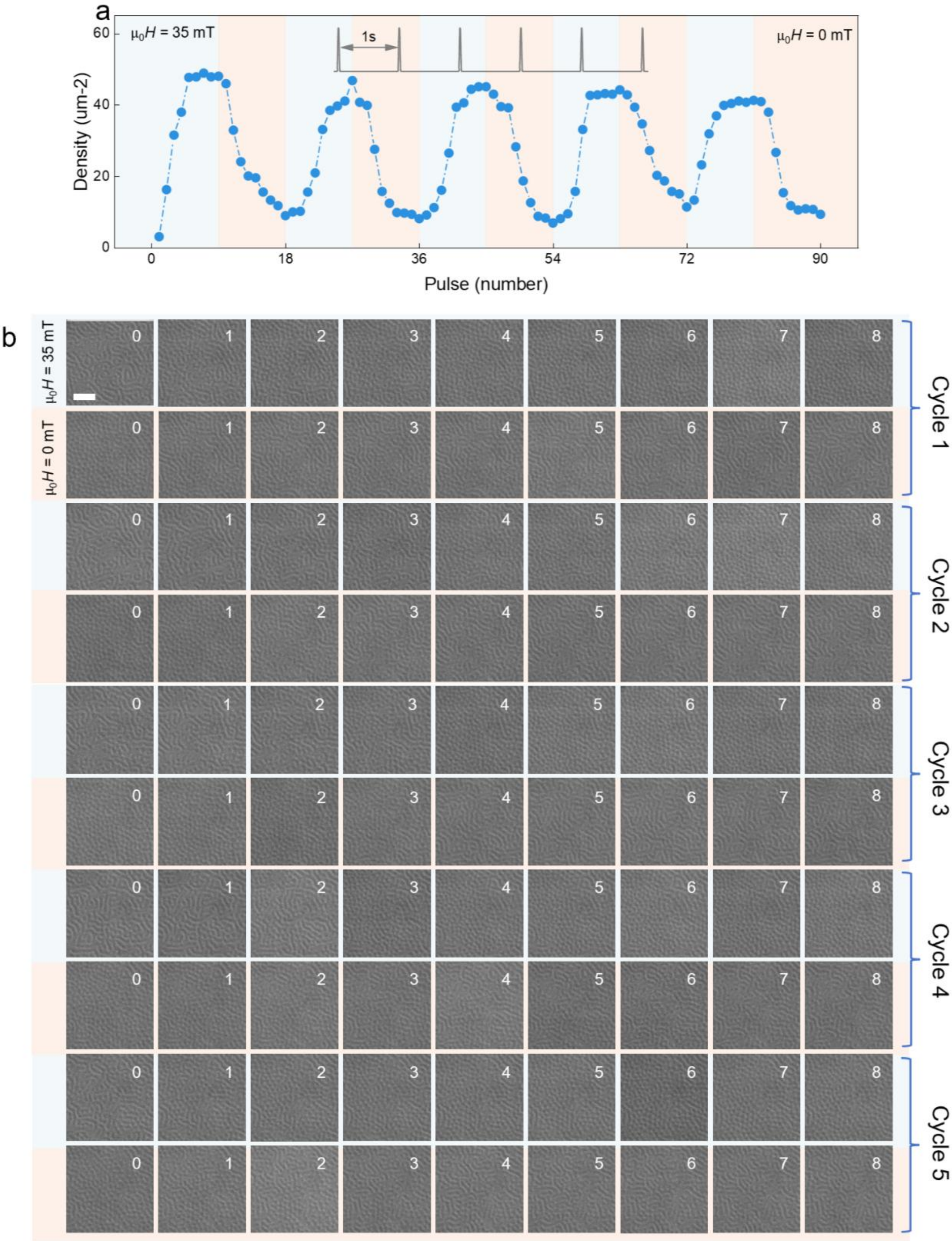

Figure S29. Nucleation and annihilation of skyrmions sample #2 under a series of ultra-fast laser pulses. The magnetic field is maintained at 35 mT and 0 mT, with a fluence of 5.2 mJ/cm^2. The scale bar is 500 nm.

Supplementary Note 4 for Neural network simulation

In this framework, the input image is represented as a pixel matrix with a dimension of 3@32×32, where 3 represents the number of image channels and 32×32 corresponds to the spatial resolution. This input matrix is progressively transformed into a feature matrix through a sequence of convolution (blue boxes) and max-pooling (red boxes) operations across layers 1-5. Taking convolutional layer 1 as an example, it comprises two consecutive convolution operations followed by a max pooling operation (Fig. S30). Firstly, the pixel matrix is convolved with the convolution kernels, each having a size of 3×3. During convolution, the kernel element is multiplied by the corresponding element in the 3×3 region of pixel matrix across all three image channels (marked by the red box in the pixel matrix), and the results are summed to produce a single output feature value (marked by the red box in the convolution result). The 3×3 region of pixel matrix then slides horizontally with a stride of 1 (marked by the blue box in the pixel matrix), and this process is repeated across all spatial positions, forming the first convolution layer. Given that there are 64 convolution kernels, the resulting convolution output has a dimension of 64. After two such convolution operations, a 2×2 max pooling operation with stride 2 is applied. In this process, the spatial resolution is reduced while retaining the maximum value, resulting in a feature matrix with dimension of 64@16×16.

The feature matrix is then processed by layers 2-5, with progressively increased channel depth and reduced spatial size. The resulting feature matrix dimensions are: 64@16×16 (layer 1), 128@8×8 (layer 2), 256@ 4×4 (layer 3), 512@2×2 (layer 4), and 512@1×1 (layer 5). The final 512@1×1 feature matrix is flattened into a one-dimensional array containing 512 elements, which is defined as the feature array X. This feature array is then fed into the fully connected layer to perform classification, producing the output probability vector Y with a dimension of 10.

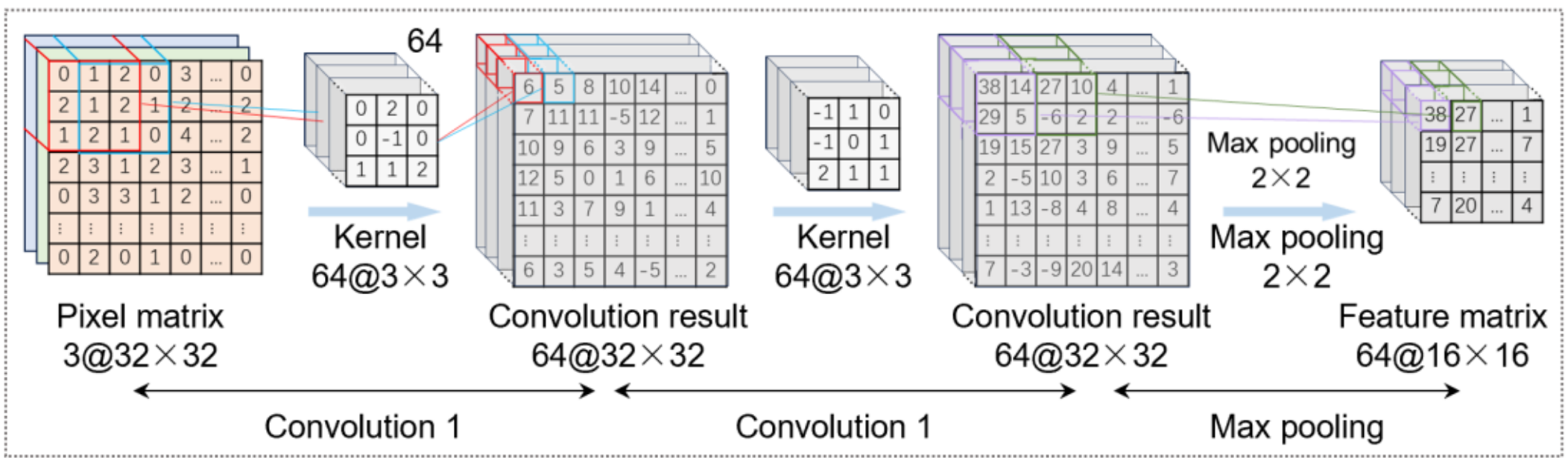

Figure S30. Detailed illustration of the convolution and max-pooling operations in convolutional layer 1. The input is a 32×32 pixel matrix with three RGB channels (red,

green and blue). After convolution with 64 kernels of size 3×3, 64 feature maps of size 32×32 (3@32×32) are generated. After two consecutive convolutions, a max pooling operation is applied, leading to 64 feature maps of size 16×16 (64@16×16). The 3@3×3 red boxes in the pixel matrix and 64@1×1 red boxes in convolution results indicate the data before and after the convolution operations, respectively. The 3@3×3 blue boxes in the pixel matrix and 64@1×1 blue boxes in convolution results represent the data before and after convolution after a horizontal shift of one step. In the max pooling process, the purple boxes highlight the data before and after the max pooling operation, where each 2×2 region is reduced to 1×1. The green boxes represent the pooling operation after two horizontal shifts.

The fully connected neural network in our work contains three layers, as schematically illustrated in Fig. S31. The feature array X is weighted and summed through three weight matrixes to obtain the output Y. For image classification, all features are weighted and summed to produce the output probability Y for each image category. The category with the highest probability is selected as the classification result. In this process, each weight factor w_{ij} in the weight matrix W represents the relationship between image features and categories, and is a multivalued parameter.

This parameter needs to be implemented by multistate physical states of the device in practical applications.

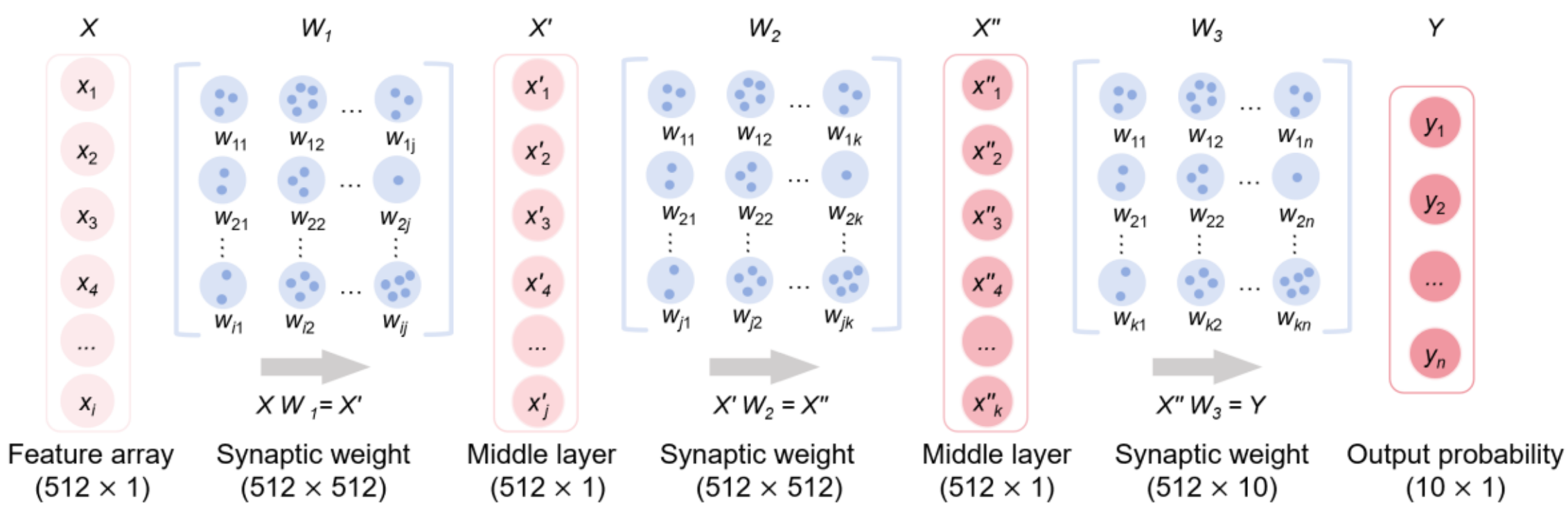

Figure S31. Schematic illustration of the fully connected layers. The input 512-dimensional feature array X is multiplied by the weight matrix W_1 to obtain the intermediate representation X', which is then multiplied by W_2 to produce X'', and finally multiplied by W_3 to generate the output Y. The numbers shown at the bottom of the figure indicate the data dimensions at each layer as well as the sizes of the corresponding weight matrices.
