# Supplementary material for: Ultrafast multi-level control of sub-50 nm skyrmions in a Pd-intercalated van der Waals magnet
Source: Nat Commun. 2026 Jul 21;17:8907. doi: 10.1038/s41467-026-75870-4 (PMC13503746; doi:10.1038/s41467-026-75870-4)
Supplement: Supplementary file 2 [file 41467_2026_75870_MOESM2_ESM.pdf]

nature portfolio

# Peer Review File

# Ultrafast Multi-level Control of Sub-50 nm Skyrmions in a Pd-Intercalated van der Waals Magnet

Corresponding Author: Professor Zhipeng Hou

Parts of this Peer Review File have been redacted as indicated to remove third-party material.

**This file contains all reviewer reports in order by version, followed by all author rebuttals in order by version.**

Version 0:

Reviewer comments:

Reviewer #1

(Remarks to the Author)
In this paper, the authors stabilize sub-50 nm skyrmions in van der Waals magnets by employing Pd intercalation. They then demonstrate the creation and annihilation of skyrmions using a femtosecond laser with ultra-low energy compared with previous reports. Finally, they discuss possible applications of their findings to neuromorphic computing, in which the creation and annihilation of skyrmions are utilized as the weights of a neural network.
Recently, applications of skyrmions have been intensively studied in spintronics and material science. Among them, skyrmion-based neuromorphic devices represent one of the most promising directions. Therefore, the demonstration of laser-based manipulation of skyrmions, which enables neuromorphic computing with ultra-low energy, is an important result in the field of spintronics and material science.
The manuscript is clearly written, and the quality of the data, analysis, and presentation are basically satisfactory. However, several ambiguous points remain, as itemized below. The authors should address these issues before publication.

1. In lines 307-309, the authors state that the magnetic contrast vanishes when the sample thickness is below 30 nm. What is the origin of this disappearance?　The origin does not seem to be explicitly described.

2. The authors demonstrate the manipulation of skyrmions using a femtosecond laser with much-lower energy than in previous reports. What is the fundamental factor that enables this ultra-low-energy manipulation? Is Pd intercalation directly responsible for achieving such ultra-low-energy manipulation?

3. In the demonstration of neuromorphic computing, the description of the detailed simulation procedure is completely lacking. The authors claim that the number of skyrmions encodes the synaptic weight. However, they do not explain how the experimental observations are connected to the neuromorphic computing simulations.

4. Minor comment: In the abstract, the authors state that skyrmion-based optical neuromorphic computing achieves a high accuracy of 92 %. However, this result is obtained by simulation. To avoid overstating the claim, it would be better to explicitly clarify that this is based on simulation.

Reviewer #2

(Remarks to the Author)
In this manuscript, the authors present LTEM studies on Pd-intercalated non-stoichiometric $Fe_{3-\delta}GaTe_2$. They report that Pd intercalation can tune the Dzyaloshinskii–Moriya interaction (DMI), resulting in the formation of smaller skyrmions compared to pristine $Fe_{3-\delta}GaTe_2$. In addition, they demonstrate the nucleation and manipulation of skyrmion density using femtosecond laser pulses, and show that the density can be tuned with pulse length, which they suggest could be relevant for neuromorphic computations.

While the study is carefully performed, in my view, the results are incremental in nature. A closely related work by one of the corresponding authors has already been published [Nat. Commun. 15, 1017 (2024)], and the present manuscript mainly reports a quantitative reduction in skyrmion size and an increase in density upon Pd intercalation. The novelty, therefore, appears limited, as no fundamentally new insights into the skyrmion physics of $Fe_{3-\delta}GaTe_2$ are presented. Moreover, although Pd intercalation is emphasized, the role of self-intercalation is not addressed, making it difficult to disentangle the specific effect of Pd from possible intrinsic effects.

Considering the above points, I do not believe the work meets the criteria for publication in Nature Communications. However, the manuscript may be more suitable for a specialized journal that publishes incremental advances in the field of 2D magnetic materials and skyrmion physics.

Reviewer #3

(Remarks to the Author)
Through combining different theoretical and experimental methods, such as micromagnetic simulations, first-principal calculations, LTEM Measurements, the authors explored the ultrafast and multi-level control of nanoscale skyrmions in Pd-Intercalated vdW magnet Fe3-δGa2Te2. They realized sub-50 nm magnetic skyrmions at room temperature with the help of magnetic field. They deemed that the enhanced DM interactions and suprressed Heisenberg exchange couplings are responsible for the size reduction of magnetic skyrmsions in Pd-Intercalated Fe3-δGa2Te2. Besides, they generate magnetic skyrmions and modulate magnetic skyrmion density using femtosecond laser. This work provides insights for understanding the magnetic skyrmions in vdW magents, and several issues are needed to be make clear.

1) The micromagnetic simulation in MuMax is based on the LLG equation, so what is the damping parameter for the LLG equation in simulations? Does the damping paramter have any effect on the simulation results?
2) It looks that the Heisenberg exchange J, DM interactions D in Figure 2d are obtanied by experiments. In Supplementary Table 3 and 4, the Heisenberg exchange constant (J) and DM interaction (D) for both Fe2.88GaTe2Pd0.05 and Fe2.88GaTe2 are given. are the experimental and theoretical resutls consistent?
3) The calculated J1 of both Fe2.88GaTe2Pd0.05 and Fe2.88GaTe2 in Supplementary Table 3 are so large that I suggest the author should make a double check on this.
4)The authors used only J1, J2 and J3 for Fe2.88GaTe2Pd0.05 and Fe2.88GaTe2. This should be not reanable because Fe2.88GaTe2Pd0.05 and Fe2.88GaTe2 has a relative low symmetry. Such rough classification of Heisenberg exchanges may cause un-reasonable calculation results.
5) The calculated DM interactions given in figure 2d and Supplementary Table 4 should use the same units, so it is easy for reader to compare the experimental and thoretical results. Explicitly, are the experimental and thoretical result of DM interactioins are consistent?
6) What are the calculated magnetic moments of Fe atoms in Fe3GaTe2? Are they consistent with experiental results? As far as I know, the GGA functional in DFT calculations will overestimate the magnetic moments of Fe atoms obvioulsy, so LDA potential should be more reasonable used to study the magnetic properites of Fe3GaTe2.
7) The considered Fe vacanies are distributed in two different Fe3GaTe2 layers. In principles, Fe vacanies can be distributed in the same layer in practice. So does the favorable Pd doping site depend on the Fe vacancies configurations in theoretical calculations and experimental samples?
8) The theoretical results play an important to obtain the experimental observations in this work, so some important details for micromagnetic simulations and first-principal calculations should be given in the “Methods” part in the maintext.
9) “first-principal” should be “first-principle”.

Version 1:

Reviewer comments:

Reviewer #1

(Remarks to the Author)
I appreciate the authors’ sincere response to my comments. While most concerns have been addressed, I find that the reply to Comment 3 is still insufficient and may contain inaccuracies.

In the revised manuscript, the explanation of the connection between experiment and simulation has become clearer, in which a new quantity “feature array X” is introduced. However, at least in the present explanation, it remains unclear how this feature array is obtained. I assume that the authors use the VGG network provided in Fig. 5c of the initial manuscript (although its structure has been removed in the revised version). However, according to Fig. 5c in the revised manuscript, the dimension of the feature matrix is n and is the same as that of the output probability. If my understanding is correct, n = 10 in this case, which differs from the dimension of the final feature layer of the VGG network (layer 5), namely 512. This point is confusing, and I recommend providing a more detailed explanation and/or correcting possible mistakes.

In addition, in any case, weights are required to obtain the feature array X. From the revised explanation of the simulation, it appears that the skyrmion device is used only in the final layer, namely in the calculation of Y from X. Thus, it is unclear what advantage is gained by using the skyrmion device only in the final layer. For example, the VGG network contains more than one million parameters. Thus, if the authors use the VGG network to calculate X, the number of parameters in the final layer

is much smaller compared with the total number of parameters. To emphasize the high accuracy of the skyrmion device, should the authors perform simulations in which the skyrmion device is used for all layers?

Before publication, these points should be clarified.

Reviewer #2

(Remarks to the Author)
I remain unconvinced by the authors' rebuttal that the present work meets the novelty threshold required for publication in Nature Communications.
The core outcome of this manuscript remains an incremental advance rather than a fundamentally new physical insight. As already noted in my earlier report, a closely related study by one of the corresponding authors has recently been published in Nature Communications [Nat. Commun. 15, 1017 (2024)]. The current manuscript primarily reports a reduction in skyrmion size and an increase in skyrmion density upon Pd intercalation, which—given the existing literature—are anticipated consequences of modifying exchange interactions and DMI via heavy-element intercalation.
The authors emphasize "vdW gap engineering" as a key novelty. While I agree that intercalation engineering is an important approach, this concept is already well established in Fe-based vdW magnets. For example, transition-metal intercalation (such as Co in $Fe_5GeTe_2$) and self-intercalation effects are widely known to alter symmetry, magnetic anisotropy, and DMI. In $Fe_{3-\delta}GaTe_2$ specifically, symmetry breaking and the stabilization of Néel-type skyrmions are fundamentally linked to Fe deficiency and Fe self-intercalation in the vdW gaps. Without such intercalation, the parent compound should not host Néel-type skyrmions.
In this context, the authors' strong claim—based on STEM/EELS—that no Fe exists in the vdW gaps and that only Pd is present is difficult to accept unambiguously. Given the presence of heavy Pd atoms, the absence of detectable Fe signal in STEM/EELS does not conclusively rule out Fe self-intercalation, as Fe contrast can be strongly suppressed. Therefore, it remains challenging to disentangle whether the observed modification of DMI arises exclusively from Pd intercalation, or whether intrinsic self-intercalation effects also contribute. As a result, the assertion that Pd intercalation alone creates a "new material platform" is not sufficiently justified. The observed reduction in skyrmion size and the enhanced density—particularly under varying laser fluence are expected trends within the established framework of skyrmion physics, where heavy elements are routinely employed to tune interfacial or bulk DMI in thin films and layered magnets.
Consequently, while the work is thorough and may be suitable for a specialized journal focused on advances in 2D magnetic materials or skyrmion physics, I do not believe it demonstrates the level of conceptual novelty or fundamental advance required for publication in Nature Communications.

Reviewer #3

(Remarks to the Author)
The authors addressed my concerns, so this work can be published in Nature Communications.

Version 2:

Reviewer comments:

Reviewer #1

(Remarks to the Author)
I appreciate the authors' sincere response to my comments.

In the previous review, I raised two concerns: (1) an insufficient/incorrect explanation of the neural network simulation and (2) the use of skyrmion devices only in a part of the network.

Regarding comment #1, the revised manuscript explains the neural network simulation procedure well and meets the required standard.

However, regarding comment #2, I think that the authors' response is still insufficient to demonstrate that the impact of this paper is sufficient for publication in Nature Communications. While the authors mention that the fully connected layers play a more critical role in the network, the convolutional layers are also essential for the CIFAR-10 task and therefore also play a critical role; without convolutional layers, the CIFAR-10 task cannot be solved. Therefore, the reason why the fully connected layers are treated specially (i.e., why the skyrmion device is used only in the fully connected layers) is still unclear. For example, if both convolutional and fully connected layers are implemented using a conventional architecture, and if the energy consumption of the fully connected layers is much larger than that of the convolutional layers, then the proposed hardware architecture would be meaningful. However, without such a discussion, I cannot evaluate the importance or significance of the proposed "hybrid" skyrmion-based optical neuromorphic computing device.

In addition, in the abstract, the authors state "skyrmion-based optical neuromorphic computing with a simulated training accuracy of ~92%". However, as described above, since this accuracy is obtained from a simulation of a "hybrid" hardware system (with convolutional layers implemented by a conventional architecture and fully connected layers by a skyrmion

device), this description may be misleading.

In summary, I find that the advantage or significance of the high CIFAR-10 accuracy (92 %) achieved by the present skyrmion-based optical neuromorphic computing system is not sufficiently clear. Given that this high accuracy is one of the main results of the study, as highlighted in the abstract, the authors should provide a more rigorous justification before publication.

Reviewer #2

(Remarks to the Author)
The authors have addressed the concerns raised in the review carefully. However, from the manuscript, it has become clear that the synthesis of this material is quite specific and non-trivial. In the literature, some reports describe Fe-enriched $Fe_{3+x}GaTe_2$ compounds, whereas the present work appears to involve Fe-deficient compositions with Pd intercalation. Because of this difference, it is important that the synthesis procedure be described in much greater detail before publication.

In particular, the authors should provide a detailed description of the synthesis of the Pd-intercalated compounds in the Supporting Information. The current description of the synthesis is very general, and it is likely that certain specific steps or experimental strategies were necessary to successfully obtain these materials. The authors mention that they attempted to substitute or dope other elements but were unsuccessful; this further suggests that the synthesis conditions are quite sensitive and therefore should be clearly documented.

Although EDX data are provided, the authors have not included XRD results for the Pd-intercalated compounds. Structural characterization, such as XRD, should be included to confirm phase formation. Additionally, the authors should clarify how Ga was retained in the sample during synthesis and whether any specific strategy was employed to successfully achieve Pd intercalation.

The authors also show DFT calculations indicating that the Pd-intercalated structure is energetically more stable than the non-intercalated compound. However, it would be helpful if the authors could provide further discussion on the underlying chemistry that allows Pd to intercalate successfully. Why does Pd work in this structure, while other attempted substituents do not? Clarifying the chemical or electronic factors that determine the successful incorporation of Pd would significantly strengthen the manuscript.

Providing these experimental and conceptual details is important to ensure that the synthesis can be reproduced by other researchers in the future. Once these points are addressed and the synthesis procedure is described in sufficient detail in the Supporting Information, I believe the manuscript would be suitable for publication.

Version 3:

Reviewer comments:

Reviewer #1

(Remarks to the Author)
I appreciate the authors' sincere response to my comments. In the revised manuscript, the authors have addressed all of my concerns by performing an additional simulation. Thus, I recommend publication of the manuscript.

Reviewer #2

(Remarks to the Author)
The explanations are satisfactory overall, and the manuscript can be accepted after minor clarification.

However, regarding the synthesis procedure, there appears to be a possible inconsistency in the authors' responses. In response to Comment 1, the authors mentioned that the liquid flux was removed by centrifugation. Later, in response to Comment 3, they stated that flakes must be carefully selected because secondary phases such as GaTe and FeTe are also formed along with Fe3GaTe2. These statements appear somewhat contradictory.

The authors also mentioned that the crystallinity of the synthesized material is lower than that of the pristine parent compound, possibly due to the introduction of grain boundaries within the planes. Based on this explanation, it may also be inferred that the presence of such grain boundaries could hinder the growth of large single crystals. If so, this synthesis approach may face limitations in scalability for obtaining sizable, high-quality single crystals.

Therefore, it would be helpful if the authors could clarify:

how effectively the molten flux is removed after centrifugation,
whether residual flux contributes to the formation of GaTe and FeTe secondary phases,
the approximate fraction or extent of these impurity phases,
and whether the reduced crystallinity and grain-boundary formation limit the growth of large single crystals.

It would also be useful if the authors could mention the typical crystal dimensions obtained in the synthesis. A small inset optical image of representative crystals placed on graph paper or with a scale bar would significantly help readers assess the crystal size and quality. Including these clarifications in the Supplementary Information would strengthen the manuscript further.

Version 4:

Reviewer comments:

Reviewer #2

(Remarks to the Author)
The authors have satisfactorily addressed all the concerns raised during the rebuttal process. I am convinced by their responses and the revisions made to the manuscript. Therefore, I recommend the manuscript for publication.

**Open Access** This Peer Review File is licensed under a Creative Commons Attribution 4.0 International License, which permits use, sharing, adaptation, distribution and reproduction in any medium or format, as long as you give appropriate credit to the original author(s) and the source, provide a link to the Creative Commons license, and indicate if changes were made.
In cases where reviewers are anonymous, credit should be given to 'Anonymous Referee' and the source.
The images or other third party material in this Peer Review File are included in the article's Creative Commons license, unless indicated otherwise in a credit line to the material. If material is not included in the article's Creative Commons license and your intended use is not permitted by statutory regulation or exceeds the permitted use, you will need to obtain permission directly from the copyright holder.
To view a copy of this license, visit https://creativecommons.org/licenses/by/4.0/

**Response to the Report of Reviewer A**

**Reviewer A's Comment:** In this paper, the authors stabilize sub-50 nm skyrmions in van der Waals magnets by employing Pd intercalation. They then demonstrate the creation and annihilation of skyrmions using a femtosecond laser with ultra-low energy compared with previous reports. Finally, they discuss possible applications of their findings to neuromorphic computing, in which the creation and annihilation of skyrmions are utilized as the weights of a neural network. Recently, applications of skyrmions have been intensively studied in spintronics and material science. Among them, skyrmion-based neuromorphic devices represent one of the most promising directions. Therefore, the demonstration of laser-based manipulation of skyrmions, which enables neuromorphic computing with ultra-low energy, is an important result in the field of spintronics and material science.

The manuscript is clearly written, and the quality of the data, analysis, and presentation are basically satisfactory. However, several ambiguous points remain, as itemized below. The authors should address these issues before publication.

**Authors' Reply:** We sincerely thank the reviewer for careful reading of our manuscript and for recognizing the significance of laser-based manipulation of skyrmions for neuromorphic computing applications. We also appreciate the reviewer's insightful comments, which are of great importance to improve the quality of our manuscript. Below, we address each comment in a point-by-point manner. We hope the reviewer will be satisfied with the revised manuscript as well as our responses.

**Comment #1:** In lines 307-309, the authors state that the magnetic contrast vanishes when the sample thickness is below 30 nm. What is the origin of this disappearance? The origin does not seem to be explicitly described.

**Authors' Reply:** We highly appreciate the reviewer's valuable comments, which are of great significance to improve the quality of our manuscript. In van der Waals (vdW) magnets, the Curie temperature ($T_C$) is known to decrease with reducing thickness, particularly when the thickness falls below ~100 nm [*Nat. Commun*. **13**, 5067 (2022); *Nano Lett.* **24**, 993 (2024)]. This feature is generally attributed to enhanced thermal spin fluctuations in reduced dimensions, which weaken the ferromagnetic exchange interactions. Consistent with this trend, previous studies have reported a reduction of $T_C$ in $Fe_3GaTe_2$ flakes from 380 K at a thickness of 112 nm to 360 K at 23 nm [*Nat. Commun*. **13**, 5067 (2022)]. Based on these observations, a similar thickness-dependent

evolution of $T_C$ is therefore expected in $Fe_{3-\delta}GaTe_2Pd_{0.1}$.

For bulk $Fe_{3-\delta}GaTe_2Pd_{0.1}$, $T_C$ is approximately 310 K (**Fig. 2c** in the main text). As the flake thickness decreases, $T_C$ is expected to be reduced. Once $T_C$ falls below room temperature, long-range ferromagnetic order is lost at 300 K and thus magnetic contrast is no longer visible in Lorentz transmission electron microscopy (LTEM). To verify this scenario, we performed temperature-dependent LTEM experiments on $Fe_{3-\delta}GaTe_2Pd_{0.1}$ lamellae with a thickness (*t*) ranging from 35 nm to 20 nm, as determined by atomic force microscopy (AFM) (**Figs. R1a-c**). For a *t* ~35 nm lamella, magnetic stripe domains are visible at 300 K (**Fig. R1d**). In contrast, as *t* is reduced to 25 nm, no magnetic contrast is observed. Upon cooling, magnetic stripe domains gradually emerge at 285 K (**Fig. R1e**). For a thinner flake (*t* ~20 nm), the appearance temperature of magnetic contrast shifts down to ~270 K (**Fig. R1f**). These results clearly confirm that the disappearance of magnetic contrast at room temperature in samples thinner than ~30 nm originates from a thickness-induced reduction of $T_C$ below room temperature.

In the revised manuscript, the discussions on the origin of the magnetic contrast disappearance have been added into the main text (Page 11, Lines 316-317) and Supplementary Information (revised Supplementary **Fig. S24**). Related references are also cited in the main text (see References 53, 63 in the revised main text).

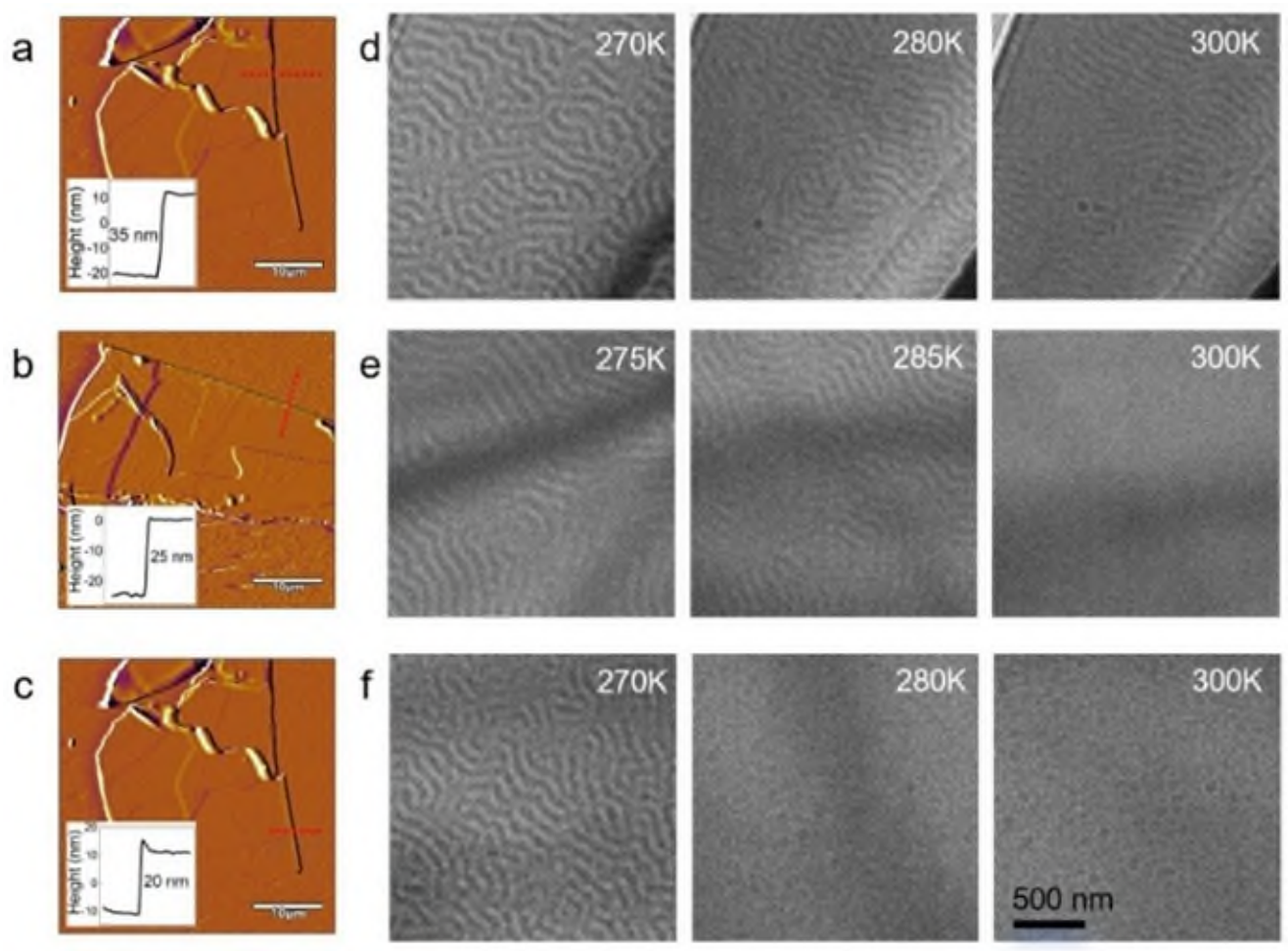


**Fig. R1.** Evolution of magnetic domains under various thicknesses (*t*) and temperature (*T*) at $Fe_{3-\delta}GaTe_2Pd_{0.1}$. **a-c,** Atomic Force Microscopy (AFM) analysis of the sample morphology and thickness of $Fe_{3-\delta}GaTe_2Pd_{0.1}$. Scale bar is 10 μm. **d-f,** The corresponding evolution of magnetic domains with decreasing temperature for three distinct sample thicknesses. Scale bar is 500 nm.

**Comment #2:** The authors demonstrate the manipulation of skyrmions using a femtosecond laser with much-lower energy than in previous reports. What is the fundamental factor that enables this ultra-low-energy manipulation? Is Pd intercalation

directly responsible for achieving such ultra-low-energy manipulation?

**Authors' Reply:** We sincerely appreciate the reviewer's insightful comments. Our work shows that femtosecond-laser-induced skyrmion generation in Pd-intercalated $Fe_{3-\delta}GaTe_2$ can be achieved with a low energy cost of ~0.6 pJ per skyrmion, which is approximately one third of that required in pristine $Fe_{3-\delta}GaTe_2$. This significant reduction of energy cost is primarily attributed to two effects introduced by Pd intercalation: (i) a reduced Curie temperature ($T_C$) and (ii) an enhanced skyrmion density. Detailed explanations are provided below.

In our study, the energy cost for per skyrmion is defined as:

$$E_{per} = E_{laser} / \rho, \tag{1}$$

where $E_{laser}$ is the total laser energy and $\rho$ is the resulting skyrmion density. Accordingly, a lowing $E_{per}$ can be achieved through a reduction of the laser energy (lower $E_{laser}$) and/or an increase in the skyrmion density (higher $\rho$). As described in the main text, femtosecond-laser-induced skyrmion generation operates through an ultrafast demagnetization process, where the laser pulse rapidly heats the sample to a temperature close to or above $T_C$, followed by skyrmion nucleation during the subsequent relaxation process. Because the required heating temperature is closely related to $T_C$, a reduced $T_C$ naturally lowers the laser power required for skyrmion generation. In Pd-intercalated sample, the introduction of Pd induces a pronounced charge redistribution throughout the material system, which weakens the exchange interaction and suppresses $T_C$, thereby enabling skyrmion generation at reduced energy.

In addition, Pd intercalation reshapes the magnetic interaction landscape by simultaneously reducing the exchange interaction constant ($J$) and enhancing the Dzyaloshinskii-Moriya interaction constant ($D$). This leads to a smaller $J/D$ ratio, favoring the stabilization of smaller skyrmions with a much higher density. Under femtosecond-laser excitation, the system experiences an ultrafast quenching process (>$10^6$-$10^8$ K/s), kinetically freezing the high-temperature magnetic texture into a metastable room-temperature configuration. Because the high-temperature phase in the Pd-intercalated sample corresponds to a lower $J/D$ ratio than that of pristine $Fe_{3-\delta}GaTe_2$, the quenched state naturally hosts denser skyrmions, which in turn reduces the energy cost for manipulating an individual skyrmion.

Therefore, Pd intercalation plays a direct and essential role in enabling the observed

energy reduction for skyrmion manipulation by simultaneously lowering the laser energy and increasing the achievable skyrmion density. Relevant discussions have been added into both the revised main text (Page 13, Lines 382-385) and the Supplementary Information (Supplementary **Note 3**).

**Comment #3:** In the demonstration of neuromorphic computing, the description of the detailed simulation procedure is completely lacking. The authors claim that the number of skyrmions encodes the synaptic weight. However, they do not explain how the experimental observations are connected to the neuromorphic computing simulations.

**Authors' Reply:** We sincerely appreciate the reviewer's insightful comments. In response to the reviewer's concern regarding the connection between the number of skyrmions and the neural network calculations, we provide a detailed description of the computation procedures below.

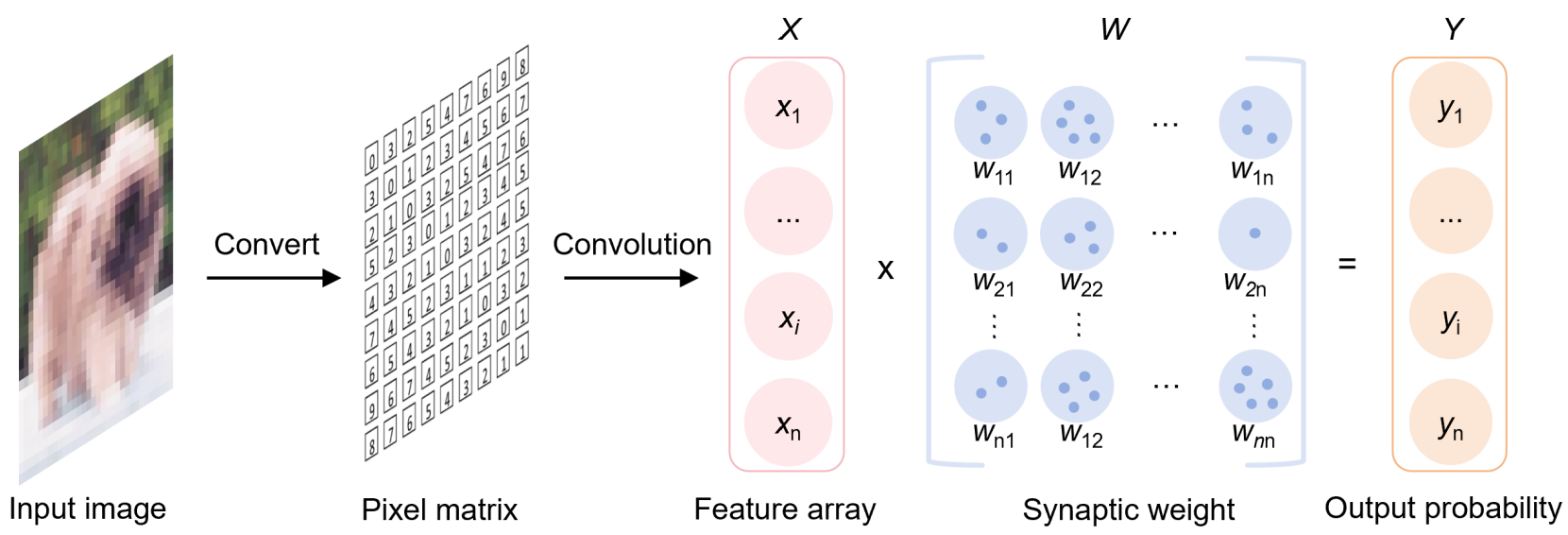


**Fig. R2.** Schematic of the neural network based on image recognition. The input image is converted into a pixel matrix and processed by convolution to obtain the feature array *X*, which is multiplied by the synaptic weight matrix *W* to produce the output probability vector *Y*. Light blue circles denote skyrmion based devices, where dark blue spheres represent the number of skyrmion.

In our work, circuit-level performance is examined using a simulated artificial neural network chip based on the Visual Geometry Group (VGG) architecture. As shown in **Fig. R2**, the input image is converted into a numerical matrix according to its pixel arrangement. Key features of the matrix are then extracted through convolution operations, and are transformed into a feature array *X*, where each element $x_i$ represents a specific feature of the image. For image classification, the input $x_i$ is weighted and summed to produce the output probability $y_i$ for each image category. The category with the highest probability is selected as the classification result. In this process, each weight factor $w_{ij}$ in the weight matrix *W* represents the relationship between image features and categories, and is a multi-valued parameter. This parameter needs to be implemented by multi-level physical states of the device in practical applications.

In the present work, a series of stable states with different number of skyrmions are obtained by applying different numbers of laser pulses. To realize hardware level image

recognition, the pulse-dependent number of skyrmions are first normalized to match the weight range of the neural network and are then used to replace the weight parameters. In practical applications, laser-driven skyrmion devices are proposed to be assembled into arrays, where each weight $w_{ij}$ in the weight matrix $W$ can be mapped onto the number of skyrmions in an individual device, thereby completing the hardware implementation of the neural network. The image classification and recognition tasks are trained on the Canadian Institute for Advanced Research-10 (CIFAR-10) dataset, which includes 10,000 images and consists of 10 image categories labeled from "0" to "9". A confusion matrix is used to evaluate the classification performance of the network, where the diagonal values indicate the proportion of correctly classified samples, and the off-diagonal values represent misclassifications. **Fig. 5d** in the main text shows the confusion matrix after a single training epoch, reflecting the network's initial untrained state with substantial classification errors across all categories. In contrast, after 100 epochs, the matrix evolves into a sharp diagonal pattern (**Fig. 5e** in the main text), indicating effective learning and accurate class discrimination, with most predictions correctly matching the true labels. The system achieves a simulated training accuracy of ~92% (**Fig. 5f** in the main text), approaching the 96% benchmark of an ideal network. The accuracy discretization can be attributed to the fact that the experimentally obtained number of skyrmion states is limited, which makes certain neighboring weight values mapped onto the same experimentally measured state.

In the revised manuscript, we have included the relevant details in the revised main text (Page 14, Lines 422-429; Page 15, Lines 430-432, 439-443, 445-452; Page 16, Line 453, Lines 463-466).

**Comment #4:** Minor comment: In the abstract, the authors state that skyrmion-based optical neuromorphic computing achieves a high accuracy of 92 %. However, this result is obtained by simulation. To avoid overstating the claim, it would be better to explicitly clarify that this is based on simulation.

**Authors' Reply:** We sincerely appreciate the reviewer's comments. In the revised manuscript, the sentence "enabling skyrmion-based optical neuromorphic computing with a high training accuracy of ~92%" has been revised into "enabling skyrmion-based optical neuromorphic computing with a simulated training accuracy of ~92%" (Page 2, Lines 47-48).

**Response to the Report of Reviewer B**

**Reviewer B's Comments:** In this manuscript, the authors present LTEM studies on Pd-intercalated non-stoichiometric $Fe_{3-\delta}GaTe_2$. They report that Pd intercalation can tune the Dzyaloshinskii–Moriya interaction (DMI), resulting in the formation of smaller skyrmions compared to pristine $Fe_{3-\delta}GaTe_2$. In addition, they demonstrate the nucleation and manipulation of skyrmion density using femtosecond laser pulses, and show that the density can be tuned with pulse length, which they suggest could be relevant for neuromorphic computations.

While the study is carefully performed, in my view, the results are incremental in nature. A closely related work by one of the corresponding authors has already been published [Nat. Commun. 15, 1017 (2024)], and the present manuscript mainly reports a quantitative reduction in skyrmion size and an increase in density upon Pd intercalation. The novelty, therefore, appears limited, as no fundamentally new insights into the skyrmion physics of $Fe_{3-\delta}GaTe_2$ are presented. Moreover, although Pd intercalation is emphasized, the role of self-intercalation is not addressed, making it difficult to disentangle the specific effect of Pd from possible intrinsic effects.

Considering the above points, I do not believe the work meets the criteria for publication in Nature Communications. However, the manuscript may be more suitable for a specialized journal that publishes incremental advances in the field of 2D magnetic materials and skyrmion physics.

**Authors' Reply:** We sincerely thank the reviewer for the careful reading of our manuscript and for raising important concerns regarding the novelty of our work and the role of Fe self-intercalation. In response, we have further highlighted the distinct scientific contributions and new physical insights of our study, making the key achievements more accessible. To clarify the specific effects of Pd intercalation and to distinguish them from possible Fe self-intercalation effects, we have conducted additional atomic-scale scanning transmission electron microscopy (STEM), electron energy loss spectroscopy (EELS), and first-principle calculations. The corresponding data, discussions and relevant references [*Nat. Commun*. **15**, 3828 (2024); *Nat. Commun*. **14**, 3744 (2023); *Sci. Adv.* **8**, eabm7103 (2022); *Phys. Rev. Mater.* **6**, 044403 (2022)] have been added into the revised manuscript. Below, we address the reviewer's comments in a point-by-point manner, and we hope that the revised manuscript and our detailed responses satisfactorily address the reviewer's concerns.

**Scientific novelty of Pd intercalation and distinction from our previous work**

While our previous work focused on the existence and optical manipulation of skyrmions in $Fe_{3-\delta}GaTe_2$, the present study addresses a fundamentally different question: how intercalation engineering can reconstruct the magnetic interaction landscape to enable low-energy, multi-level manipulation of sub-50 nm skyrmions under ultrafast excitation at room temperature. The specific advances include three aspects below.

First, the central advance of this work is the identification of a materials-design principle for skyrmion miniaturization in vdW magnets. We demonstrate that Pd intercalation simultaneously (i) weakens the Heisenberg exchange interaction and (ii) enhances the Dzyaloshinskii-Moriya interaction (DMI), resulting in a reduced ratio of the exchange constant ($J$) and DMI constant ($D$), i.e., $J/D$. This combined modification is essential for stabilizing room-temperature skyrmions with an average diameter of ~43 nm, approximately two times smaller than that in the pristine $Fe_{3-\delta}GaTe_2$ (~100 nm), representing the smallest skyrmions reported in vdW magnets to date. This work goes beyond reporting a specific material result and instead establishes lowering the $J/D$ ratio via intercalation engineering as a general and effective strategy for skyrmion miniaturization in vdW magnets, providing a broadly applicable physical framework to explore vdW skyrmionics rather than an incremental parameter optimization.

Second, the present manuscript introduces energy efficiency as a central physical metric, quantified by the energy cost per skyrmion. We demonstrate that Pd intercalation reduces this cost to ~0.6 pJ per skyrmion, approximately one-third of that in the pristine $Fe_{3-\delta}GaTe_2$. This significant reduction arises from two combined effects introduced by the Pd intercalation: (i) a reduced Curie temperature ($T_C$) and (ii) an enhanced skyrmion density, as discussed below.

In our study, the energy cost for per skyrmion is defined as:

$$E_{per} = E_{laser} / \rho, \qquad (1)$$

where $E_{laser}$ is the total energy of the laser and $\rho$ is the resulting skyrmion density. Accordingly, lowing $E_{per}$ requires both a reduced laser energy (lower $E_{laser}$) and an increased skyrmion density (higher $\rho$). Femtosecond-laser-induced skyrmion formation operates through ultrafast demagnetization process, where the laser pulse rapidly heats the sample to a temperature near or above $T_C$, enabling skyrmion nucleation during subsequent relaxation. The reduced $T_C$ in Pd-intercalated $Fe_{3-\delta}GaTe_2$ naturally lowers

the laser power required for skyrmion creation. In addition, Pd intercalation reduces the exchange interaction while enhancing the DMI. This leads to a smaller $J/D$ ratio, promoting the stabilization of smaller, denser skyrmions. Under femtosecond-laser excitation, the system experiences rapid heating followed by ultrafast quenching (>$10^6$-$10^8$ K/s), kinetically freezing the high-temperature magnetic texture into a metastable room-temperature configuration. Because the high-temperature phase in the Pd-intercalated sample corresponds to a lower $J/D$, the quenched state hosts denser skyrmions, further lowering $E_{per}$. Our work establishes a direct physical link between intercalation engineering, magnetic interactions, and energy-efficient skyrmion manipulation, advancing both the fundamental understanding of skyrmion physics and the development of skyrmion-based devices.

Finally, by tuning the number of laser pulses, we achieve a pulse-number-dependent, multi-level modulation of the skyrmion density. This work for the first time establishes a laser-induced multi-level control of skyrmions, enabling deterministic and continuously tunable skyrmion densities within a single material platform. Leveraging this functionality, we further demonstrate that such multi-level optical control of skyrmion density is well-suited for implementing neuromorphic computing based on the synapse-neuron architectures, achieving a high simulated training accuracy of ~92%. These results represent a conceptual and functional advance beyond binary skyrmion manipulation, and open a new route towards high-speed, low-power, and multi-level skyrmion-based neuromorphic computing architectures in vdW magnets, far beyond the achievements in our last work [Nat. Commun. 15, 1017 (2024)].

In summary, our results establish a framework for high-speed, low-power, and multi-level generation and utilization of densely packed, ultra-small skyrmions in vdW magnets, highlighting their potential for future neuromorphic computing architectures.

**Distinguishing Pd intercalation from Fe self-intercalation**

We highly appreciate the reviewer's comments regarding the possibility of Fe self-intercalation and a clear identification of this issue is crucial for strengthening the reliability of our conclusions. In response, we have performed additional STEM, EELS, and first-principle calculations to carefully examine the possible Fe-intercalation in the both pristine $Fe_{3-\delta}GaTe_2$ and $Fe_{3-\delta}GaTe_2Pd_{0.1}$, as discussed below.

In the previous version of the manuscript, lamellae were prepared along the [100] zone axis to probe the atomic occupancy within the vdW gaps of both $Fe_{3-\delta}GaTe_2$ and $Fe_{3-\delta}GaTe_2Pd_{0.1}$. Combined STEM imaging and energy-dispersive X-ray spectroscopy (EDX) mapping showed no evidence of Fe atoms in the vdW gaps, while the intercalated atoms in $Fe_{3-\delta}GaTe_2Pd_{0.1}$ were identified as Pd atom.

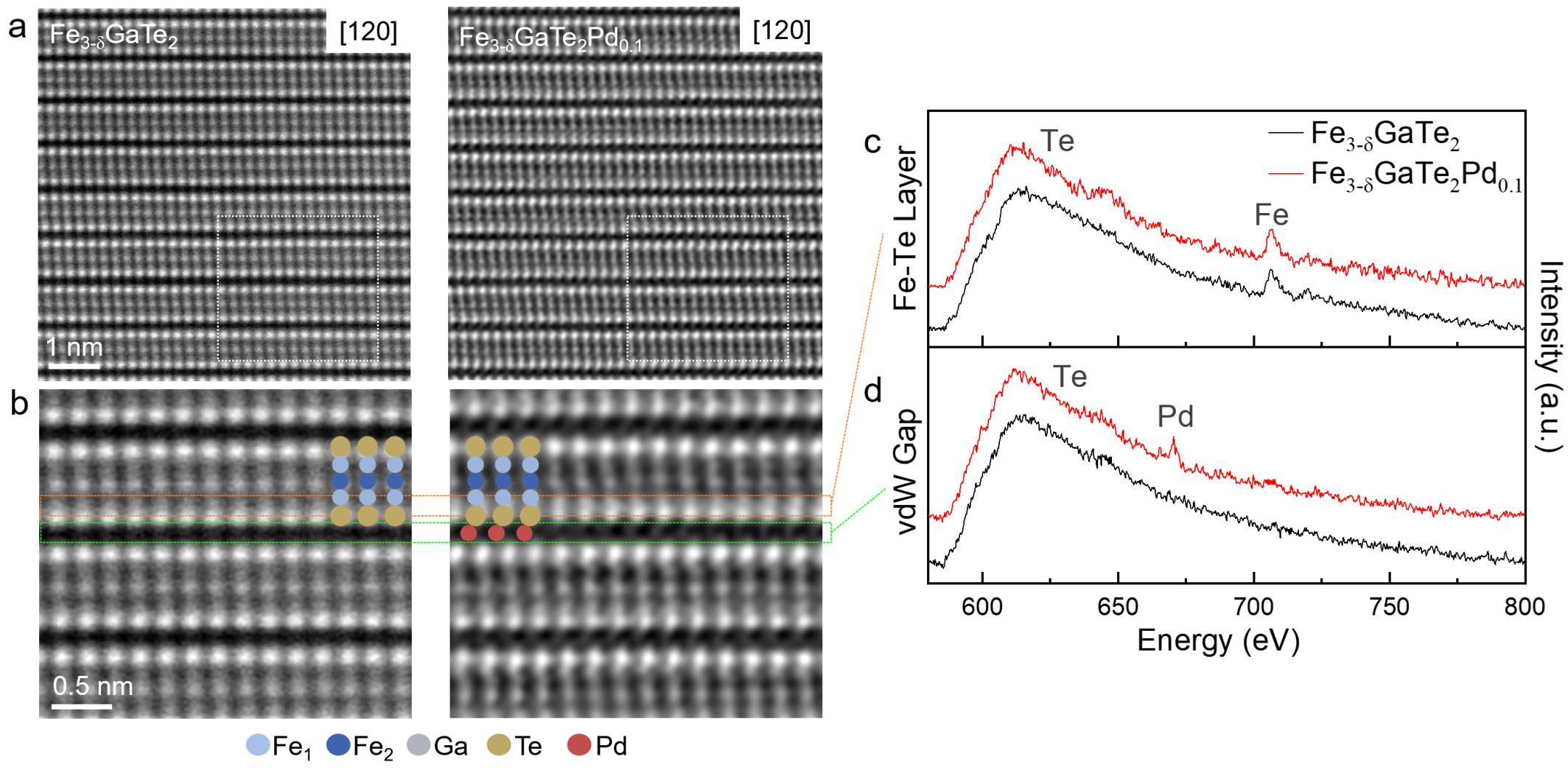


**Fig. R1.** Atomic images and EELS spectrum of $Fe_{3-\delta}GaTe_2$ and $Fe_{3-\delta}GaTe_2Pd_{0.1}$. **a**, Annular bright-field (ABF) image of taken along the [120] zone axis. Scale bars represent 1 nm. **b**, Enlarged atomic images corresponding to the regions marked in panel (a). The elements are represented as follows: $Fe_1$ (light blue), $Fe_2$ (dark blue), Te (yellow), Pd (red). Scale bars represent 0.5 nm. **c**, **d** EELS spectrum obtained from different layers, as indicated by the square boxs marked in panel **b**.

To further validate this result, we additionally performed STEM measurements on lamellae aligned along the [120] zone axis for both $Fe_{3-\delta}GaTe_2$ and $Fe_{3-\delta}GaTe_2Pd_{0.1}$. In this viewing direction, the Te-Fe-Te layers are orderly stacked along the *c*-axis (**Fig. R1a**). High-magnification images reveal no detectable contrast within the vdW gaps of pristine $Fe_{3-\delta}GaTe_2$ (**Fig. R1b**), indicating the absence of intercalated atoms. In contrast, a weak but discernible contrast is observed at the vdW gap positions in $Fe_{3-\delta}GaTe_2Pd_{0.1}$, suggesting the presence of intercalated atoms. To determine their elemental identity, we performed EELS measurements across the Fe-Te atom layer (enclosed by orange box in **Fig. R1b**) and vdW gaps (enclosed by green box in **Fig. R1b**), as indicated in **Figs. R1c** and **d**. The EELS spectra show distinct Fe-related signals from Fe-Te layer in both $Fe_{3-\delta}GaTe_2$ and $Fe_{3-\delta}GaTe_2Pd_{0.1}$ (**Fig. R1c**). In contrast, a distinct Pd-related signal is detected within the vdW gaps of $Fe_{3-\delta}GaTe_2Pd_{0.1}$, while no Fe-related signal is observed within the vdW gaps of either compound (**Fig. R1d**). These results demonstrate that the vdW gaps in $Fe_{3-\delta}GaTe_2Pd_{0.1}$ are occupied solely by Pd atoms,

with no evidence of Fe self-intercalation. This result is fully consistent with the EDX mapping results obtained in the (100) plane presented in our previous manuscript.

To confirm this result from thermodynamics, we have further calculated the formation energy ($E_f$) of Fe- and Pd-intercalated $Fe_{2.88}GaTe_2$, as schematically illustrated in **Fig. R2**. The formation energy is defined as:

$$E_f = E_{Intercalated\text{-}FGT} - (E_{FGT} + E_{Intercalated\text{-}atom}), \quad (2)$$

where $E_{Intercalated\text{-}FGT}$ is the total energy of the intercalated system, $E_{FGT}$ is the total energy of pristine $Fe_{2.88}GaTe_2$, and $E_{Intercalated\text{-}atom}$ is the energy of the isolated intercalated atom. The calculated formation energies are -5.157 eV/cell for Pd intercalation ($E_{Intercalated\text{-}Pd}$) and -4.383 eV/cell for Fe intercalation ($E_{Intercalated\text{-}Fe}$), indicating that Pd is energetically more favorable for occupying the vdW gaps of $Fe_{2.88}GaTe_2$. These results theoretically confirm that the intercalated atoms in $Fe_{3-\delta}GaTe_2Pd_{0.1}$ are Pd atom rather than Fe atom.

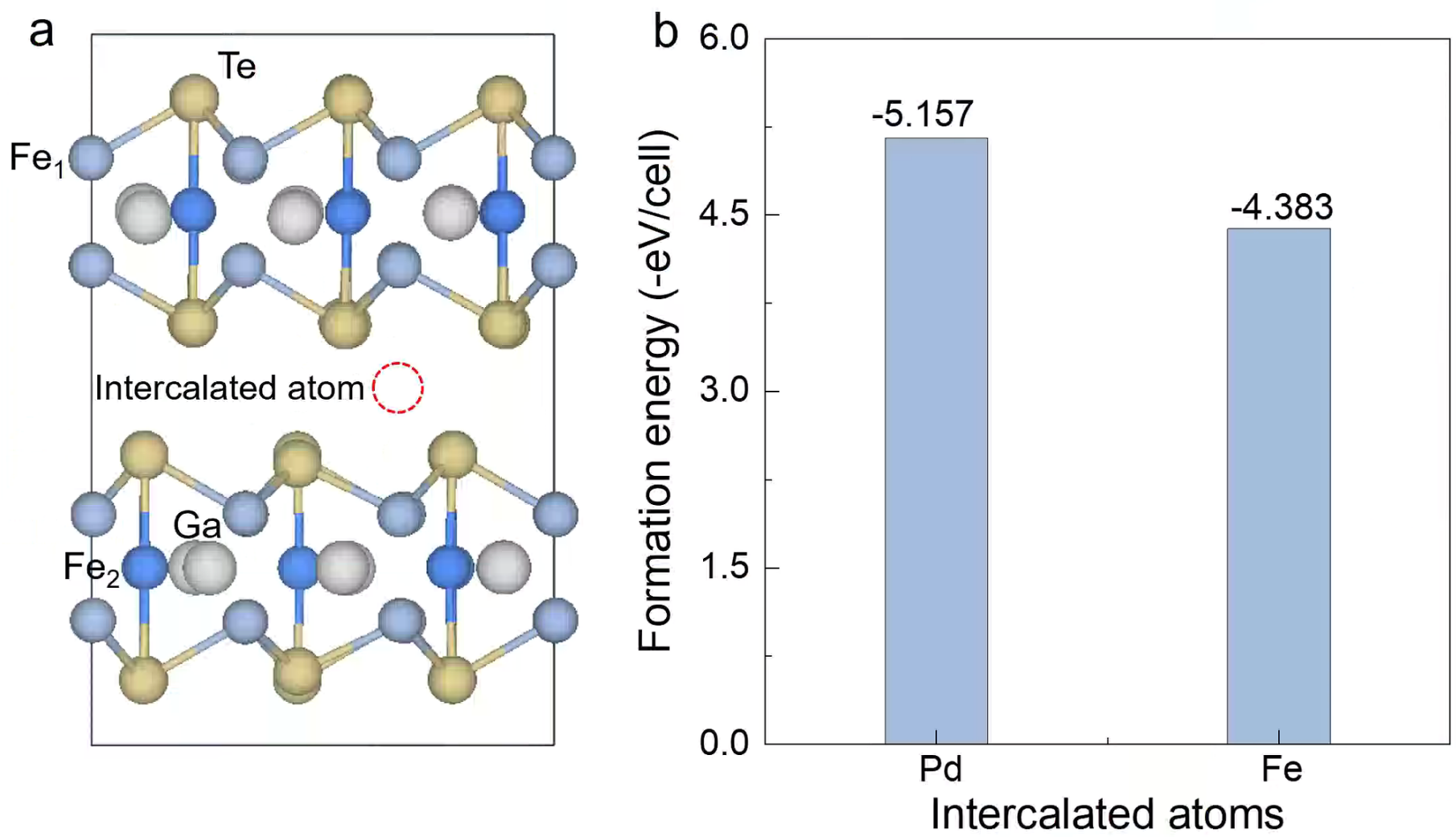


**Fig. R2. a,** Structure configuration of intercalated atoms in the gaps of the $Fe_{2.88}GaTe_2$. **b,** The calculated formation energies of Fe- and Pd-intercalated gaps of the $Fe_{2.88}GaTe_2$.

In the revised manuscript, we present a comprehensive analysis demonstrating that role of Pd intercalation and clearly distinguishing it from possible Fe self-intercalation. The corresponding analyses, discussions, and relevant references [*Nat. Commun.* **15**, 3828 (2024); *Nat. Commun.* **14**, 3744 (2023); *Sci. Adv.* **8**, eabm7103 (2022); *Phys. Rev. Mater.* **6**, 044403 (2022)] have been added into both the main text [Page 7, Lines 187-192], References (28, 40, 41, 56), Supplementary Information [Supplementary **Figs. S10, S11**].

**Response to the Report of Reviewer C**

**Reviewer C's Comment:** Through combining different theoretical and experimental methods, such as micromagnetic simulations, first-principle calculations, LTEM Measurements, the authors explored the ultrafast and multi-level control of nanoscale skyrmions in Pd-Intercalated vdW magnet $Fe_{3-\delta}GaTe_2$. They realized sub-50 nm magnetic skyrmions at room temperature with the help of magnetic field. They deemed that the enhanced DM interactions and suppressed Heisenberg exchange couplings are responsible for the size reduction of magnetic skyrmsions in Pd-Intercalated $Fe_{3-\delta}GaTe_2$. Besides, they generate magnetic skyrmions and modulate magnetic skyrmion density using femtosecond laser. This work provides insights for understanding the magnetic skyrmions in vdW magnets, and several issues are needed to be make clear.

**Authors' Reply:** We sincerely thank the reviewer for careful reading of our manuscript. We also appreciate the reviewer's valuable comments regarding the micromagnetic simulations and first-principle calculations, which are of great significance to improve the quality of our manuscript. Below we answer these comments in a point-by-point basis. We hope the reviewer will be satisfied with our revised manuscript and responses.

**Comment #1:** The micromagnetic simulation in MuMax is based on the LLG equation, so what is the damping parameter for the LLG equation in simulations? Does the damping parameter have any effect on the simulation results?

**Authors' Reply:** We thank the reviewer for pointing out this technical concern. The Landau-Lifshitz-Gilbert (LLG) equation is written as:

$$\frac{\partial \boldsymbol{m}}{\partial t} = -|\gamma|\boldsymbol{m}\times\boldsymbol{H}_{\mathrm{eff}} + \alpha\boldsymbol{m}\times\frac{\partial \boldsymbol{m}}{\partial t}, \quad (1)$$

where $\boldsymbol{m}$ is the normalized magnetization vector ($\boldsymbol{M}/M_s$), $\gamma$ is the gyromagnetic ratio, $\boldsymbol{H}_{\mathrm{eff}}$ is the effective field and $\alpha$ is the Gilbert damping parameter. The first term describes energy-conserving precession of the magnetization around the effective field, while the second term represents the damping torque that dissipates energy and drives the system towards an equilibrium state aligned with the effective field. Thus, a smaller $\alpha$ leads to stronger precession and a longer relaxation process, whereas a larger $\alpha$ suppresses precession and accelerates relaxation. In our simulations, $\alpha$ was set to be 0.1, following the experimentally reported value of 0.075 for $Fe_3GaTe_2$ at 300 K [*Nano Lett.*, **24**, 12204 (2024)]. To examine the influence of $\alpha$ on the micromagnetic simulations, we systematically simulated the magnetic field-dependent domain evolution processes

for $\alpha$ ranging from 1 to 0.05, as shown in **Fig. R1**. These results indicate that varying $\alpha$ has a negligible impact on the final relaxed magnetic configurations. This behavior is expected because the magnetic states discussed here correspond to equilibrium states, which are governed by the energy landscape rather than the specific value of α. In the revised manuscript, detailed descriptions about $\alpha$ have been added into the Methods (Page 17, Line 496-497) and Supplementary **Note 1**.

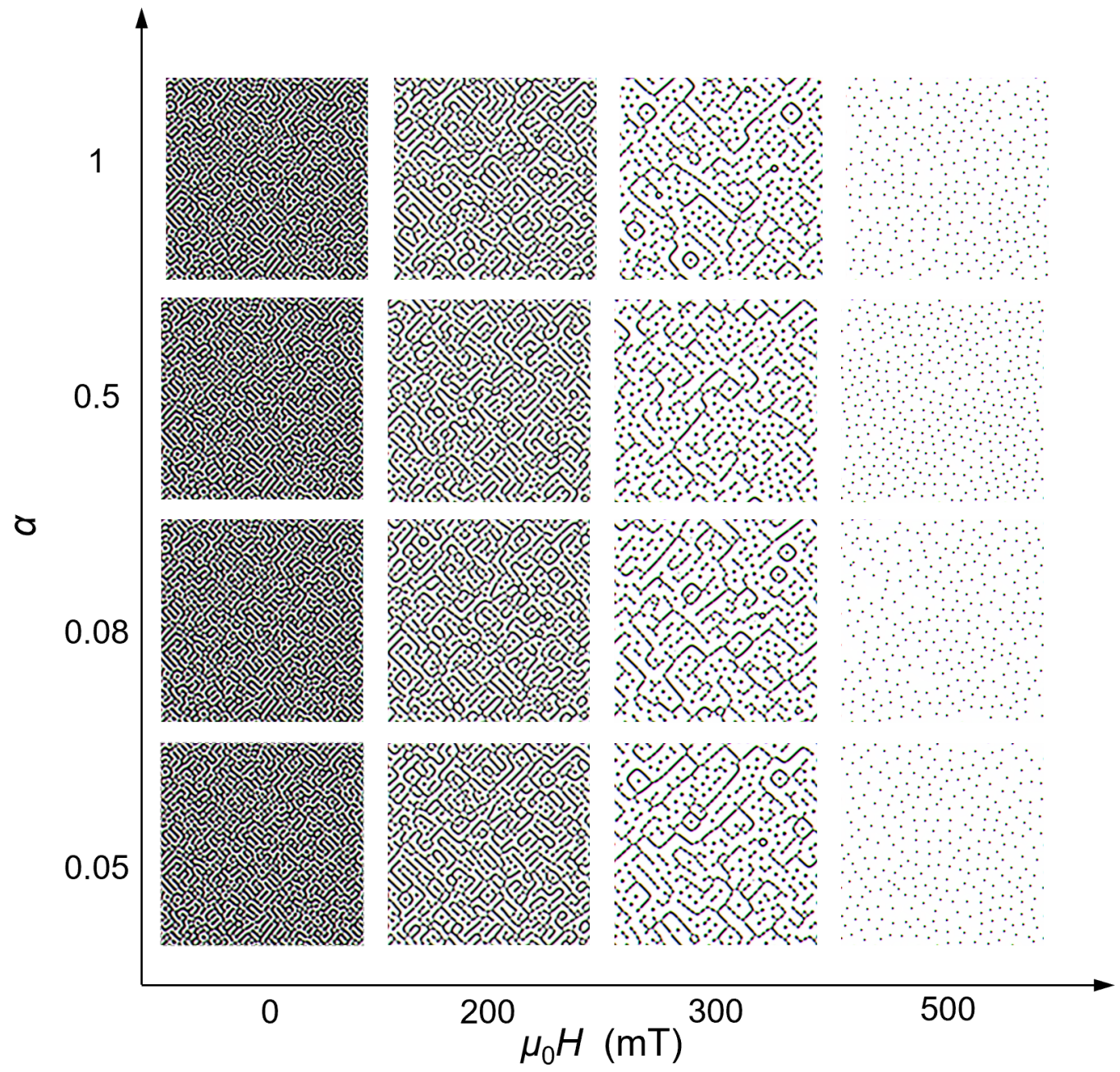


**Fig. R1.** Magnetic field driven evolution of magnetic domains under various damping parameter $\alpha$. $M_s$, $K_{eff}$, $J$ and $D$ are fixed to be $2.5\times10^5$ A/m, $1.5\times10^5$ J/m$^3$, 0.7 pJ/m, 0.5 mJ/m$^2$.

**Comment #2:** It looks that the Heisenberg exchange $J$, DM interactions $D$ in Figure 2d are obtained by experiments. In Supplementary Table 3 and 4, the Heisenberg exchange constant ($J$) and DM interaction ($D$) for both $Fe_{2.88}GaTe_2Pd_{0.05}$ and $Fe_{2.88}GaTe_2$ are given. Are the experimental and theoretical results consistent?

**Authors' Reply:** We sincerely appreciate the reviewer's insightful comments, which are of great significance to improve the consistency between the experimentally extracted and theoretically calculated magnetic interaction parameters. Based on the reviewer's comments, we have carefully examined our computational approach and calculated the magnetic interactions using several different exchange-correlation schemes, including GGA, GGA + U, LDA, and LDA + U. We find that, although the absolute values obtained from first-principle calculations differ from the experimentally extracted ones, all approaches consistently reproduce the same evolution trend. The detailed analysis is presented below.

## Heisenberg exchange interaction

In our work, the experimentally extracted Heisenberg exchange constant at 10 K, i.e. $J$ (10 K), decreases from 1.89 pJ/m for $Fe_{2.88}GaTe_2$ to 1.79 pJ/m for $Fe_{2.88}GaTe_2Pd_{0.05}$, indicating that Pd intercalation weakens the ferromagnetic exchange coupling. To understand the physics mechanism underlying this trend, we calculated the nearest-neighbor, next-nearest-neighbor, and interlayer exchange coupling constants between Fe atoms (denoted as $J_1$, $J_2$, and $J_3$, respectively) for both $Fe_{2.88}GaTe_2$ and $Fe_{2.88}GaTe_2Pd_{0.05}$ using first-principle methods within the GGA framework in the previous version of our manuscript. As shown in **Table R1**, the calculated nearest-neighbor exchange $J_1$ decreases from 140.019 meV (93.093 pJ/m) in $Fe_{2.88}GaTe_2$ to 132.148 meV (87.999 pJ/m) in $Fe_{2.88}GaTe_2Pd_{0.05}$, reproducing the experimentally observed reduction of ferromagnetic exchange upon Pd intercalation. However, the absolute magnitudes of the calculated exchange constants are significantly larger than those extracted experimentally. To further examine this discrepancy, we calculated the exchange interactions using different exchange-correlation schemes, including GGA + U, LDA, and LDA + U, where U is the effective on-site Coulomb interaction. We find that the reduction of ferromagnetic exchange induced by Pd intercalation is robust for all these methods, confirming the reliability of our theoretical model. In contrast, introducing a U significantly reduces the magnitude of $J$, bringing the calculated values closer to the experimental estimates. In particular, when a moderate U = 2.5 eV is applied within the GGA model, $J_1$ decreases to 31.732 meV (21.131 pJ/m) for $Fe_{2.88}GaTe_2Pd_{0.05}$. Further increasing U up to 3.0 eV, the self-consistent calculations become difficult to converge. Therefore, GGA + U approach with U = 2.5 eV is adopted in subsequent calculations.

We note that, when applying GGA + U (U = 2.5 eV), the calculated exchange constant $J_1$ falls within the range reported in previous theoretical studies (22 meV-75 meV) [*Nat Commun* **15**, 10765 (2024), *Nano Lett.* **25**, 11, 4260 (2025), *Nano Lett.* **23**, 11526 (2023)]. However, it remains larger than the experimental values. In experiments, $J$ was extracted by fitting the equation [*Nat. Phys.* **11**, 825-829 (2015); *Nat. Commun.* **15** 4472 (2024)]:

$$J\,(10\ \mathrm{K}) = \frac{M_{\mathrm{s}}\,(10\ \mathrm{K})\,\lambda_{\mathrm{spin}}\,(10\ \mathrm{K})}{2gu_{\mathrm{B}}}, \quad (2)$$

where $J$ scales with the saturation magnetization $M_s$. Therefore, the smaller experimentally extracted $J$ primarily originates from the reduced $M_s$ in real samples compared with that obtained from first-principle calculations. Specifically, the experimentally measured $M_s$ of $Fe_{3-\delta}GaTe_2Pd_{0.05}$ is 52.0 emu/g at 10 K, whereas the calculated $M_s$ based on the GGA + U (U = 2.5 eV) method reaches 83.8 emu/g. The reduced $M_s$ in real samples can be attributed to the higher concentration of Fe vacancies compared with the ideal structures used in the first-principle calculations.

In the revised manuscript, we present the GGA + U (U = 2.5 eV) results and include a detailed discussion in the revised Supplementary Information (Supplementary **Note 2** and Supplementary **Table S4**) to clarify the influence of different exchange-correlation treatments, enabling a more consistent comparison between the experimental and theoretical results.

**Table R1.** Calculated values of $J_1$, $J_2$, and $J_3$ for $Fe_{2.88}GaTe_2$ and $Fe_{2.88}GaTe_2Pd_{0.05}$ based on first-principle calculations using GGA, GGA+U, LDA, and LDA+U functionals.

| Functionals | Structure | $J_1$ (meV) | $J_1$ (pJ/m) |
|---|---|---|---|
| GGA | $Fe_{2.88}GaTe_2$ | 140.019 | 93.093 |
| | $Fe_{2.88}GaTe_2Pd_{0.05}$ | 132.148 | 87.999 |
| GGA+U (U =1 eV) | $Fe_{2.88}GaTe_2$ | 149.568 | 99.442 |
| | $Fe_{2.88}GaTe_2Pd_{0.05}$ | 142.261 | 94.733 |
| GGA+U (U = 2 eV) | $Fe_{2.88}GaTe_2$ | 48.937 | 32.536 |
| | $Fe_{2.88}GaTe_2Pd_{0.05}$ | 43.878 | 29.219 |
| GGA+U (U = 2.5 eV) | $Fe_{2.88}GaTe_2$ | 36.431 | 24.222 |
| | $Fe_{2.88}GaTe_2Pd_{0.05}$ | 31.732 | 21.131 |
| GGA+U (U = 3 eV) | $Fe_{2.88}GaTe_2$ | Difficult to converge | |
| | $Fe_{2.88}GaTe_2Pd_{0.05}$ | | |
| LDA | $Fe_{2.88}GaTe_2$ | 139.105 | 93.034 |
| | $Fe_{2.88}GaTe_2Pd_{0.05}$ | 88.476 | 59.903 |
| LDA+U (U = 1 eV) | $Fe_{2.88}GaTe_2$ | 163.917 | 109.628 |
| | $Fe_{2.88}GaTe_2Pd_{0.05}$ | 124.925 | 84.581 |
| LDA+U (U = 2 eV) | $Fe_{2.88}GaTe_2$ | 136.590 | 91.351 |
| | $Fe_{2.88}GaTe_2Pd_{0.05}$ | 92.181 | 62.412 |
| LDA+U (U = 2.5 eV) | $Fe_{2.88}GaTe_2$ | 118.923 | 79.536 |
| | $Fe_{2.88}GaTe_2Pd_{0.05}$ | 76.690 | 51.923 |

**Dzyaloshinskii-Moriya interaction**

**Fig. 2d** in the main text shows that the experimentally extracted DMI constant (*D*) at 300 K increases with Pd intercalation from ~0.15 mJ/m$^2$ for $Fe_{3-\delta}GaTe_2$ to ~0.20 mJ/m$^2$ for $Fe_{3-\delta}GaTe_2Pd_{0.05}$, demonstrating that Pd intercalation enhances the DMI. For the non-stoichiometric $Fe_{3-\delta}GaTe_2$, the Fe deficiency leads to a deviation of $Fe_2$ atoms towards *c*-axis direction, which breaks the inversion symmetry and results in the DMI [*Nat. Commun.* **15**, 1017 (2024)]. Therefore, the DMI between the $Fe_1$ atom pairs and Te atoms are neglected, and that between the $Fe_2$ atom pairs and Te atom in $Fe_{2.88}GaTe_2$ and $Fe_{2.88}GaTe_2Pd_{0.05}$ are calculated using the four-state method [*Dalton Trans*. **42**, 823 (2013); *Phys. Rev. B* **84**, 224429 (2011)].

Based on the GGA + U (U = 2.5 eV) model, the absolute value of *D* for $Fe_2$ pairs nearest to Pd atom are calculated. The resulting *D* values are 0.495 mJ/m$^2$ for $Fe_{2.88}GaTe_2$ and 1.368 mJ/m$^2$ (calculations details are presented in the Reply to reviewer's comment #5) for $Fe_{2.88}GaTe_2Pd_{0.05}$, respectively, supporting the experimentally observed enhancement of *D*. Despite the consistency in the evolution trend, the calculated *D* are larger than that extracted experimentally. This discrepancy can be attributed to two primary factors. First, the calculated DMI corresponds to the ground-state value at 0 K, whereas the experimental DMI is extracted at room temperature, where thermal fluctuations generally reduce the effective DMI [*Phys. Rev. B* 101, 054433 (2020); *Appl. Phys. Lett.* 113, 092402 (2018)]. Second, the experimentally extracted DMI represents a spatially averaged value over the sample, and thus can be reduced by inhomogeneous Pd distribution in real materials.

In the revised manuscript, the relevant data and discussions have been added into the revised Supplementary Information (Supplementary **Fig. S16** and **Tables S5, S6**).

**Comment #3:** The calculated $J_1$ of both $Fe_{2.88}GaTe_2Pd_{0.05}$ and $Fe_{2.88}GaTe_2$ in Supplementary Table 3 are so large that I suggest the author should make a double check on this.

**Authors' Reply:** We sincerely appreciate the reviewer's insightful comments, which are of great significance to improve the quality of our manuscript. In this work, we calculated the nearest-neighbor, next-nearest-neighbor, and interlayer exchange coupling constants between Fe atoms (denoted as $J_1$, $J_2$, and $J_3$, respectively, **Fig. R2a**) for both $Fe_{2.88}GaTe_2$ and $Fe_{2.88}GaTe_2Pd_{0.05}$. Details procedures are listed below.

First, the magnetic ground state of $Fe_{2.88}GaTe_2$ and $Fe_{2.88}GaTe_2Pd_{0.05}$ was determined

by comparing the energies of four magnetic configurations, as displayed in **Figs. R2b-e**. We find that ferromagnetic state (**Fig. R2b**) is always more stable than the other three states (**Figs. R2c-e**), suggesting that these compounds exhibit ferromagnetism. Then, we analyzed all symmetry-inequivalent intralayer $Fe_1$-$Fe_1$ bond lengths in both $Fe_{2.88}GaTe_2$ and $Fe_{2.88}GaTe_2Pd_{0.05}$. Despite the lowered crystal symmetry, our results demonstrate that the variations in $Fe_1$-$Fe_1$ bond lengths are relatively small. Specifically, in $Fe_{2.88}GaTe_2$ ($Fe_{2.88}GaTe_2Pd_{0.05}$), $Fe_1$-$Fe_1$ distances associated with the nearest-neighbor exchange coupling constants ($J_1$) range from 2.370 Å to 2.448 Å (from 2.369 Å to 2.455 Å), corresponding to variations of ~0.078 Å (~0.086 Å). These results indicate that the intralayer $Fe_1$-$Fe_1$ bonding environment does not give rise to strongly inequivalent exchange parameters. Based on these results, the ferromagnetism is mapped onto a classical Heisenberg model.

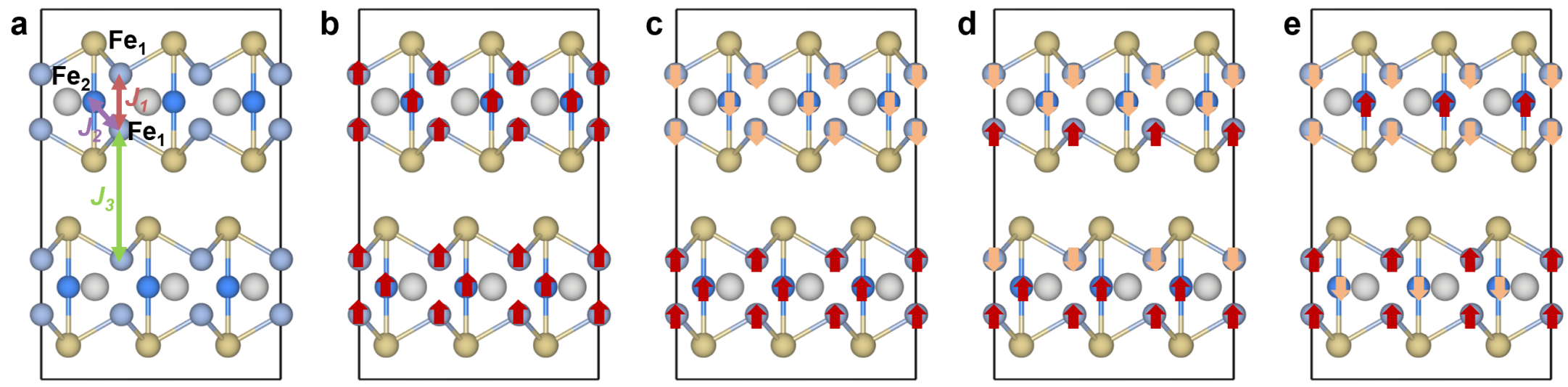


**Fig. R2. a,** Defined exchange parameter. **b-e** Schematic illustration of four magnetic configurations. Red arrows indicate spins pointing upward, whereas pink arrows indicate spins pointing downward. The total energies of the four magnetic configurations correspond to $E_1$, $E_2$, $E_3$, $E_4$.

The Heisenberg Hamiltonian for our calculations is expressed as:

$$H = -J_1 \sum_{i,j} \vec{S_i} \cdot \vec{S_j} - J_2 \sum_{k,l} \vec{S_k} \cdot \vec{S_l} - J_3 \sum_{m,n} \vec{S_m} \cdot \vec{S_n} - K\left(S^Z\right)^2 \quad (2)$$

where $S$ denotes the magnetic moment at the corresponding Fe site, and $K$ is the anisotropic energy parameter. $J_1$, $J_2$, and $J_3$ are obtained from the total energies of one ferromagnetic configuration (schematically illustrated in **Fig. R2b**, $E_1$) and three other states (schematically illustrated in **Figs. R2c-e**, $E_2$, $E_3$, $E_4$) following the classical Heisenberg Hamiltonian, as described below:

$$E_1 = -17J_1S_1^2 - 99J_2S_1S_2 - 17J_3S_1^2 + E_0, \quad (3)$$

$$E_2 = -17J_1S_1^2 - 99J_2S_1S_2 + 17J_3S_1^2 + E_0, \quad (4)$$

$$E_3 = 17J_1S_1^2 - 3J_2S_1S_2 + 17J_3S_1^2 + E_0, \quad (5)$$

$$E_4 = -17J_1S_1^2 + 99J_2S_1S_2 + 17J_3S_1^2 + E_0, \quad (6)$$

where $E_0$ represents the energy of the nonmagnetic state, $S_1$ and $S_2$ represent the spin

magnetic moments of $Fe_1$ and $Fe_2$ atoms. By combing equations (3)-(6), we can deduce $J_1$, $J_2$, and $J_3$ to be:

$$J_1 = (E_3 - \frac{48}{99}E_4 - \frac{51}{99}E_2)/34S_1^2, \quad (7)$$

$$J_2 = (E_4 - E_2)/198S_1S_2, \quad (8)$$

$$J_3 = (E_2 - E_1)/34S_1^2. \quad (9)$$

The calculated $J_1$ values for $Fe_{2.88}GaTe_2$ and $Fe_{2.88}GaTe_2Pd_{0.05}$ are 140.019 meV (93.093 pJ/m) and 132.148 meV (87.999 pJ/m), respectively using first-principle calculations within the GGA framework. While the calculations reproduce the experimentally observed reduction of ferromagnetic exchange upon Pd intercalation, the absolute magnitudes of the calculated exchange constants are significantly larger than those extracted experimentally.

Based on the reviewer's comments, we have carefully examined our computational approach and calculated the magnetic interactions using several different exchange-correlation schemes, including GGA, GGA + U, LDA, and LDA + U (**Table R1** in the response to reviewer's comment #2). We find that the reduction of ferromagnetic exchange induced by Pd intercalation is robust for all these methods, confirming the reliability of our theoretical model. In contrast, introducing U significantly reduces the magnitude of *J*, bringing the calculated values closer to the experimental estimates. In particular, when a moderate U = 2.5 eV is applied, $J_1$ of $Fe_{2.88}GaTe_2Pd_{0.05}$ decreases to 31.732 meV (21.131 pJ/m). Further increasing U up to 3.0 eV, the self-consistent calculations become difficult to converge.

We note that, when applying GGA + U (U = 2.5 eV), the calculated exchange constant $J_1$ falls within the range reported in previous theoretical studies (22 meV-75 meV) [*Nat Commun* **15**, 10765 (2024), *Nano Lett.* **25**, 11, 4260 (2025), *Nano Lett.* **23**, 11526 (2023)]. However, it remains larger than the experimental values. The smaller experimentally extracted *J* originates from the reduced $M_s$ in real samples, which can be attributed to the substantially higher concentration of Fe vacancies compared with the idealized structures used in the first-principle calculations.

In the revised manuscript, we present the GGA + U (U = 2.5 eV) results and include a detailed discussion in the revised Supplementary Information (Supplementary **Note 2** and Supplementary **Table S4**) to clarify the influence of different exchange-

correlation treatments, enabling a more consistent comparison between the experimental and theoretical results.

**Comment #4:** The authors used only $J_1$, $J_2$ and $J_3$ for $Fe_{2.88}GaTe_2Pd_{0.05}$ and $Fe_{2.88}GaTe_2$. This should be not reasonable because $Fe_{2.88}GaTe_2Pd_{0.05}$ and $Fe_{2.88}GaTe_2$ has a relative low symmetry. Such rough classification of Heisenberg exchanges may cause un-reasonable calculation results.

**Authors' Reply:** We sincerely thank the reviewer for this insightful comment regarding the treatment of Heisenberg exchange interactions in systems with reduced symmetry. In response, we carefully analyzed all the symmetry-inequivalent intralayer $Fe_1$-$Fe_1$ bond lengths in both $Fe_{2.88}GaTe_2$ and $Fe_{2.88}GaTe_2Pd_{0.05}$. Despite the lowered crystal symmetry, we find that the variations in $Fe_1$-$Fe_1$ bond lengths are relatively small, indicating that the local magnetic exchange environment remains nearly uniform.

Specifically, in $Fe_{2.88}GaTe_2$ ($Fe_{2.88}GaTe_2Pd_{0.05}$), $Fe_1$-$Fe_1$ distances associated with the nearest-neighbor exchange coupling constants ($J_1$) range from 2.370 Å to 2.448 Å (from 2.369 Å to 2.455 Å), corresponding to variations of ~0.078 Å (~0.086 Å ). To further quantify the impact of these bond-length variations, we calculated $J_1$ for the shortest and longest $Fe_1$-$Fe_1$ distances (denoted as $J_{1\text{-shortest}}$ and $J_{1\text{-longest}}$) in both systems based on four-state energy-mapping method [*Dalton Trans.* 42, 823 (2013)], as summarized in **Table R2**. We find that, the bond-length variations lead to only slight variations in $J_1$. Specifically, in $Fe_{2.88}GaTe_2$, $J_1$ varies from 35.040 meV to 37.889 meV, while in $Fe_{2.88}GaTe_2Pd_{0.05}$, it ranges from 30.964 meV to 34.554 meV. The slight variations of $J_1$ demonstrate that, although the systems lack high crystal symmetry, the intralayer $Fe_1$-$Fe_1$ bonding environment does not give rise to strongly inequivalent exchange parameters. Therefore, grouping the dominant magnetic interactions into nearest-neighbor ($J_1$), next-nearest-neighbor ($J_2$), and interlayer ($J_3$) exchange constants provides a physically reasonable and effective description of the magnetic interactions in both systems. Importantly, this simplified classification captures the dominant exchange pathways and correctly reproduces the experimentally observed trends, confirming the reliability of our model.

The bond-length analysis has been added into the revised Supplementary Information (Supplementary **Note 2**) to support the validity of the $J_1$-$J_3$ approximation.

**Table R2.** Exchange couplings $J_1$ obtained from the four-state energy-mapping method for $Fe_{2.88}GaTe_2$ and $Fe_{2.88}GaTe_2Pd_{0.05}$. In these low-symmetry structures, the nearest-neighbor $Fe_1$-$Fe_1$

bond lengths are not equivalent. $J_{1\text{-shortest}}$ and $J_{1\text{-longest}}$ denote the couplings associated with the shortest and longest $Fe_1$-$Fe_1$ nearest-neighbor pairs, respectively.

| Structure | $J_{1\text{-longest}}$ (meV) | $J_{1\text{-shortest}}$ (meV) |
|---|---|---|
| $Fe_{2.88}GaTe_2$ | 35.040 | 37.889 |
| $Fe_{2.88}GaTe_2Pd_{0.05}$ | 30.964 | 34.554 |

**Comment #5:** The calculated DM interactions given in figure 2d and Supplementary Table 4 should use the same units, so it is easy for reader to compare the experimental and theoretical results. Explicitly, are the experimental and theoretical result of DM interactions consistent?

**Authors' Reply:** We sincerely appreciate the reviewer's comments. In the revised manuscript, the unit of the DMI constant ($D$) used in both the experimental measurements and theoretical calculations has been unified to mJ/m$^2$, in order to facilitate a direct comparison.

**Fig. 2d** in the main text shows that the experimentally extracted DMI constant at 300 K increases with Pd intercalation from ~0.15 mJ/m² for x = 0 to ~0.20 mJ/m² for x = 0.05, demonstrating that Pd intercalation enhances the DMI. For the non-stoichiometric $Fe_{3-\delta}GaTe_2$ and $Fe_{3-\delta}GaTe_2Pd_x$, the Fe deficiency leads to a deviation of $Fe_2$ atoms towards $c$-axis direction, which breaks the inversion symmetry and results in the DMI [*Nat. Commun.* **15**, 1017 (2024)]. Therefore, the DMI between the $Fe_1$ atom pairs and Te atoms are neglected, and that between the $Fe_2$ atom pairs and Te atom in $Fe_{2.88}GaTe_2$ and $Fe_{2.88}GaTe_2Pd_{0.05}$ is calculated based on a 3×3 supercell, as schematically illustrated in **Fig. R3**.

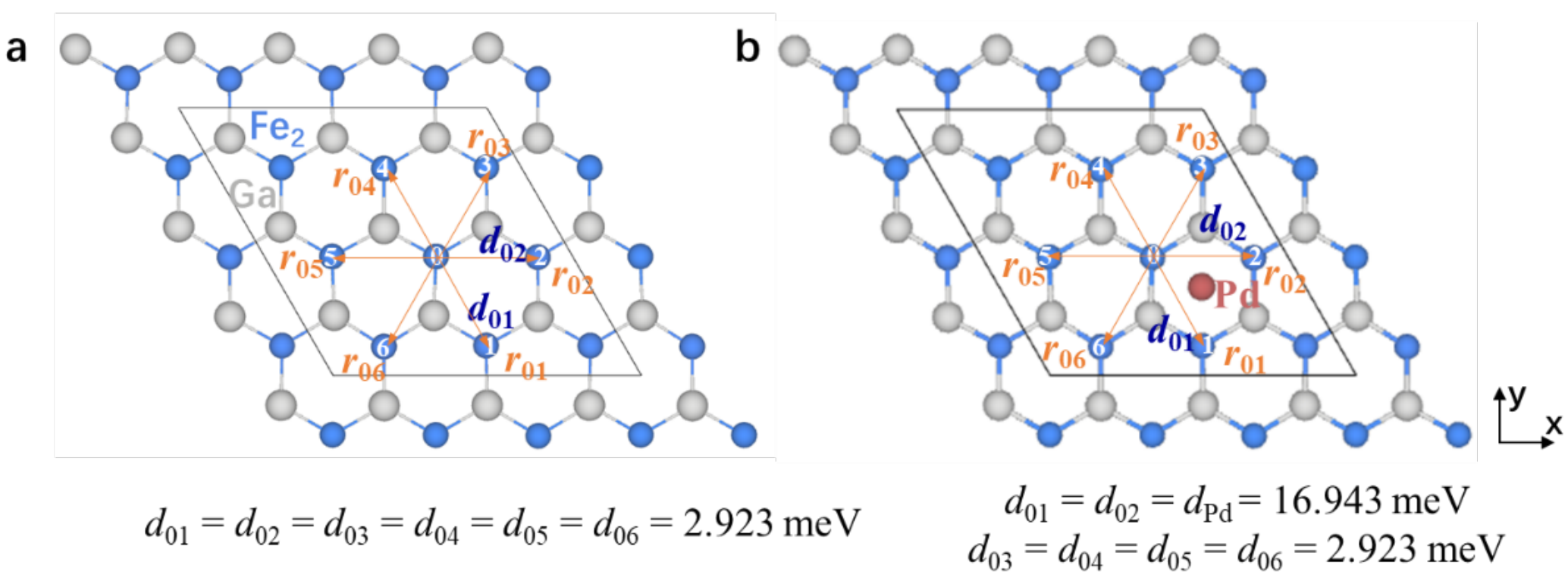


**Fig. R3.** Schematic diagram of the DMI parameters $d_{0j}$ and direction vector $\mathbf{r}_{0j}$ between different $Fe_2$-$Fe_2$ pairs in **a** $Fe_{2.88}GaTe_2$ and **b** $Fe_{2.88}GaTe_2Pd_{0.05}$.

In our study, $D$ is obtained via the equation [*Nat. Rev. Phys.* **5**, 43 (2022)]:

$$D = \sqrt{D_x^2 + D_y^2 + D_z^2}\ , \tag{10}$$

where $D_x$, $D_y$, $D_z$ are the Cartesian components of $D$ and are given by

$$D_{i\ (i = x,\ y,\ z)} = \frac{1}{V}\sum_j |(\boldsymbol{r}_{0j})_i| d_{0j} \quad . \tag{11}$$

Here, $V$ represent the volume of the 3×3 supercell and is $2.01\times10^{-27}$ $m^3$ for both $Fe_{2.88}GaTe_2$ and $Fe_{2.88}GaTe_2Pd_{0.05}$; $\boldsymbol{r}_{0j} = \boldsymbol{r}_j - \boldsymbol{r}_0$ is the direction vector pointing from the reference $Fe_2$ atom at site 0 to its neighboring $Fe_2$ atom at site j (**Fig. R3**), and $(\boldsymbol{r}_{0j})_i$ (i = x, y, z) denotes the component of direction vector along x-, y-, z-axis, respectively; $d_{0j}$ represents the DMI energy between reference $Fe_2$ atom at site 0 and the nearest-neighbor $Fe_2$ atoms at site j (**Fig. R3**). The magnitude of $\boldsymbol{r}_{0j}$ and their Cartesian components are listed in **Table R3**.

**Table R3.** Direction vectors $\boldsymbol{r}_{0j}$ and their components along the x-, y-, z-axis for $Fe_{2.88}GaTe_2$ and $Fe_{2.88}GaTe_2Pd_{0.05}$

| Atom | | $\lvert\boldsymbol{r}_{0j}\rvert$ (Å) | $\lvert(\boldsymbol{r}_{0j})_x\rvert$ (Å) | $\lvert(\boldsymbol{r}_{0j})_y\rvert$ (Å) | $\lvert(\boldsymbol{r}_{0j})_z\rvert$ (Å) |
|---|---|---|---|---|---|
| $Fe_{2.88}GaTe_2$ | 1 | 3.99 | 1.96 | 3.47 | 0.04 |
| | 2 | 3.98 | 2.02 | 3.43 | 0.04 |
| | 3 | 3.99 | 1.97 | 3.47 | 0.03 |
| | 4 | 4.05 | 4.05 | 0.00 | 0.00 |
| | 5 | 4.05 | 2.02 | 3.51 | 0.00 |
| | 6 | 3.99 | 3.99 | 0.02 | 0.02 |
| $Fe_{2.88}GaTe_2Pd_{0.05}$ | 1 | 3.98 | 1.96 | 3.47 | 0.07 |
| | 2 | 3.99 | 2.03 | 3.44 | 0.06 |
| | 3 | 4.00 | 1.96 | 3.48 | 0.04 |
| | 4 | 3.98 | 3.98 | 0.00 | 0.00 |
| | 5 | 4.05 | 2.03 | 3.51 | 0.00 |
| | 6 | 3.98 | 0.02 | 0.06 | 3.98 |

As shown in **Table R3**, the distances between nearest-neighbor $Fe_2$ pairs in $Fe_{2.88}GaTe_2$ are nearly the same. Therefore, for simplicity, we assume a uniform DMI energy $d_{0j} = d$ for all nearest-neighbor $Fe_2$ pairs in $Fe_{2.88}GaTe_2$. Based on the four-state method and GGA + U (U = 2.5 eV) model [*Dalton Trans.* **42**, 823 (2013); *Phys. Rev. B* **84**, 224429 (2011)], $d$ is calculated to be 2.923 meV.

For $Fe_{2.88}GaTe_2Pd_{0.05}$, the nearest-neighbor $Fe_2$-$Fe_2$ distances remain almost unchanged upon Pd intercalation. However, the presence of Pd provides additional spin-orbit coupling to nearby $Fe_2$ pairs, leading to an enhancement of the DMI energies. Accordingly, we assume that the DMI energy for $Fe_2$-$Fe_2$ pairs farther from the Pd

atoms remian unchanged ($d_{03}$= $d_{04}$ = $d_{05}$ = $d_{06}$ = 2.923 meV), while the DMI energy for $Fe_2$ pairs adjacent to the Pd atoms is enhanced, with $d_{01}$ = $d_{02}$ = $d_{Pd}$ = 16.943 meV.

Based on equations (10) and (11), and using the DMI energies together with the corresponding direction vectors, the DMI constant $D$ can are calculated to be 0.495 mJ/m$^2$ for $Fe_{2.88}GaTe_2$ and 1.368 mJ/m$^2$ for $Fe_{2.88}GaTe_2Pd_{0.05}$. These results demonstrate that Pd intercalation enhances the DMI, consistent with the experimentally extracted values. Detailed numerical results are summarized in **Table R4**.

**Table R4.** Calculated total DMI constant $d$ and $D$ and the components of $D$ along the x-, y-, z-axis in $Fe_{2.88}GaTe_2$ and $Fe_{2.88}GaTe_2Pd_{0.05}$.

| | $Fe_{2.88}GaTe_2$ | $Fe_{2.88}GaTe_2Pd_{0.05}$ |
|---|---|---|
| $d_{0j}$ (meV) | $d_{01} = d_{02} = d_{03} = d_{04} = d_{05} = d_{06}$ = 2.923 | $d_{01} = d_{02} = d_{Pd}$ = 16.943<br>$d_{03} = d_{04} = d_{05} = d_{06}$ = 2.923 |
| $D_x$ (mJ/m$^2$) | 0.374 | 0.818 |
| $D_y$ (mJ/m$^2$) | 0.372 | 1.097 |
| $D_z$ (mJ/m$^2$) | 0.003 | 0.002 |
| $D$ (mJ/m$^2$) | 0.495 | 1.368 |

Despite the consistency in the evolution trend, the calculated $D$ are larger than that extracted experimentally. This discrepancy can be attributed to two primary factors. First, the calculated DMI corresponds to the ground-state value at 0 K, whereas the experimental DMI is extracted at room temperature, where thermal fluctuations generally reduce the effective DMI [*Phys. Rev. B* **101**, 054433 (2020); *Appl. Phys. Lett.* **113**, 092402 (2018)]. Second, the experimentally extracted DMI represents a spatially averaged value over the sample, which can be reduced by inhomogeneous Pd distribution in real materials.

In the revised manuscript, the relevant data and discussions have been added into the revised Supplementary Information (Supplementary **Fig. S16** and **Tables S5, S6**).

**Comment #6:** What are the calculated magnetic moments of Fe atoms in $Fe_3GaTe_2$? Are they consistent with experimental results? As far as I know, the GGA functional in DFT calculations will overestimate the magnetic moments of Fe atoms obviously, so LDA potential should be more reasonable used to study the magnetic properties of $Fe_3GaTe_2$.

**Authors' Reply:** We sincerely appreciate the reviewer's insightful comments regarding

the accuracy of calculated Fe magnetic moments and the choice of exchange-correlation functional. In the previous version of our manuscript, the magnetic moments of Fe atoms were calculated using the GGA functional. For $Fe_{2.88}GaTe_2$, the magnetic moments of $Fe_1$ ($M_{Fe1}$) and $Fe_2$ ($M_{Fe2}$) were 2.224 $\mu_B$ and 1.459 $\mu_B$, respectively, yielding an average magnetic moment ($M_{ave}$) of 1.974 $\mu_B$. For $Fe_{2.88}GaTe_2Pd_{0.05}$, the corresponding values are 2.211 $\mu_B$ and 1.443 $\mu_B$, with an average of 1.960 $\mu_B$ per Fe atom (**Table R5**). These results indicate that Pd intercalation slightly reduces the magnetic moment of Fe atoms.

**Table R5.** Calculated magnetic moments for $Fe_1$ ($M_{Fe1}$), $Fe_2$ ($M_{Fe2}$), and average magnetic moments ($M_{ave}$) in $Fe_{2.88}GaTe_2$ and $Fe_{2.88}GaTe_2Pd_{0.05}$ using GGA, GGA+U, LDA, and LDA+U functionals.

| Functionals | Structure | $M_{Fe1}$($\mu_B$) | $M_{Fe2}$($\mu_B$) | $M_{ave}$ ($\mu_B$) |
|---|---|---|---|---|
| GGA | $Fe_{2.88}GaTe_2$ | 2.224 | 1.459 | 1.974 |
| | $Fe_{2.88}GaTe_2Pd_{0.05}$ | 2.211 | 1.443 | 1.960 |
| LDA | $Fe_{2.88}GaTe_2$ | 2.049 | 1.329 | 1.814 |
| | $Fe_{2.88}GaTe_2Pd_{0.05}$ | 2.031 | 1.320 | 1.798 |
| GGA + U (U = 2.5eV) | $Fe_{2.88}GaTe_2$ | 2.841 | 2.158 | 2.618 |
| | $Fe_{2.88}GaTe_2Pd_{0.05}$ | 2.759 | 2.140 | 2.556 |
| LDA + U (U = 2.5eV) | $Fe_{2.88}GaTe_2$ | 2.662 | 1.952 | 2.430 |
| | $Fe_{2.88}GaTe_2Pd_{0.05}$ | 2.589 | 1.909 | 2.367 |

Following the reviewer's comments, we performed calculations using the LDA functional (**Table R5**). Within LDA, $M_{Fe1}$ and $M_{Fe2}$ are reduced to 2.049 $\mu_B$ and 1.329 $\mu_B$ in $Fe_{2.88}GaTe_2$, and to 2.031 $\mu_B$ and 1.320 $\mu_B$ in $Fe_{2.88}GaTe_2Pd_{0.05}$, respectively. Compared with GGA, LDA yields $M_{ave}$ that are lower by approximately ~0.16 $\mu_B$ for $Fe_{2.88}GaTe_2$ and ~0.17 $\mu_B$ for $Fe_{2.88}GaTe_2Pd_{0.05}$. We also performed calculations using the GGA + U (U = 2.5 eV) and LDA + U (U = 2.5 eV) functionals. In contrast to LDA + U, $M_{ave}$ calculated by GGA + U in $Fe_{2.88}GaTe_2$ and $Fe_{2.88}GaTe_2Pd_{0.05}$ are overestimated by ~0.18 $\mu_B$ and ~0.19 $\mu_B$, respectively.

Overall, these results indicate that the difference between LDA and GGA leads to only a slight variation in the calculated Fe magnetic moments. Importantly, both computational approaches consistently reproduce the relative trend that Pd intercalation reduces the Fe magnetic moments, in agreement with the experimentally observed suppression of saturation magnetization.

**Comment #7:** The considered Fe vacancies are distributed in two different $Fe_3GaTe_2$

layers. In principle, Fe vacancies can be distributed in the same layer in practice. So does the favorable Pd doping site depend on the Fe vacancies configurations in theoretical calculations and experimental samples?

**Authors' Reply:** We sincerely appreciate the reviewer's insightful comments, which are of great significance to improve the quality of our manuscript. Previous studies have identified the coexistence of $Fe_1$ and $Fe_2$ vacancies in $Fe_{3-\delta}GeTe_2$, which induces a displacement of $Fe_2$ atoms along the *c*-axis [*Nat. Commun.* **15**, 1017 (2024)]. Such $Fe_2$-sites deviations break the centro-symmetry and gives rise to a pronounced DMI. To establish the energetically favorable Pd intercalation site, we constructed a 3×3 supercell containing two Fe vacancies and one Pd dopant, corresponding to a composition of $Fe_{2.88}GaTe_2Pd_{0.05}$.

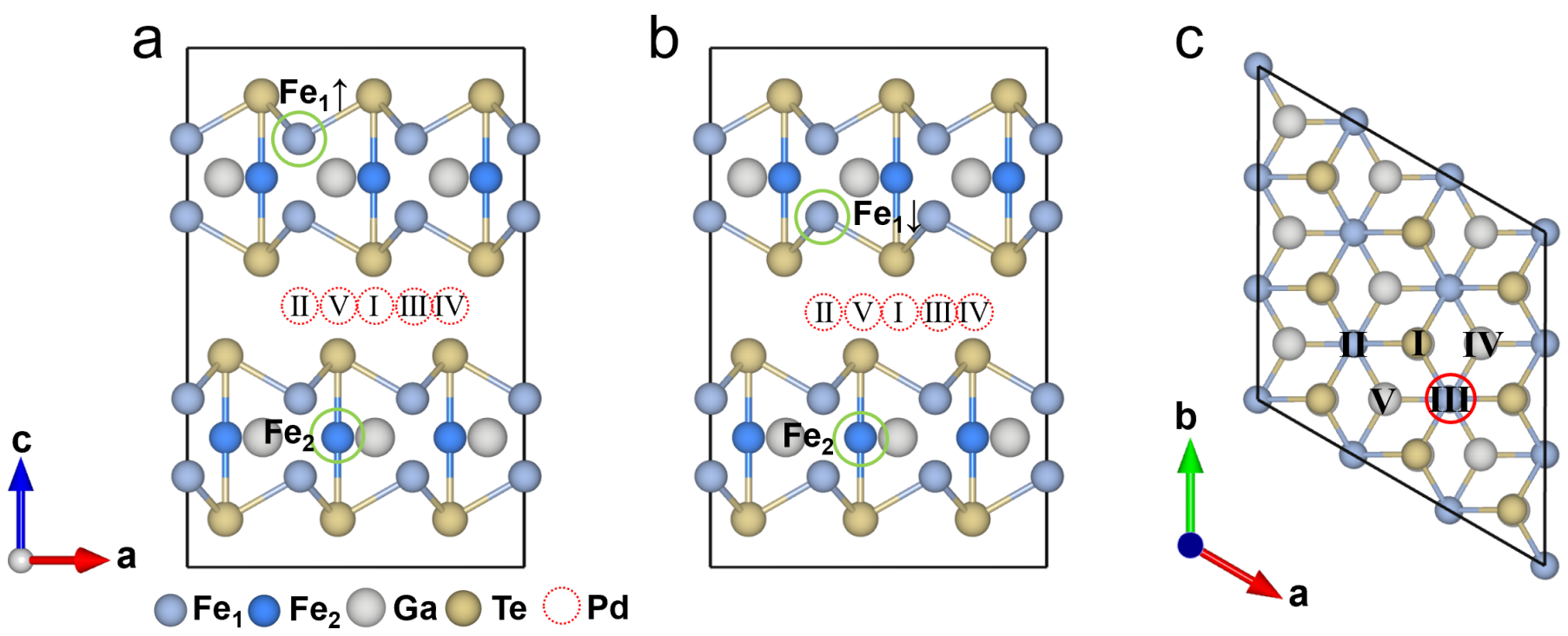


**Fig. R4. a,** $Fe_1$↑(upper) + $Fe_2$(lower), in which $Fe_1$ is located in the upper layer farthest from the vdW gap and $Fe_2$ in the lower layer. **b,** $Fe_1$↓(upper) + $Fe_2$(lower), where $Fe_1$ is located in the upper layer closest to the vdW gap and $Fe_2$ remains in the lower layer. **c,** Five Pd candidate positions (labeled as I, II, III, IV, and V).

In the previous version of our manuscript, we focused on vacancy configurations in which Fe vacancies are distributed across two different layers, i.e., (i) $Fe_1$↑(upper) + $Fe_2$(lower), where $Fe_1$↑(upper) vacancy is located in the upper layer farther from the vdW gap and $Fe_2$ (lower) is located in the lower layer (**Fig. R4a**) and (ii) $Fe_1$↓(upper) + $Fe_2$(lower), where $Fe_1$↓(upper) vacancy is located in the upper layer closer to the vdW gap and $Fe_2$ remains in the lower layer (**Fig. R4b**). For each vacancy configuration, five possible Pd intercalation sites (denoted as I, II, III, IV, and V) were examined (**Fig. R4c**), and all structures were fully relaxed to their energy minima. We systematically analyzed how the vacancy configurations influence the deviation of $Fe_2$ atoms, the equilibrium position of Pd within the vdW gaps, and the resulting magnetic parameters. We found that only the $Fe_1$↑(upper) + $Fe_2$(lower) + $Pd_{III}$ configuration can simultaneously reproduce the experimental observations. Detailed discussions have

been presented in the Supplementary **Note 2** of our previous manuscript.

As pointed out by the reviewer, Fe vacancies could, in principle, also be distributed within the same $Fe_{3-\delta}GaTe_2$ layer. Following the reviewer's comments, we have considered the configurations in which two Fe vacancies are located within the same layer, i.e., (i) $Fe_1$↑(upper) + $Fe_2$(upper), where $Fe_1$↑(upper) is located in the upper layer farther from the vdW gap and $Fe_2$ (upper) is located in the upper layer (**Fig. R5a**), and (ii) $Fe_1$↓(upper) + $Fe_2$(upper), where $Fe_1$↓(upper) is located in the upper layer closer to the vdW gap and $Fe_2$(upper) is located in the upper layer (**Fig. R5b**). For each vacancy configuration, we also examined five possible Pd intercalation sites (denoted as I, II, III, IV, and V) (**Fig. R5c**). However, as discussed below, these configurations do not yield results consistent with experimental observations.

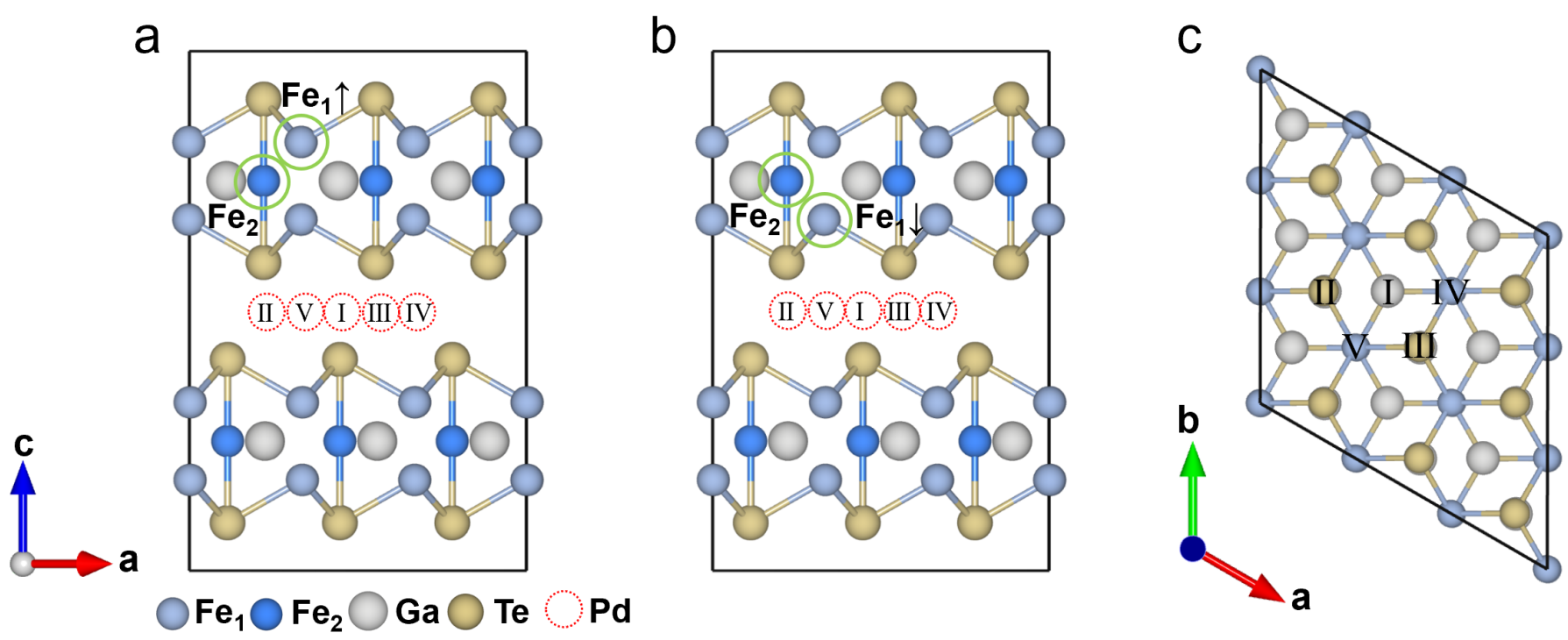


**Fig. R5. a,** $Fe_1$↑(upper) + $Fe_2$(upper), in which $Fe_1$ is located in the upper layer farthest from the vdW gap and $Fe_2$ in the upper layer. **b,** $Fe_1$↓(upper) + $Fe_2$(upper), where $Fe_1$ is located in the upper layer closest to the vdW gap and $Fe_2$ remains in the upper layer. **c,** Five Pd candidate positions (labeled as I, II, III, IV, and V).

The optimized results show that, for both the $Fe_1$↑(upper) + $Fe_2$(upper) and $Fe_1$↓(upper) + $Fe_2$(upper) vacancy configurations, the Pd atom is stably located within the middle position of the vdW gap only when occupying site IV or V. In contrast, for site II, the Pd atom shifts towards a neighboring Te atom, while for sites I and III, it migrates into the lower Fe-containing layer rather than locating within the vdW gap. We then calculated the nearest-neighbor exchange coupling constants ($J_1$) for both $Fe_{2.88}GaTe_2$ and $Fe_{2.88}GaTe_2Pd_{0.05}$ in the $Fe_1$↑(upper) + $Fe_2$(upper) + $Pd_{IV}$, $Fe_1$↓(upper) + $Fe_2$(upper) + $Pd_{IV}$, $Fe_1$↑(upper) + $Fe_2$(upper) + $Pd_V$, and $Fe_1$↓(upper) + $Fe_2$(upper) + $Pd_V$ configurations. As summarized in **Table R6**, Pd intercalation in these configurations leads to an enhancement of $J_1$, which is in contradiction with the experimentally observed reduction of exchange coupling.

Based on these results, we conclude that the energetically favorable Pd intercalation

site is sensitive to the Fe-vacancy configuration, and only vacancy configurations in which Fe vacancies are distributed across different layers reproduce the experimentally observed Pd position and magnetic trends. The corresponding discussions have been added in Supplementary **Fig. S7**.

**Table R6.** Calculated nearest-neighbor exchange coupling constants ($J_1$) for both $Fe_{2.88}GaTe_2$ and $Fe_{2.88}GaTe_2Pd_{0.05}$ in the $Fe_1$↑(upper) + $Fe_2$(upper) + $Pd_{IV}$, $Fe_1$↓(upper) + $Fe_2$(upper) + $Pd_{IV}$, $Fe_1$↑(upper) + $Fe_2$(upper) + $Pd_V$, and $Fe_1$↓(upper) + $Fe_2$(upper) + $Pd_V$ configurations.

| Vacancy configuration | Pd site | Structure | $J_1$ (meV) |
|---|---|---|---|
| | | $Fe_{2.88}GaTe_2$ | 27.541 |
| $Fe_1$↑(upper) + $Fe_2$ (upper) | IV | $Fe_{2.88}GaTe_2Pd_{0.05}$ | 29.200 |
| | V | $Fe_{2.88}GaTe_2Pd_{0.05}$ | 27.815 |
| | | $Fe_{2.88}GaTe_2$ | 27.541 |
| $Fe_1$↓(upper) + $Fe_2$ (upper) | IV | $Fe_{2.88}GaTe_2Pd_{0.05}$ | 29.097 |
| | V | $Fe_{2.88}GaTe_2Pd_{0.05}$ | 28.621 |

**Comment #8:** The theoretical results play an important to obtain the experimental observations in this work, so some important details for micromagnetic simulations and first-principe calculations should be given in the "Methods" part in the maintext.

**Authors' Reply:** We sincerely appreciate the reviewer's insightful comments. We have added important details for micromagnetic simulations and first-principle calculations into the "Methods" section in the revised manuscript.

"*Micromagnetic simulation*"

Micromagnetic simulations were performed using the GPU-accelerated simulation package MuMax[3]. The simulated geometry was a slab with dimensions of 512 × 512 × 64 $nm^3$ and a mesh size of 2.0 × 2.0 × 2.0 $nm^3$. Periodic boundary conditions are applied to emulate large-scale simulations. The total energy of the system includes contributions from exchange interaction, first-order uniaxial magnetic anisotropy, Dzyaloshinskii-Moriya interaction (DMI), demagnetization energy, and Zeeman energy. The initial magnetization process is simulated using magnetic parameters guided by experimental measurements, including ferromagnetic exchange constant ($J$), effective magnetic anisotropy constant ($K_{eff}$), DMI constant ($D$), and saturation magnetization ($M_s$). The specific values used are: $J$ = 1.1 pJ/m; $K_{eff}$ = 1.5 × $10^5$ J/$m^3$; $D$ = 0.25 mJ/$m^2$; $M_s$= 2.5 × $10^5$ A/m. Gilbert damping ($\alpha$) is set to be 0.1, following the experimentally reported value of 0.075 for $Fe_3GaTe_2$ at 300 K.

*“First-principle calculations”*

Based on density functional theory (DFT), we employed the Vienna ab initio simulation package (VASP) to calculate the forces, electronic structures, and magnetic properties of the non-stoichiometric $Fe_{3-\delta}GaTe_2$ and Pd-intercalated $Fe_{3-\delta}GaTe_2$ (that is, $Fe_{3-\delta}GaTe_2Pd_x$). The exchange-correlation interaction was described by the Perdew-Burke-Ernzerhof (PBE) functional under the generalized gradient approximation (GGA). The strong correlation between the localized *d* orbitals was taken into account by including a Hubbard U term through the GGA + U formulation [*Phys. Rev. B* **74**, 125106 (2006)]. An effective U value of 2.5 eV was applied. The interaction between valence electrons and ionic cores was described within the framework of the projector augmented wave (PAW) method. The energy cutoff of the plane-wave basis was set to 500 eV, and the Brillouin Zone was sampled by Γ-centered k-point mesh of 0.02 $Å^{-1}$. During geometry optimization, both lattice constants and atomic positions were fully relaxed until the force on each atom was less than 0.01 eV/Å and the energy convergence criterion was less than $10^{-6}$ eV. Moreover, vdW interactions were included using the DFT-D3 in all calculations.

**Comment #9:** “first-principal” should be “first-principle”.

**Authors’ Reply:** We sincerely appreciate the reviewer’s insightful comments. We have corrected “first-principal” to “first-principle” throughout the manuscript.

**Response to the Report of Reviewer A**

**Reviewer A's general Comment:** I appreciate the authors' sincere response to my comments. While most concerns have been addressed, I find that the reply to Comment 3 is still insufficient and may contain inaccuracies.

**Authors' Reply:** We sincerely thank the reviewer for the careful reading of our manuscript and for pointing out the remaining concerns regarding Comment 3. In the revised manuscript, we have revised the descriptions of the VGG network and provided additional technical details to ensure accuracy. We hope the reviewer will be satisfied with the revised manuscript as well as our responses.

**Reviewer A's Comment #1:** In the revised manuscript, the explanation of the connection between experiment and simulation has become clearer, in which a new quantity "feature array X" is introduced. However, at least in the present explanation, it remains unclear how this feature array is obtained. I assume that the authors use the VGG network provided in Fig. 5c of the initial manuscript (although its structure has been removed in the revised version). However, according to Fig. 5c in the revised manuscript, the dimension of the feature matrix is n and is the same as that of the output probability. If my understanding is correct, n = 10 in this case, which differs from the dimension of the final feature layer of the VGG network (layer 5), namely 512. This point is confusing, and I recommend providing a more detailed explanation and/or correcting possible mistakes.

**Authors' Reply:** We sincerely thank the reviewer for carefully examining the dimensional consistency between the feature array $X$ and the output probability vector $Y$. This insightful comment has helped us identify and correct an important inconsistency in the previous version of the manuscript. In our work, the dimension of the feature array $X$ is 512, whereas the output probability vector $Y$ has a dimension of 10. We regret that **Fig. 5c** in the previous revised manuscript incorrectly indicated identical dimensions for the two quantities, which may have caused confusion. This error has now been corrected in the updated figure. Following the reviewer's suggestion, we have also provided a more detailed description clarifying how the feature array $X$ is obtained, as described below.

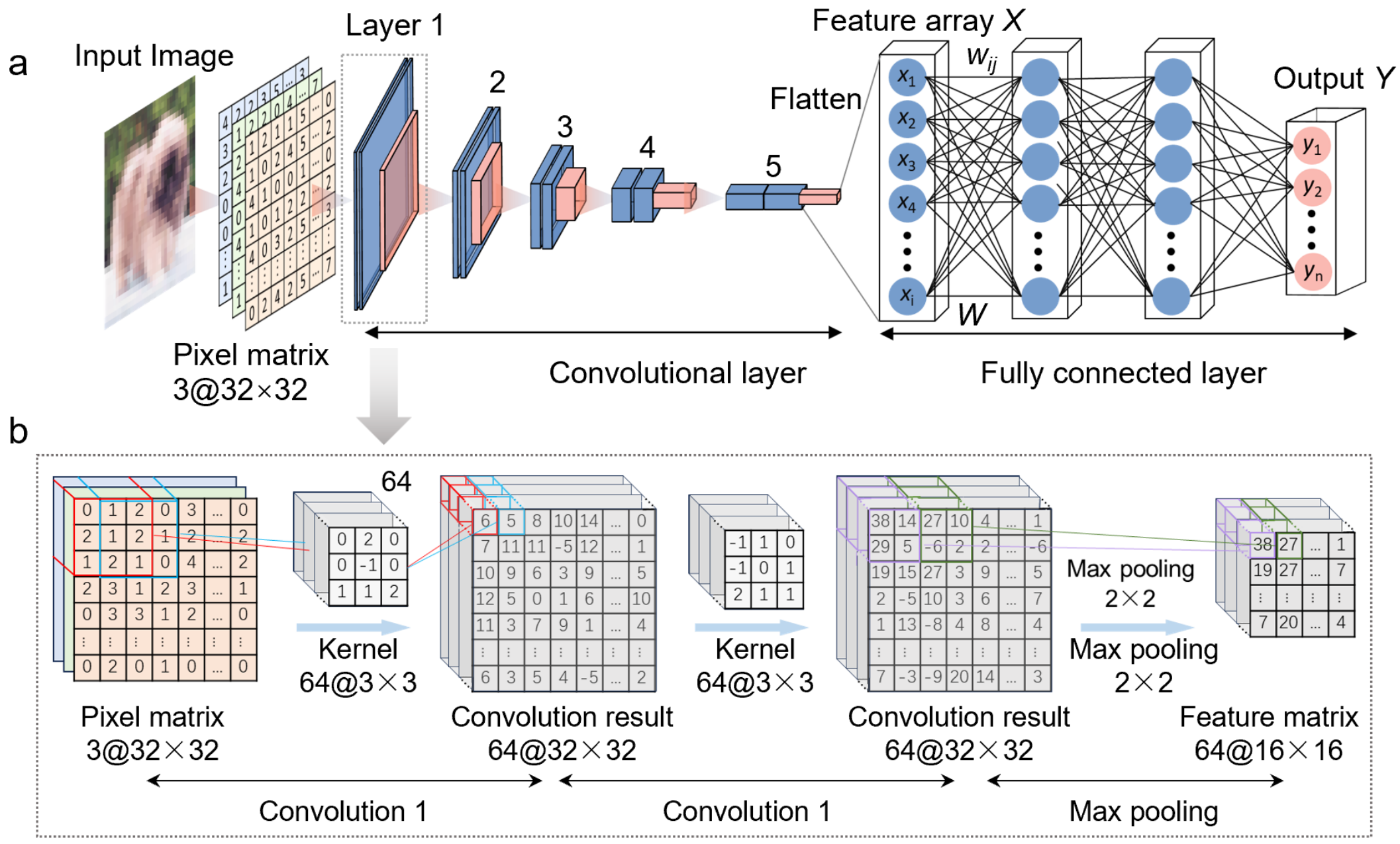


**Fig. R1. Network structure of the Visual Geometry Group (VGG) network-16. a,** Overall structure of the network, consisting of five convolutional blocks (layers 1-5) followed by fully connected layers (illustrated as cuboids with blue and red circles). **b,** Detailed illustration of the convolution and max-pooling operations in convolutional layer 1. The input is a 32×32-pixel matrix with three RGB channels (red, green and blue). After convolution with 64 kernels of size 3×3, 64 feature maps of size 32×32 (3@32×32) are generated. After two consecutive convolutions, a max pooling operation is applied, leading to 64 feature maps of size 16×16 (64@16×16). The 3@3×3 red boxes in the pixel matrix and 64@1×1 red boxes in convolution results indicate the data before and after the convolution operations, respectively. The 3@3×3 blue boxes in the pixel matrix and 64@1×1 blue boxes in convolution results represent the data before and after convolution after a horizontal shift of one step. In the max pooling process, the purple boxes highlight the data before and after the max pooling operation, where each 2×2 region is reduced to 1×1. The green boxes represent the pooling operation after two horizontal shifts.

The complete convolutional neural network architecture used in this work is schematically illustrated in **Fig. R1a**. In this framework, the input image is represented as a pixel matrix with a dimension of 3@32×32, where 3 represents the number of image channels and 32×32 corresponds to the spatial resolution. This input matrix is progressively transformed into a feature matrix through a sequence of convolution (blue boxes) and max-pooling (red boxes) operations across layers 1-5. Taking convolutional layer 1 as an example, it comprises two consecutive convolution operations followed by a max pooling operation (**Fig. R1b**). Firstly, the pixel matrix is convolved with the convolution kernels, each having a size of 3×3. During convolution, the kernel element is multiplied by the corresponding element in the 3×3 region of pixel matrix across all three image channels (marked by the red box in the pixel matrix), and the results are summed to produce a single output feature value (marked by the red box in the convolution result). The 3×3 region of pixel matrix then slides horizontally with a stride

of 1 (marked by the blue box in the pixel matrix), and this process is repeated across all spatial positions, forming the first convolution layer. Given that there are 64 convolution kernels, the resulting convolution output has a dimension of 64. After two such convolution operations, a 2×2 max pooling operation with stride 2 is applied. In this process, the spatial resolution is reduced while retaining the maximum value, resulting in a feature matrix with dimension of 64@16×16.

The feature matrix is then processed by layers 2-5, with progressively increased channel depth and reduced spatial size. The resulting feature matrix dimensions are: 64@16×16 (layer 1), 128@8×8 (layer 2), 256@ 4×4 (layer 3), 512@2×2 (layer 4), and 512@1×1 (layer 5). The final 512@1×1 feature matrix is flattened into a one-dimensional array containing 512 elements, which is defined as the feature array *X*. This feature array is then fed into the fully connected layer to perform classification, producing the output probability vector *Y* with a dimension of 10.

In the revised manuscript, **Fig. 5b, 5c** in the last version of our manuscript is replaced by **Fig. R1a**; Simultaneously **Fig. R1b** and associated descriptions have been added into the Supplementary Information (Supplementary **Note 4** and **Fig. S28**).

**Reviewer A's Comment #2:** In addition, in any case, weights are required to obtain the feature array X. From the revised explanation of the simulation, it appears that the skyrmion device is used only in the final layer, namely in the calculation of Y from X. Thus, it is unclear what advantage is gained by using the skyrmion device only in the final layer. For example, the VGG network contains more than one million parameters. Thus, if the authors use the VGG network to calculate X, the number of parameters in the final layer is much smaller compared with the total number of parameters. To emphasize the high accuracy of the skyrmion device, should the authors perform simulations in which the skyrmion device is used for all layers? Before publication, these points should be clarified.

**Authors' Reply:** We sincerely thank the reviewer for the valuable comments, which have helped us further improve the clarity and quality of the manuscript. Actually, the fully connected neural network in our work contains three layers, as schematically illustrated in **Fig. R2**. The feature array *X* is weighted and summed through three weight

matrixes to obtain the output $Y$. Although the complete VGG network contains more than one million parameters, the three fully connected layers employed in our study already comprise $5.29 \times 10^5$ parameters, accounting for a considerable proportion of the total network parameters. Moreover, the convolutional layers mainly focus on image feature extraction from input images. In contrast, the fully connected layers directly determine the final classification probabilities and thus play a more critical role in the network. Therefore, implementing skyrmion devices in the fully connected layers already covers a significant fraction of the network parameters and effectively highlights the advantages of the hardware realization of skyrmion devices for neural network. To avoid confusion, the **Fig. R2** and related descriptions have been added into the Supplementary Information (Supplementary **Note 4** and Supplementary **Fig. S29**).

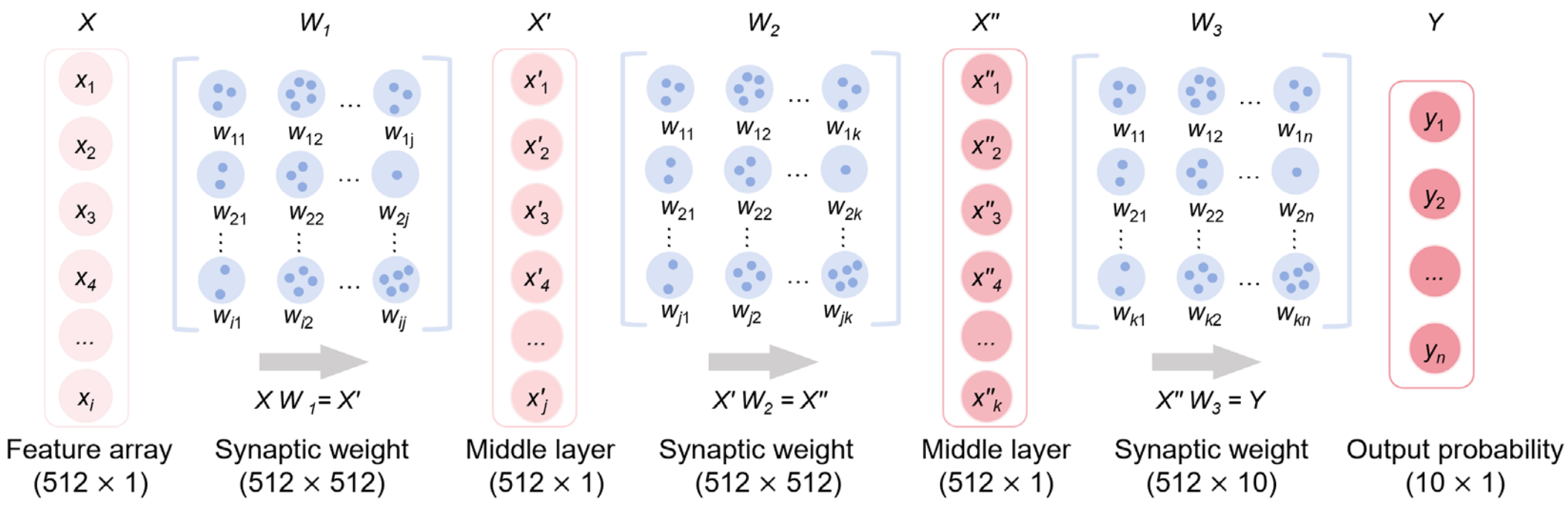


**Fig. R2. Schematic illustration of the fully connected layers.** The input 512-dimensional feature array $X$ is multiplied by the weight matrix $W_1$ to obtain the intermediate representation $X'$, which is then multiplied by $W_2$ to produce $X''$, and finally multiplied by $W_3$ to generate the output $Y$. The numbers shown at the bottom of the figure indicate the data dimensions at each layer as well as the sizes of the corresponding weight matrices.

**Response to the Report of Reviewer B**

**Reviewer B's Comment #1:** I remain unconvinced by the authors' rebuttal that the present work meets the novelty threshold required for publication in Nature Communications.

The core outcome of this manuscript remains an incremental advance rather than a fundamentally new physical insight. As already noted in my earlier report, a closely related study by one of the corresponding authors has recently been published in Nature Communications [Nat. Commun. 15, 1017 (2024)]. The current manuscript primarily reports a reduction in skyrmion size and an increase in skyrmion density upon Pd intercalation, which—given the existing literature—are anticipated consequences of modifying exchange interactions and DMI via heavy-element intercalation.

**Authors' Reply:** We sincerely thank the reviewer for the careful reading of our manuscript and for raising the important concern regarding novelty. While we understand the reviewer's concern, we believe that the present work extends beyond an incremental advance. The previous work [*Nat. Commun.* 15, 1017 (2024)] focused primarily on demonstrating room-temperature skyrmions in $Fe_{3-\delta}GaTe_2$ and clarifying the microscopic origin of DMI in that system. In contrast, the objective of the present study is to realize optically activated skyrmion neuromorphic devices through an integrated materials-to-control strategy. The scientific questions addressed here therefore shift from "whether skyrmions exist and how DMI arises" to "how to engineer a vdW magnetic system to enable ultrafast, low-energy, multi-level skyrmion manipulation suitable for device functionality."

Achieving this objective requires overcoming two critical challenges: First, skyrmions in existing vdW magnets typically exhibit diameters of ~100 nm at room temperature, limiting integration density and scalability. Substantially reducing their size while maintaining high density is essential for device-level implementation. Second, laser-induced deterministic multi-level modulation of skyrmions, a critical requirement for performing neuromorphic computing, has yet to be achieved.

In this work, we successfully address both challenges by employing an integrated materials-to-control strategy:

**(i) Skyrmion size miniaturization via Pd intercalation.** Guided by systematic micromagnetic simulations, we established a material-design strategy centered on enhancing the Dzyaloshinskii-Moriya interaction while suppressing Heisenberg

exchange coupling to achieve both skyrmion miniaturization and high density in the vdW magnets. Implementing this Pd atomic intercalation into the vdW magnet $Fe_{3-\delta}GaTe_2$, we achieved magnetic-field-stabilized densely packed skyrmions with a minimum average diameter of ~43 nm at room temperature, representing the smallest skyrmions reported to date in vdW magnets.

**(ii) Ultrafast, multi-level modulation of sub-50 nm skyrmions.** Leveraging this tailored vdW material platform and a custom-built in situ Lorentz transmission electron microscopysetup with fs laser excitation, we demonstrate fs laser-induced ultrafast generation of ~43 nm skyrmions, with a low energy consumption of only 0.6 pJ per skyrmion, which is over an order of magnitude lower than that required for the current-driven skyrmion control. Most importantly, we achieve pulse-number-dependent, multi-level modulation of skyrmion density, enabling skyrmion-based optical neuromorphic computing with a training accuracy of ~92%.

We believe this work will be of great interest to the broad readers of *Nature Communications* because these results open promising opportunities for high-speed, energy-efficient, and brain-inspired spintronic devices.

**Reviewer B's Comment #2:** The authors emphasize "vdW gap engineering" as a key novelty. While I agree that intercalation engineering is an important approach, this concept is already well established in Fe-based vdW magnets. For example, transition-metal intercalation (such as Co in $Fe_5GeTe_2$) and self-intercalation effects are widely known to alter symmetry, magnetic anisotropy, and DMI.

**Authors' Reply:** We fully agree that intercalation engineering is a well-established strategy in Fe-based vdW magnets. However, the advance of the present work does not lie in introducing intercalation as a concept, but in demonstrating how vdW-gap engineering can be employed to drive the system into a fundamentally different magnetic interaction regime with distinct dynamical consequences.

In the present study, Pd intercalation does not merely enhance spin-orbit coupling. Instead, it simultaneously enhances DMI while reducing the Heisenberg exchange interaction at $Fe_2$-$Fe_2$ bonds, thereby modifying the ratio of the exchange constant ($J$) and DMI constant ($D$), i.e., $J/D$. This cooperative modification of magnetic interactions enables the system to enter a low $J/D$ regime that is not typically realized in previously reported Fe-based vdW magnets. Accessing this interaction regime leads to three experimentally demonstrated consequences:

(i) Stabilization of densely packed skyrmions with a minimum average diameter of ~43 nm in Pd-intercalated $Fe_{3-\delta}GaTe_2$ at room temperature, which significantly improves integration density compared to the ~100 nm scale commonly observed in vdW magnets.

(ii) Femtosecond-laser-induced ultrafast generation of ~43 nm skyrmions at a density of ~101 $\mu m^{-2}$, with an energy consumption of only 0.6 pJ per skyrmion, which is approximately one-third of that in pristine $Fe_{3-\delta}GaTe_2$ and over an order of magnitude lower than that required for current-driven control.

(iii) Deterministic, pulse-number-dependent multi-level modulation of skyrmion density, enabling skyrmion-based optical neuromorphic computing with a training accuracy of ~92%.

To the best of our knowledge, this is the first experimental study demonstrating that heavy-metal intercalation can be employed as a vdW-gap engineering strategy to access a new magnetic interaction regime in Fe-based vdW magnets. Therefore, the novelty does not arise from intercalation as a chemical tool, but from the discovery that vdW-gap engineering can access a distinct magnetic regime enabling energy-efficient neuromorphic functionality.

**Reviewer B's Comment #3:** In $Fe_{3-\delta}GaTe_2$ specifically, symmetry breaking and the stabilization of Néel-type skyrmions are fundamentally linked to Fe deficiency and Fe self-intercalation in the vdW gaps. Without such intercalation, the parent compound should not host Néel-type skyrmions.

**Authors' Reply:** We thank the reviewer for raising this important point regarding the structural origin of symmetry breaking and Néel-type skyrmions in the Fe deficiency $Fe_{3-\delta}GaTe_2$. We would like to respectfully clarify that Fe deficiency and Fe self-intercalation in the vdW gap correspond to two distinct structural scenarios that should not be conflated.

In previously reported Fe-self-intercalated $Fe_3GaTe_2$ (i.e., $Fe_{3+\delta}GaTe_2$), excess Fe atoms occupy sites within the vdW gaps, which modifies inversion symmetry and magnetic interactions [*Nat. Commun.* 15, 3828 (2024)]. In contrast, for Fe-deficient $Fe_3GaTe_2$ (i.e., $Fe_{3-\delta}GaTe_2$), it has been well established that the coexistence of $Fe_1$ and $Fe_2$ vacancies induces a displacement of $Fe_2$ atoms along the *c*-axis [*Nat. Commun.* 15, 1017 (2024); *Nat. Commun.* 15, 3278 (2024); *Adv. Mater.* 36, 2311022 (2024)]. This vacancy-induced lattice distortion breaks local centrosymmetry and gives rise to a

pronounced DMI. Importantly, in both our previous work and reports from other groups, single-crystal X-ray diffraction (XRD) [*Nat. Commun.* 15, 1017 (2024)] and atomic-resolution transmission electron microscopy (TEM) [*Nat. Commun.* 15, 1017 (2024); *Nat. Commun.* 15, 3278 (2024); *Adv. Mater.* 36, 2311022 (2024)] consistently show no Fe occupation in the vdW gap of Fe-deficient $Fe_{3-\delta}GaTe_2$. Therefore, although Fe intercalation is absent in the vdW gap, inversion symmetry breaking, DMI, and Néel-type skyrmions can still emerge in the Fe-deficient $Fe_{3-\delta}GaTe_2$.

**Reviewer B's Comment #4:** In this context, the authors' strong claim—based on STEM/EELS—that no Fe exists in the vdW gaps and that only Pd is present is difficult to accept unambiguously. Given the presence of heavy Pd atoms, the absence of detectable Fe signal in STEM/EELS does not conclusively rule out Fe self-intercalation, as Fe contrast can be strongly suppressed. Therefore, it remains challenging to disentangle whether the observed modification of DMI arises exclusively from Pd intercalation, or whether intrinsic self-intercalation effects also contribute. As a result, the assertion that Pd intercalation alone creates a "new material platform" is not sufficiently justified.

**Authors' Reply:** We thank the reviewer for raising the concern regarding the potential self-intercalation of Fe atoms. To address this concern, we provide a justification based on both crystal growth mechanisms and atomic-scale characterization.

First, from the perspective of crystal growth, previous studies on $Fe_3GaTe_2$ have established that Fe self-intercalation occurs under Fe-rich growth conditions [*Nat. Commun.* 15, 3828 (2024)], where excess Fe atoms occupy vdW-gap sites. In contrast, the present work employs Fe-deficient $Fe_3GaTe_2$ (i.e., $Fe_{3-\delta}GaTe_2$) as the precursor. Under such Fe-deficient conditions, there is no thermodynamic driving force for additional Fe atoms to occupy vdW-gap sites. This growth strategy substantially reduces the possibility of intrinsic Fe self-intercalation prior to Pd insertion. To confirm this result from thermodynamics, we have further calculated the formation energy ($E_f$) of Fe- and Pd-intercalated $Fe_{2.88}GaTe_2$, as schematically illustrated in **Fig. R1**. The formation energy is defined as:

$$E_f = E_{Intercalated\text{-}FGT} - (E_{FGT} + E_{Intercalated\text{-}atom}), \quad (1)$$

where $E_{Intercalated\text{-}FGT}$ is the total energy of the intercalated system, $E_{FGT}$ is the total energy of pristine $Fe_{2.88}GaTe_2$, and $E_{Intercalated\text{-}atom}$ is the energy of the isolated intercalated atom. The calculated formation energies are -5.157 eV/cell for Pd

intercalation ($E_{Intercalated-Pd}$) and -4.383 eV/cell for Fe intercalation ($E_{Intercalated-Fe}$), indicating that Pd is energetically more favorable for occupying the vdW gaps of $Fe_{2.88}GaTe_2$. These results theoretically confirm that the intercalated atoms in $Fe_{3-\delta}GaTe_2Pd_{0.1}$ are Pd atoms rather than Fe atoms.

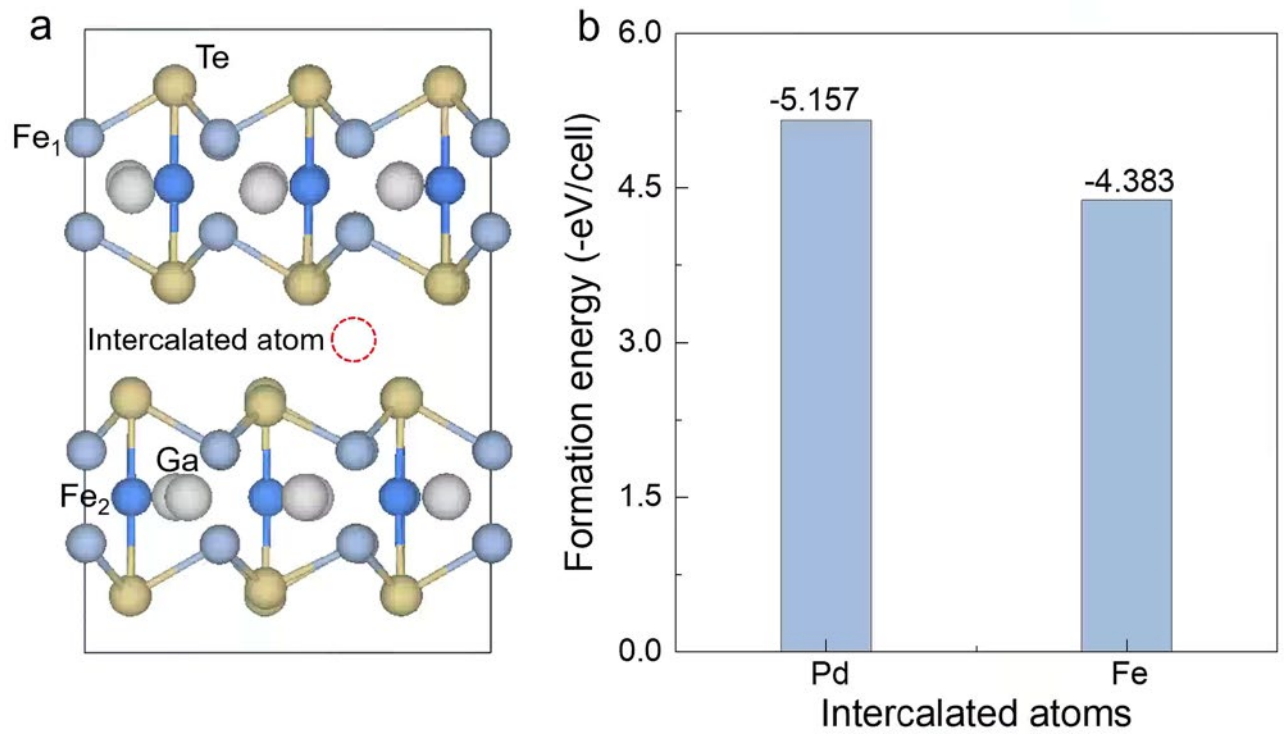


**Fig. R1. a,** Structure configuration of intercalated atoms in the gaps of the $Fe_{2.88}GaTe_2$. **b,** The calculated formation energies of Fe- and Pd-intercalated gaps of the $Fe_{2.88}GaTe_2$.

Second, regarding the reviewer's concern about signal masking in STEM images, we fully agree with this professional judgment. In Z-contrast HAADF-STEM imaging, the scattering cross-section of heavy Pd atoms (Z = 46) is significantly larger than that of lighter Fe atoms (Z = 26), meaning the strong signal from Pd can obscure the weak contrast of Fe in imaging mode. To overcome this limitation, we performed atomically resolved EELS and EDX analyses specifically targeting the vdW-gap regions.

It is important to emphasize that the physical mechanism of EELS elemental identification is based on inner-shell electron ionization, which differs fundamentally from the elastic scattering used in STEM imaging. In EELS spectroscopy, elemental identification relies on characteristic ionization edges that are strictly separated along the energy axis [*Ultramicroscopy* 34, 165 (1990); *Micron* 28, 333 (1997)], meaning the brightness suppression effect observed in STEM imaging does not apply here. Specifically, the Fe $L_{2,3}$-edge at ~708 eV is energetically well separated from the Pd $M_1$-edge at ~670 eV. More importantly, as a 3d transition metal, the Fe L-edge exhibits a high ionization cross-section and extremely sharp white line features. This implies that even in the presence of a heavy atom background, the distinct spectral signature of Fe would be easily detectable if even trace amounts were present in the vdW gaps. However, as shown in the **Fig. R2**, our EELS scans focused on the vdW gap regions of the Pd-intercalated samples clearly reveal the Pd signal, while the spectrum within the Fe energy window appears as a flat baseline with absolutely no distinguishable Fe characteristic peaks. Simultaneous EDX analysis (**Fig. 2b** in the main text) further

corroborates this finding by confirming the presence of Pd and the absence of Fe within the interlayers.

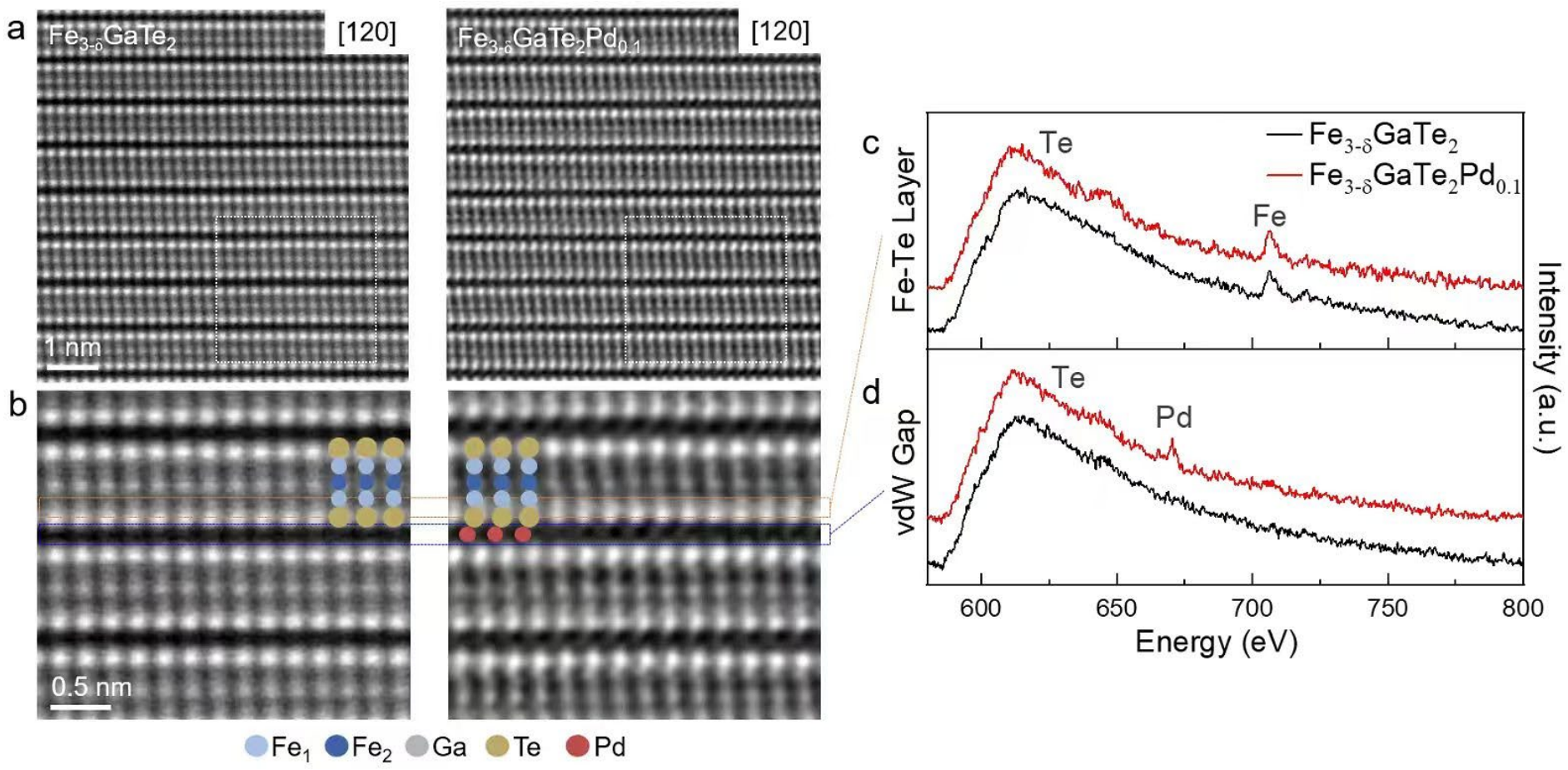


**Fig. R2.** Atomic images and EELS spectrum of $Fe_{3-\delta}GaTe_2$ and $Fe_{3-\delta}GaTe_2Pd_{0.1}$. **a**, Annular bright-field (ABF) image of taken along the [120] zone axis. Scale bars represent 1 nm. **b**, Enlarged atomic images corresponding to the regions marked in panel (a). The elements are represented as follows: $Fe_1$ (light blue), $Fe_2$ (dark blue), Te (yellow), Pd (red). Scale bars represent 0.5 nm. **c**, **d** EELS spectrum obtained from different layers, as indicated by the square boxs marked in panel **b**.

In summary, this dual evidence based on the synthesis control using Fe-deficient precursors and the data from high-sensitivity EELS and EDX spectroscopy allows us to decisively rule out the possibility of Fe self-intercalation. Therefore, we are confident that this system represents a new material platform dominated by pure Pd intercalation, and the DMI modulation is attributed to the introduction of Pd atoms.

**Reviewer B's Comment #5:** The observed reduction in skyrmion size and the enhanced density—particularly under varying laser fluence are expected trends within the established framework of skyrmion physics, where heavy elements are routinely employed to tune interfacial or bulk DMI in thin films and layered magnets.

**Authors' Reply:** We thank the reviewer for this insightful comment. We agree that introducing heavy elements to tune DMI is an established strategy in skyrmion systems, and a reduction in skyrmion size may appear consistent with this general framework. However, realizing such a strategy experimentally in layered vdW magnets is far from straightforward. Successful intercalation requires chemical compatibility with the host lattice, structural stability of the vdW gaps, and preservation of long-range magnetic order. In our study, we systematically explored ten candidate heavy-metal intercalants, including Zr, Hf, Nb, Ta, Mo, W, Ir, Pt, Rh, Pd. In practice, most attempted elements either failed to enter the vdW gaps, led to structural instability, or disrupted the magnetic phase. Remarkably, Pd was the only element that could be reliably

intercalated while maintaining crystallographic integrity and ferromagnetic ordering. To the best of our knowledge, this is the first experimental study demonstrating that heavy-metal intercalation can be employed as a vdW-gap engineering strategy to access a new magnetic interaction regime in Fe-based vdW magnets. Therefore, while DMI enhancement by heavy elements may appear conceptually expected, the experimental realization of stable heavy-metal vdW-gap engineering in $Fe_{3-\delta}GaTe_2$ is highly nontrivial and required careful materials design.

On the other hand, the key advance of the present work does not lie in a simple enhancement of DMI. Pd intercalation simultaneously strengthens spin-orbit coupling and weakens the Heisenberg exchange interaction, effectively driving the system into a low $J/D$ regime. Accessing this interaction regime leads to three experimentally demonstrated consequences:

(i) Stabilization of densely packed skyrmions with a minimum average diameter of ~43 nm in Pd-intercalated $Fe_{3-\delta}GaTe_2$ at room temperature, which significantly improves integration density compared to the ~100 nm scale commonly observed in vdW magnets.

(ii) Femtosecond-laser-induced ultrafast generation of ~43 nm skyrmions at a density of ~101 $\mu m^{-2}$, with an energy consumption of only 0.6 pJ per skyrmion, which is approximately one-third of that in pristine $Fe_{3-\delta}GaTe_2$ and over an order of magnitude lower than that required for current-driven control.

(iii) Deterministic, pulse-number-dependent multi-level modulation of skyrmion density, enabling skyrmion-based optical neuromorphic computing with a training accuracy of ~92%.

While the qualitative trend of size reduction may be anticipated, the experimentally realized low $J/D$ regime and its associated ultrafast, low-energy, multi-level functionality have not been demonstrated in previously reported multilayer films, bulk magnets, or vdW skyrmion systems. We believe the present work extends beyond conventional DMI tuning and establishes a functional pathway towards energy-efficient neuromorphic devices.

**Reviewer B's Comment #6:** Consequently, while the work is thorough and may be suitable for a specialized journal focused on advances in 2D magnetic materials or skyrmion physics, I do not believe it demonstrates the level of conceptual novelty or fundamental advance required for publication in Nature Communications.

**Authors' Reply:** We sincerely appreciate the reviewer's assessment regarding the scope and conceptual advance of our work. Our advance is not limited to reducing skyrmion size or increasing density. Instead, by tailoring the *J*/*D* ratio through vdW-gap engineering, we access a magnetic interaction regime that enables pulse-number-dependent multi-level modulation of skyrmion density under ultrafast optical excitation. This dynamic controllability, together with the low energy consumption (0.6 pJ per skyrmion), allows us to demonstrate skyrmion-based optical neuromorphic computing with ~92% training accuracy. We believe this work extends beyond advances in 2D magnetic materials and will be of great interest to the broad readers of *Nature Communications* because it establishes a new pathway for integrating materials engineering and ultrafast optical control to achieve densely packed, sub-50 nm skyrmions with multi-level tunability, opening promising opportunities for high-speed, energy-efficient, and brain-inspired spintronic devices.

**Response to the Report of Reviewer A**

**Reviewer A's General Comment:** In the previous review, I raised two concerns: (1) an insufficient/incorrect explanation of the neural network simulation and (2) the use of skyrmion devices only in a part of the network. Regarding comment #1, the revised manuscript explains the neural network simulation procedure well and meets the required standard.

**Authors' Reply:** We sincerely thank the reviewer for the careful reading of our manuscript and for pointing out that our revised manuscript provides a clear and sufficiently detailed explanation of the neural network simulation procedure.

**Comment #1:** However, regarding comment #2, I think that the authors' response is still insufficient to demonstrate that the impact of this paper is sufficient for publication in Nature Communications. While the authors mention that the fully connected layers play a more critical role in the network, the convolutional layers are also essential for the CIFAR-10 task and therefore also play a critical role; without convolutional layers, the CIFAR-10 task cannot be solved. Therefore, the reason why the fully connected layers are treated specially (i.e., why the skyrmion device is used only in the fully connected layers) is still unclear. For example, if both convolutional and fully connected layers are implemented using a conventional architecture, and if the energy consumption of the fully connected layers is much larger than that of the convolutional layers, then the proposed hardware architecture would be meaningful. However, without such a discussion, I cannot evaluate the importance or significance of the proposed "hybrid" skyrmion-based optical neuromorphic computing device.

**Authors' Reply:** We sincerely appreciate the reviewer's valuable comments, which are of great significance to improve the clarity of our manuscript regarding the neural network simulation. We agree with the reviewer that both the convolutional layers and the fully connected layers are essential for successfully performing the CIFAR-10 task. To address the reviewer's concern, we have conducted simulations in which both the convolutional and fully connected layers are implemented using the proposed skyrmion-based device, rather than adopting a "hybrid" architecture. The corresponding results are presented in **Figs. R1a-c**. As shown in **Fig. R1a**, the confusion matrix after a single training epoch, reflecting the network's initial untrained state with substantial classification errors across all categories. In contrast, after 100 epochs, the matrix evolves into a sharp diagonal pattern (**Fig. R1b**), indicating effective

learning and accurate class discrimination, with most predictions correctly matching the true labels. The system achieves an average training accuracy of ~91% (**Fig. R1c**), which is comparable to that of the "hybrid" configuration presented in our previous manuscript (~92%). The slight reduction in accuracy is attributed to additional non-idealities introduced when the convolutional layers are also implemented using the skyrmion device for feature extraction. These results demonstrate that the device framework is not limited to fully connected layers, but can be extended to convolutional operations without significantly compromising overall performance.

In the revised manuscript, the previous **Figs. 5d-f** in the main text have been replaced with **Figs. R1a-c** and the corresponding description has been added in the manuscript (Page 15, Lines 440-442 and Page 16, Lines 464-466 in the main text).

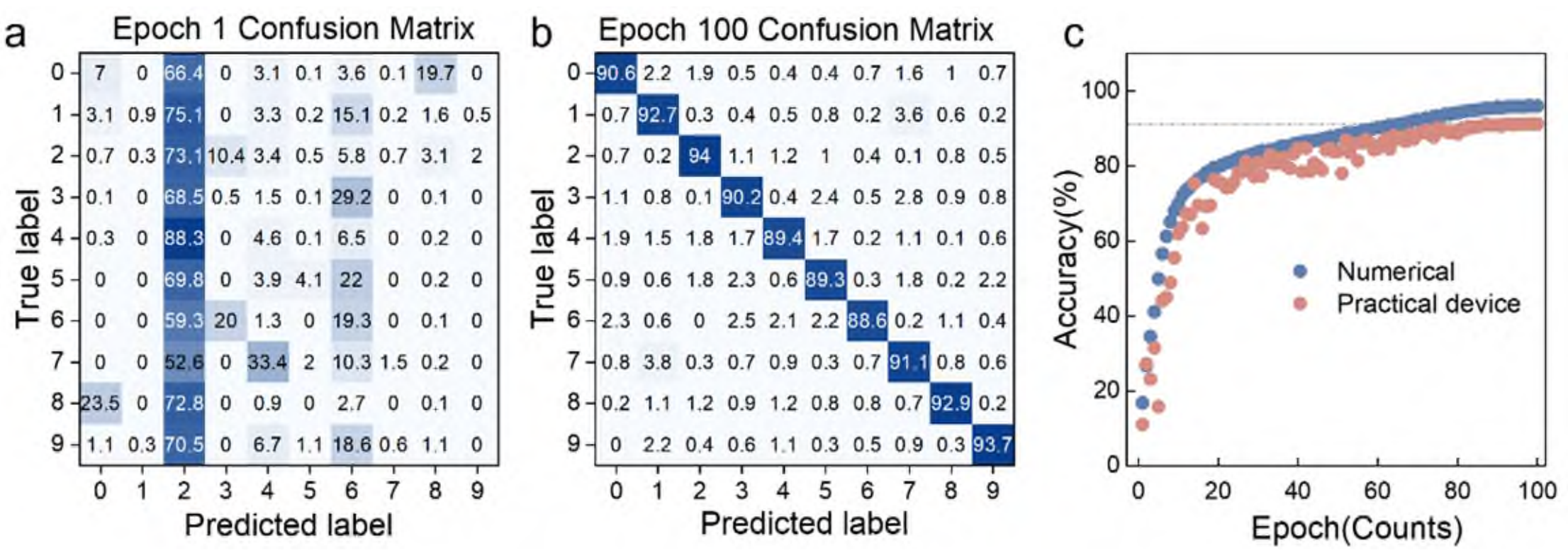


**Figure R1.** Network structure of VGG network-16 for CIFAR-10 image classification. Confusion matrix of predicted and actual values for image recognition task, **a** before and **b** after training for 100 epochs. **c** Comparison of recognition accuracy between the device and the ideal network.

**Reviewer A's Comment #2:** In addition, in the abstract, the authors state "skyrmion-based optical neuromorphic computing with a simulated training accuracy of ~92%". However, as described above, since this accuracy is obtained from a simulation of a "hybrid" hardware system (with convolutional layers implemented by a conventional architecture and fully connected layers by a skyrmion device), this description may be misleading.

**Authors' Reply:** We sincerely appreciate the reviewer's valuable comments, which have helped improve the quality and clarity of our manuscript. Our updated simulations demonstrate that both the convolutional and fully connected layers can be successfully implemented using skyrmion-based optical neuromorphic computing devices, while maintaining a classification accuracy of 91%. Accordingly, we have revised the

description in the abstract as “skyrmion-based optical neuromorphic computing with a simulated training accuracy of ~91%” (Page 2, Lines 47-49 in the main text).

**Reviewer A’s Comment #3:** In summary, I find that the advantage or significance of the high CIFAR-10 accuracy (92 %) achieved by the present skyrmion-based optical neuromorphic computing system is not sufficiently clear. Given that this high accuracy is one of the main results of the study, as highlighted in the abstract, the authors should provide a more rigorous justification before publication.

**Authors’ Reply:** We sincerely appreciate the reviewer’s valuable comments, which are of great significance to improve the quality and clarity of our manuscript. In the revised manuscript, we have conducted additional simulations in which both the convolutional and fully connected layers are implemented using the proposed skyrmion-based device, rather than adopting a “hybrid” architecture. The system achieves a training accuracy of ~91% (**Fig. R1c**), comparable to that of the “hybrid” configuration. These results demonstrate that the proposed device framework is not limited to fully connected layers, but can be extended to convolutional operations without significantly compromising overall performance, thus underscoring its practical feasibility and significance.

In the revised manuscript, the previous **Figs. 5d-f** have been replaced with **Figs. R1a-c** and the corresponding description has been added in the manuscript (Page 15, Lines 440-442 and Page 16, Lines 464-466 in the main text).

**Response to the Report of Reviewer B**

**Reviewer B's General Comment:** The authors have addressed the concerns raised in the review carefully. However, from the manuscript, it has become clear that the synthesis of this material is quite specific and non-trivial. In the literature, some reports describe Fe-enriched $Fe_{3+x}GaTe_2$ compounds, whereas the present work appears to involve Fe-deficient compositions with Pd intercalation. Because of this difference, it is important that the synthesis procedure be described in much greater detail before publication. In particular, the authors should provide a detailed description of the synthesis of the Pd-intercalated compounds in the Supporting Information. The current description of the synthesis is very general, and it is likely that certain specific steps or experimental strategies were necessary to successfully obtain these materials. The authors mention that they attempted to substitute or dope other elements but were unsuccessful; this further suggests that the synthesis conditions are quite sensitive and therefore should be clearly documented.

**Authors' Reply:** We sincerely appreciate the reviewer's valuable comments, which are of great significance to improve the clarity of our manuscript regarding the crystal synthesis details. In response, we have provided a comprehensive description of the synthesis procedure for the Pd-intercalated $Fe_{3-\delta}GaTe_2$, as summarized below.

Pd-intercalated $Fe_{3-\delta}GaTe_2$ crystals were grown using a Te-flux method. High-purity Fe (99.99%), Pd (99.99%), Ga (99.99%), and Te (99.99%) powders were mixed in molar ratio 1: x : 1 : 2 (x = 0, 0.05, 0.1, 0.2). To prevent oxidation, all weighing and mixing processes were carried out in an argon-filled glovebox. The mixture was then loaded into an alumina crucible, which was subsequently enclosed in a quartz tube and sealed under high vacuum. Prior to sealing, the quartz tube was purged twice under low vacuum (<10 Pa) and once under high vacuum ($10^{-4}$ Pa). The sealed ampoule was placed in a muffle furnace, with its position close to the furnace thermocouple to ensure accurate temperature control. The ampoule was heated to 1273 K over 10 hours and held for 24 hours. During the holding stage, the ampoule was gently shaken to promote melt homogeneity. The temperature was then reduced to 1153 K within 1 hour, followed by slow cooling to 1033 K over 120 hours, and subsequently maintained at this temperature for an additional 167 hours. Finally, the excess molten flux was

removed by centrifugation to obtain $Fe_{3-\delta}GaTe_2Pd_x$ crystals. During centrifugation, the sample undergoes natural cooling, enabling the formation of a thermodynamically stable phase.

We note that the synthesis is highly sensitive to the Fe : Ga : Te ratio, which plays a critical role in stabilizing the $Fe_{3-\delta}GaTe_2$ phase. Specifically, when the molar ratio of Fe : Ga : Te is set to x : 1 : 2 with x = 0.9-1.2, the $Fe_{3-\delta}GaTe_2$ phase is preferentially obtained. In contrast, lower Fe content (x = 0.6-0.8) leads to the formation of Ga-Te secondary phases, whereas higher Fe content (x = 1.3) favors Fe-Te compounds [*Nat. Commun.* 15, 1017 (2024)]. Based on this phase stability window, we selected a Fe : Ga : Te ratio of 1 : 1 : 2 as the starting composition for Pd intercalation.

In addition, because stabilization of the $Fe_{3-\delta}GaTe_2$ phase is highly sensitive to the chemical composition, melt homogeneity is also important for successful phase formation. Accordingly, the ampoule was gently shaken during the holding stage at 1273 K to improve melt uniformity and promote the formation of the target phase. Importantly, this thermal-equilibrium growth route differs from previously reported Fe-enriched $Fe_{3+x}GaTe_2$ systems [*Nat. Commun.* 15, 3828 (2024)], which are typically obtained via rapid quenching. In contrast, our slow-cooling process promotes the formation of the thermodynamically stable Fe-deficient phase, which is beneficial for achieving Pd intercalation.

In the revised manuscript, these additional details have been added into **Methods** (Page 17, Lines 508-517, Page 18, Lines 518-519 in the main text) to ensure reproducibility and to clarify the distinctions from previously reported synthesis approaches.

**Reviewer B's Comment #2:** Although EDX data are provided, the authors have not included XRD results for the Pd-intercalated compounds. Structural characterization, such as XRD, should be included to confirm phase formation.

**Authors' Reply:** We sincerely appreciate the reviewer's valuable comments, which are of great significance to improve the quality of our manuscript. In the previous version of the manuscript, we performed atomic-resolution scanning transmission electron microscopy (STEM) along the [100] and [001] zone axes on $Fe_{3-\delta}GaTe_2Pd_{0.05}$ to investigate the structural effect of Pd intercalation. The STEM analysis indicates that Pd atoms are intercalated at the III sites within the vdW gaps, in good agreement with

our first-principles calculations (as illustrated in **Figs. R1a** and **b**, **Supplementary Note 2**). Moreover, the measured lattice parameters ($a = b = 4.05$ Å, $c = 16.15$ Å) are very close to those of pristine $Fe_{3-\delta}GaTe_2$ ($a = b = 4.08$ Å, $c = 16.13$ Å) [*Nat. Commun.* 15, 1017 (2024)]. Specifically, the in-plane lattice constants (*a* and *b*) show only a contraction of ~0.03 Å, while the out-of-plane lattice constant (*c*) exhibits a slight expansion of ~0.02 Å. Given the low concentration of intercalated Pd atoms (below 1% for $Fe_{3-\delta}GaTe_2Pd_{0.05}$), no global crystallographic symmetry change is expected. Accordingly, the pristine $P3m1$ space group of $Fe_{3-\delta}GaTe_2$ is preserved, while Pd intercalation mainly induces subtle local structural distortions.

Based on the reviewer's comments, we subsequently performed X-ray diffraction (XRD) measurements on the cleaved (001) surface, as schematically illustrated in **Figs. R1c** and **e**. After Pd intercalation, no additional diffraction peaks were observed along the (00L) direction, while the strongest Bragg peaks ($2\theta = 33.4°$) exhibited a slight shift of ~0.05° towards lower angles. This behavior is consistent with the XRD patterns simulated on the basis of the STEM-derived structural model (**Figs. R1d** and **f**), further confirming the reliability of the structural model established from the STEM results. However, when single-crystal XRD measurements were carried out on these Pd-intercalated samples, diffraction patterns comparable to those of pristine $Fe_{3-\delta}GaTe_2$ could not be obtained, indicating a reduction in single-crystal quality after Pd incorporation. This can be attributed to the fact that, although the crystals remain globally oriented along the (001) direction, in-plane grain boundaries are likely introduced within the basal plane.

In the revised manuscript, the XRD results and associated descriptions have been added into the Supporting Information (Page 7, Lines 189-190 in the main text and **Supplementary Figure S12**).

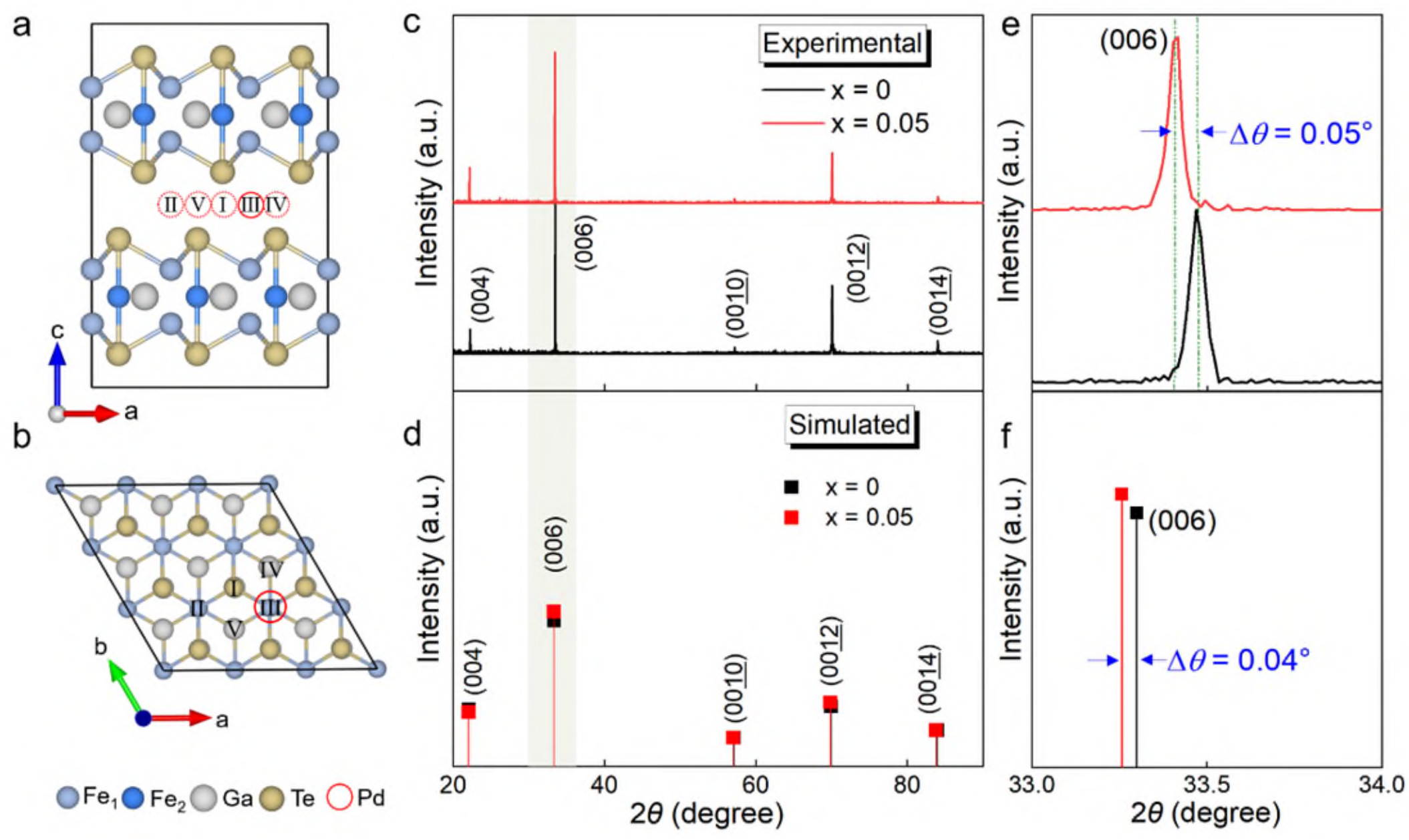


**Figure R1. a**, **b** Five Pd candidate positions (labeled as I, II, III, IV, and V) are illustrated from the perspectives of the *ac* and *ab* planes, respectively. **c, d** Experimental and simulated X-ray diffraction spectra for $Fe_{3-\delta}GaTe_2$ and $Fe_{3-\delta}GaTe_2Pd_{0.05}$. **e, f** An enlarged view of the X-ray diffraction spectrum highlighting the (006) diffraction peak.

**Reviewer B's Comment #3:** Additionally, the authors should clarify how Ga was retained in the sample during synthesis and whether any specific strategy was employed to successfully achieve Pd intercalation.

**Authors' Reply:** We sincerely appreciate the reviewer's valuable comments, which are of great significance to improve the quality of our manuscript. We found that the retention of Ga in $Fe_{3-\delta}GaTe_2$ is highly sensitive to the Fe content during synthesis. Specifically, when the molar ratio of Fe : Ga : Te was set to be x : 1 : 2 with x = 0.9-1.2, Ga was effectively retained and the $Fe_{3-\delta}GaTe_2$ phase was preferentially obtained. In contrast, lower Fe content (x = 0.6-0.8) led to the formation of Ga-Te phases, whereas higher Fe content (x = 1.3) favored Fe-Te phases [*Nat. Commun.* 15, 1017 (2024)]. Based on this phase stability window, we selected a Fe : Ga : Te ratio of 1 : 1 : 2 as the starting composition for Pd intercalation.

We further note that, because stabilization of the $Fe_{3-\delta}GaTe_2$ phase is highly sensitive to the chemical composition, melt homogeneity is important for successful phase formation. Accordingly, the ampoule was gently shaken during the holding stage at

1273 K to improve melt uniformity and promote the formation of the target phase. Importantly, this thermal-equilibrium growth route differs from previously reported Fe-enriched $Fe_{3+x}GaTe_2$ systems, which are typically obtained via rapid quenching. In contrast, our slow-cooling process promotes the formation of the thermodynamically stable Fe-deficient phase, which is beneficial for achieving Pd intercalation.

Nevertheless, despite of these efforts, it is difficult to completely eliminate the formation of Ga-Te or Fe-Te compounds. Thus, several characterization techniques, including energy dispersive X-ray (EDX) spectroscopy, magnetic measurements, and X-ray diffraction (XRD) analysis should be employed to distinguish between the flakes of $Fe_{3-\delta}GaTe_2Pd_x$ and Ga-Te or Fe-Te compounds, ensuring that only the desired material is used for subsequent measurements.

**Reviewer B's Comment #4:** The authors also show DFT calculations indicating that the Pd-intercalated structure is energetically more stable than the non-intercalated compound. However, it would be helpful if the authors could provide further discussion on the underlying chemistry that allows Pd to intercalate successfully. Why does Pd work in this structure, while other attempted substituents do not? Clarifying the chemical or electronic factors that determine the successful incorporation of Pd would significantly strengthen the manuscript.

**Authors' Reply:** We thank the reviewer for the constructive comments. Following the reviewer's suggestion, we have further clarified the mechanism of atomic intercalation into $Fe_{3-\delta}GaTe_2$ and explained why Pd is preferentially intercalated compared with other heavy transition metals (HTMs, HTMs=Zr, Hf, Nb, Ta, Mo, W, Ir, Pt, and Rh). The intercalation of HTM atoms into $Fe_{3-\delta}GaTe_2$ involves three steps: dissociation, migration, and stabilization, as detailed below:

The first step involves the dissociation of the HTM materials into isolated atoms, which determines the availability of atomic species for subsequent intercalation. To characterize this process, we evaluated the cohesive energies of various HTMs, which represent the energy required to separate bulk metals into isolated atoms, as shown in **Table R1** [*J. Chem. Educ.* 89, 592 (2012)]. The results show that Pd has the lowest

cohesive energy among the considered HTM elements, indicating that it can more readily dissociate into isolated atoms, thereby facilitating subsequent intercalation into the vdW gaps.

*[REDACTED]*

**Table R1.** Cohesive energy of HTM atoms (Zr, Hf, Nb, Ta, Mo, W, Ir, Pt, Rh, Pd) [*J. Chem. Educ.* 89, 592 (2012)].

Then, the dissociated atoms migrate within the vdW gaps of FGT, where the migration energy barrier characterizes the kinetic accessibility of interlayer transport. **Fig. R2a** shows the calculated diffusion pathways of HTM atoms within the vdW gap, with the corresponding energy profiles in **Fig. R2b**. The energy of the metal atom at the 4-site configuration was set to zero as the reference state. Further computational details are provided in **Supplementary Note 2**. The results show that Pd exhibits the lowest migration barrier among all investigated elements, indicating enhanced interlayer diffusivity and a stronger tendency to occupy energetically favorable intercalation sites, consistent with its relatively small atomic radius.

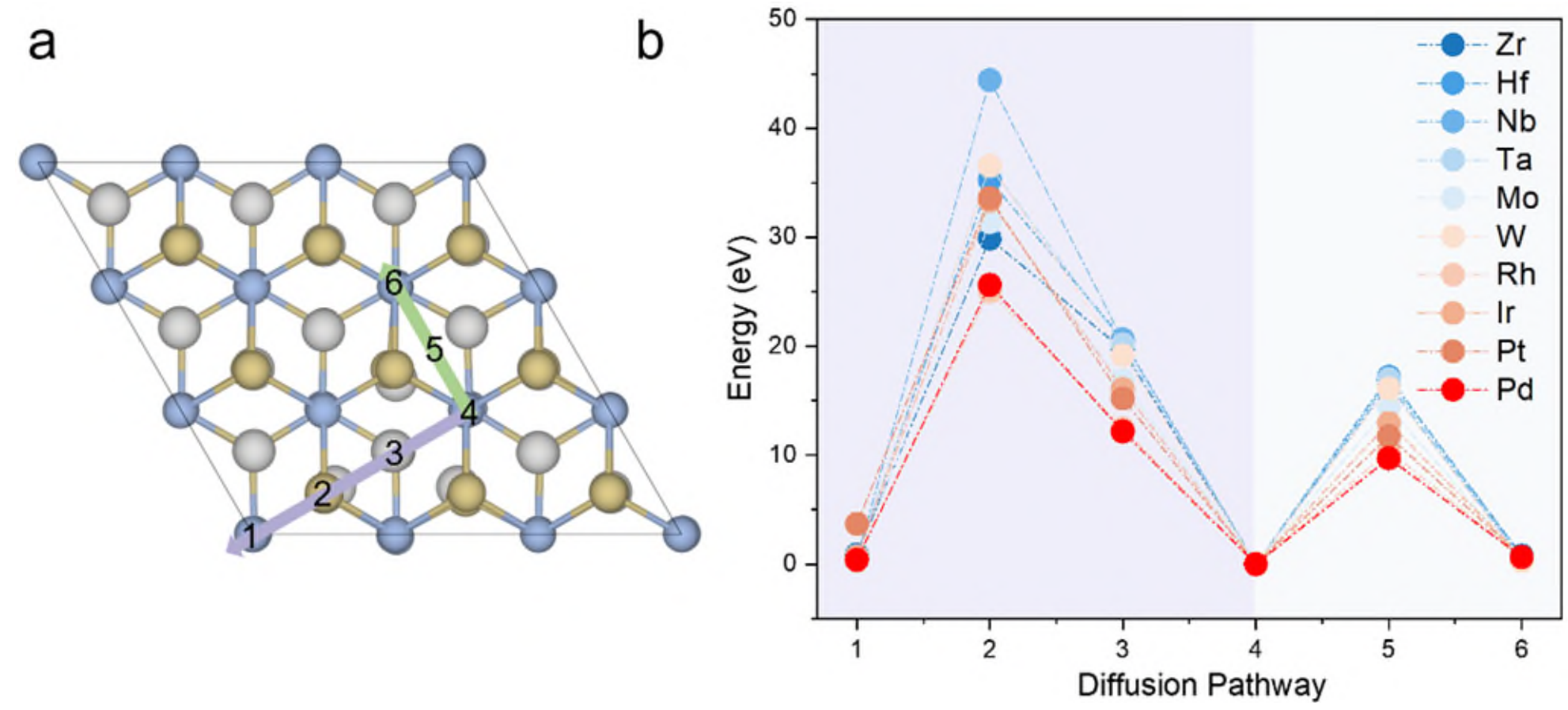


**Figure R2. Migration energy of various heavy transition metal atoms in the vdW gap of $Fe_{2.88}GaTe_2$. a**, Diffusion pathway of heavy metal atoms (Zr, Hf, Nb, Ta, Mo, W, Ir, Pt, Rh, Pd) within the vdW gap of FGT and **b**, the corresponding energy profile. The energy of the metal atom at the 4-site configuration is set to zero as the baseline reference with GGA functionals.

Following migration within the vdW gaps, atoms stabilize at energetically favorable interlayer sites, which determines the thermodynamic stability of the intercalated system. To quantify this stability, we calculated the formation energies of various HTMs

within intercalated systems, as depicted in **Fig. R3a**. The formation energy is defined as:

$$E_f = E_{Intercalated\text{-}FGT} - (E_{FGT} + E_{Intercalated\text{-}atom}), \quad (1)$$

where $E_{Intercalated\text{-}FGT}$, $E_{FGT}$, and $E_{Intercalated\text{-}atom}$ denote the energy of the intercalated system, pristine $Fe_{2.88}GaTe_2$, and the isolated intercalated atom, respectively. Our results show that Pd exhibits the lowest formation energy (-5.16 eV/unit cell), as shown in **Fig. R3b,** significantly lower than the others, indicating that Pd intercalation is the most thermodynamically favorable.

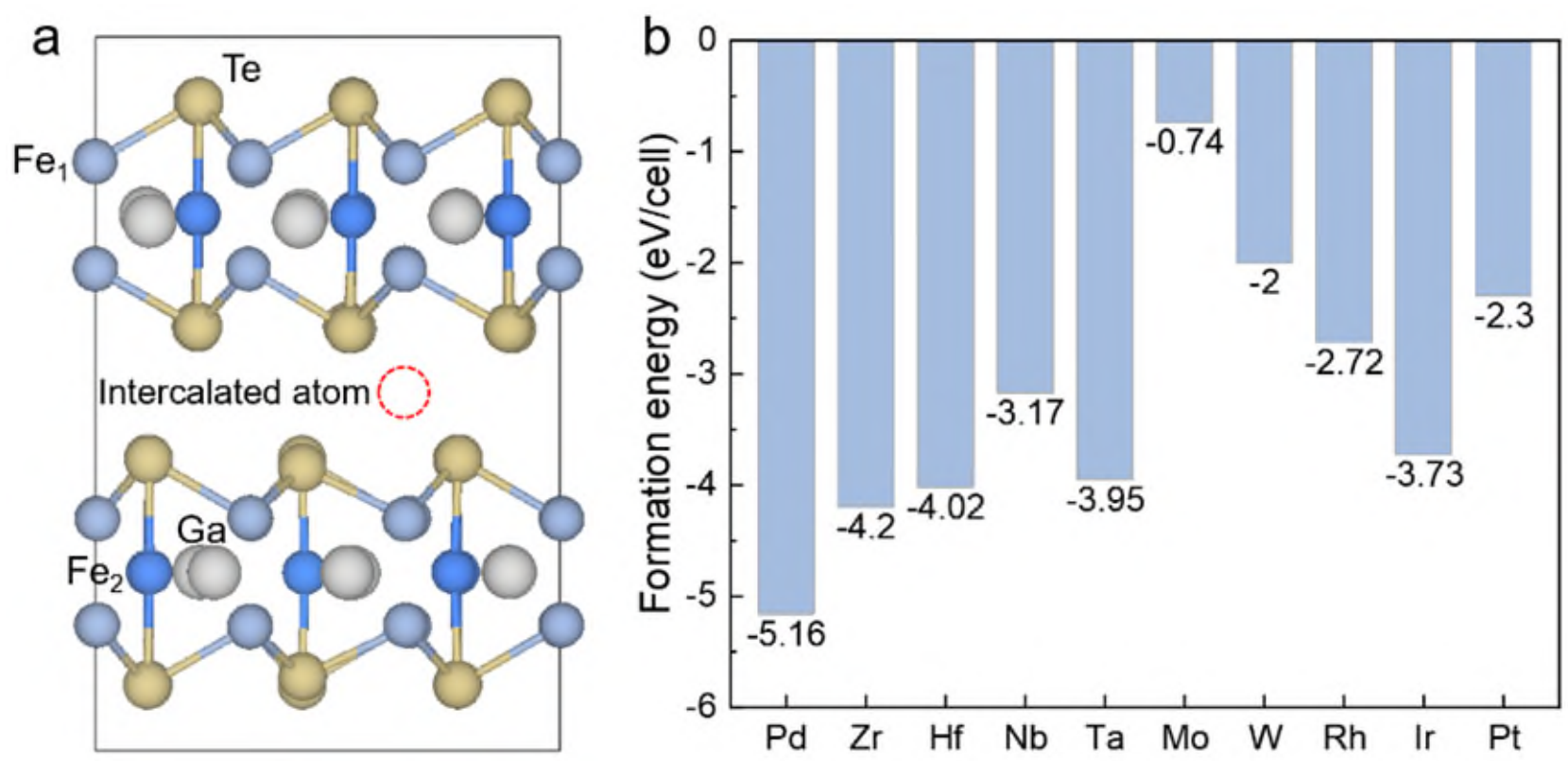


**Figure R3. a,** Structure configuration of intercalated atoms in the gaps of the $Fe_{2.88}GaTe_2$. **b,** The calculated formation energies of heavy metal atoms (Zr, Hf, Nb, Ta, Mo, W, Ir, Pt, Rh, Pd)-intercalated gaps of the $Fe_{2.88}GaTe_2$.

In summary, the lower cohesive energy, migration energy, and formation energy of Pd collectively explain its preferential intercalation into $Fe_{3-\delta}GaTe_2$, whereas other HTM elements are less favorable under similar synthesis conditions. The corresponding discussion has been added in the manuscript (Page 6, Lines 173-175 in the main text) and **Supplementary Note 2**.

**Reviewer B's Comment #5:** Providing these experimental and conceptual details is important to ensure that the synthesis can be reproduced by other researchers in the future. Once these points are addressed and the synthesis procedure is described in sufficient detail in the Supporting Information, I believe the manuscript would be suitable for publication.

**Reply:** We sincerely thank the reviewer for the constructive suggestions. Following the reviewer's comments and suggestions, we have incorporated detailed synthesis

procedures, XRD characterization results, and DFT calculations into the revised manuscript and Supporting Information (Supplementary **Table S3**, **Figure S9, S12**). We believe these additions substantially improve the reproducibility of the work, and we hope they fully address the reviewer’s concerns.

**Response to the Report of Reviewer B**

**Reviewer B's General Comment:** The explanations are satisfactory overall, and the manuscript can be accepted after minor clarification.

**Authors' Reply:** We sincerely thank the reviewer for the careful reading of our manuscript and for pointing out that our explanations are satisfactory overall. In response to the remaining request, the relevant text has been carefully revised in the manuscript. We hope that the revised manuscript will addresses the reviewer's concern.

**Comment #1:** However, regarding the synthesis procedure, there appears to be a possible inconsistency in the authors' responses. In response to Comment 1, the authors mentioned that the liquid flux was removed by centrifugation. Later, in response to Comment 3, they stated that flakes must be carefully selected because secondary phases such as GaTe and FeTe are also formed along with $Fe_3GaTe_2$. These statements appear somewhat contradictory.

**Authors' Reply:** We sincerely appreciate the reviewer's valuable comments. We agree that the descriptions in our previous responses could have been clearer, and we have now added further clarifications to avoid possible misunderstanding.

To illustrate the crystal growth process more clearly, we have presented a schematic diagram of the synthesis procedure, as shown in **Fig. R1**. In this process, stoichiometric starting materials with nominal compositions of Fe : Ga : Te : Pd = 1 : x : 1 : 2, where x = 0, 0.05, 0.1, and 0.2, were first loaded into the bottom of an alumina crucible (Step **I**). A filter was placed on top of the crucible to separate the crystals from the liquid flux during centrifugation. To facilitate the drainage of the liquid flux, an alumina tube was further placed above the filter.

The entire assembly was then sealed in an evacuated quartz ampoule for heat treatment. Upon heating to 1273 K, the starting materials were dissolved in the molten flux, forming a liquid phase (Step **II**). After a series of controlled temperature steps for crystal growth (Step **III**), solid crystalline products gradually formed within the flux. Centrifugation was then performed at 1033 K to remove the remaining liquid flux from these crystals (Step **IV**). During centrifugation, the ampoule was inverted, allowing the residual liquid flux, mainly containing Ga and Te, to be expelled through the filter into the opposite end of the ampoule. It should be emphasized that centrifugation removes the residual liquid flux, but it does not remove the crystals that have formed during

growth process. According to the Ga-Te phase diagram, the centrifugation temperature of 1033 K is slightly below the formation temperature of Ga-Te binary phases ($T_{Ga–Te}$ ~ 1049 K) [*Calphad* **16**, 317 (1992)]. Therefore, before centrifugation, Ga-Te secondary phases have already formed together with the target $Fe_{3-δ}GaTe_2Pd_x$ crystals in the crucible. After centrifugation, these solid phases were retained in the crucible, while only the liquid flux was removed.

In our previous responses, the statement that "the liquid flux was removed by centrifugation" refers to the removal of the residual liquid flux, mainly containing Ga and Te. In contrast, the statement that "flakes must be carefully selected" refers to the subsequent selection of target $Fe_{3-δ}GaTe_2Pd_x$ crystals from the retained solid crystalline products, which contain Ga-Te secondary phases that had already formed during the crystal growth process before centrifugation. These clarifications have been added to the **Methods** (Page 18, Lines 520-530) section of the revised manuscript to avoid possible misunderstanding by readers.

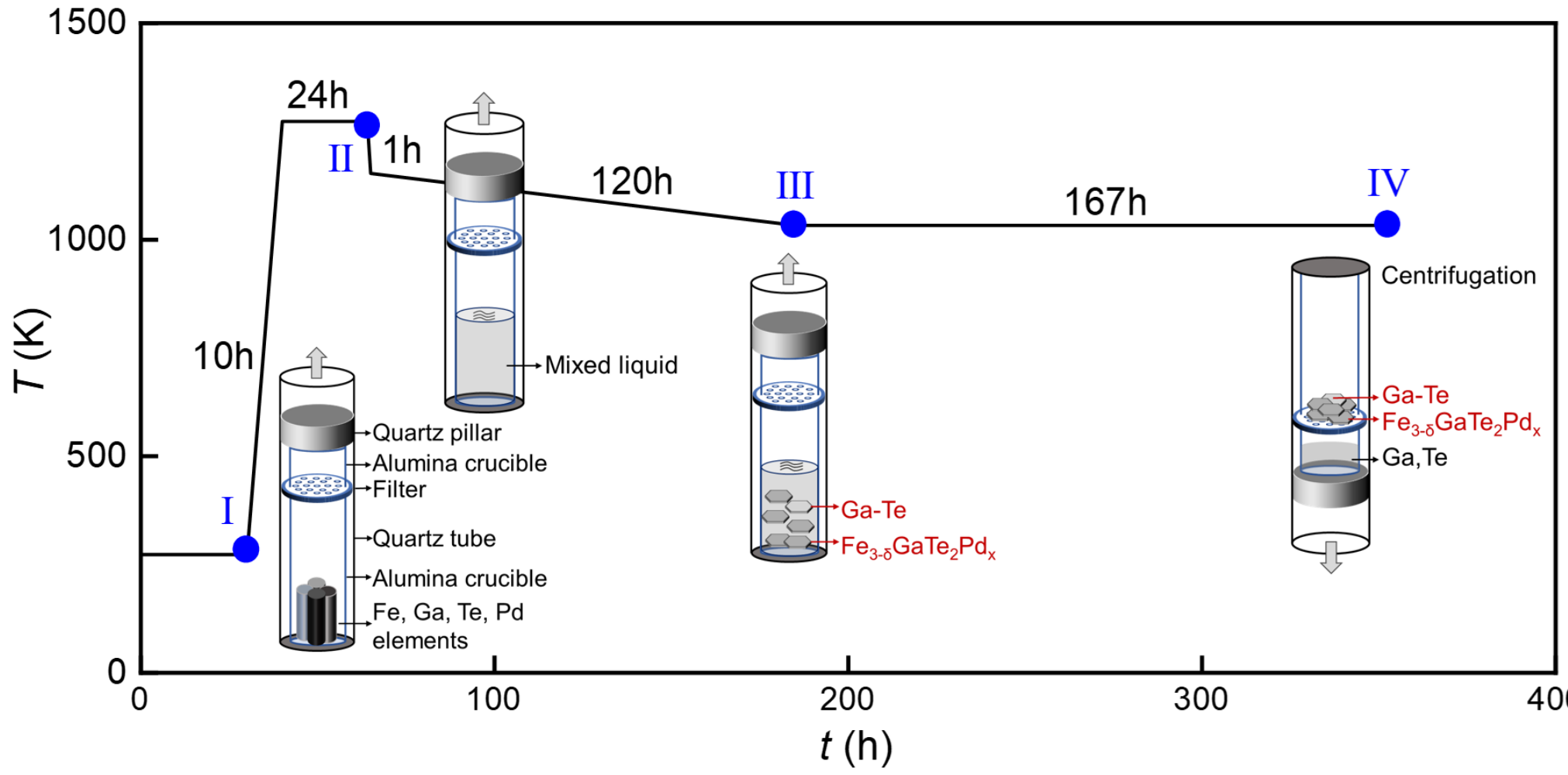


**Figure R1.** The temperature-time dependent plot for single-crystal growth. **I-IV** Schematic diagram illustrating the entire single-crystal growth.

**Comment #2:** The authors also mentioned that the crystallinity of the synthesized material is lower than that of the pristine parent compound, possibly due to the introduction of grain boundaries within the planes. Based on this explanation, it may also be inferred that the presence of such grain boundaries could hinder the growth of large single crystals. If so, this synthesis approach may face limitations in scalability for obtaining sizable, high-quality single crystals.

**Authors' Reply:** We sincerely thank the reviewer for this insightful comment. To address the reviewer's concern, we have statistically analyzed the sizes of both the $Fe_{3-}$

$_{\delta}GaTe_2Pd_x$ and pristine $Fe_{3-\delta}GaTe_2$ crystals. **Fig. R2** presents representative optical images of the corresponding crystals. It can be clearly seen that the typical lateral size of the $Fe_{3-\delta}GaTe_2Pd_x$ crystals is on the order of 0.5-1 mm, which is approximately half that of the pristine $Fe_{3-\delta}GaTe_2$ (1-2 mm) crystals. This result suggests that Pd intercalation indeed imposes constraints on the growth of sizable crystals, consistent with the reviewer's consideration.

Nevertheless, the obtained $Fe_{3-\delta}GaTe_2Pd_x$ crystals are sufficiently large for the magnetic transport and Lorentz transmission electron microscopy (LTEM) characterizations performed in this work. Therefore, although our synthesis approach may currently face limitations in scaling up to large crystals, it remains sufficient for establishing the Pd-intercalation-induced modification of magnetic interactions and skyrmion properties discussed in this manuscript.

In the revised manuscript, we have added relevant clarifications to avoid overstating the scalability of the synthesis approach (Page 18, Lines 518-519 in the main text, Supplementary **Fig. S12**).

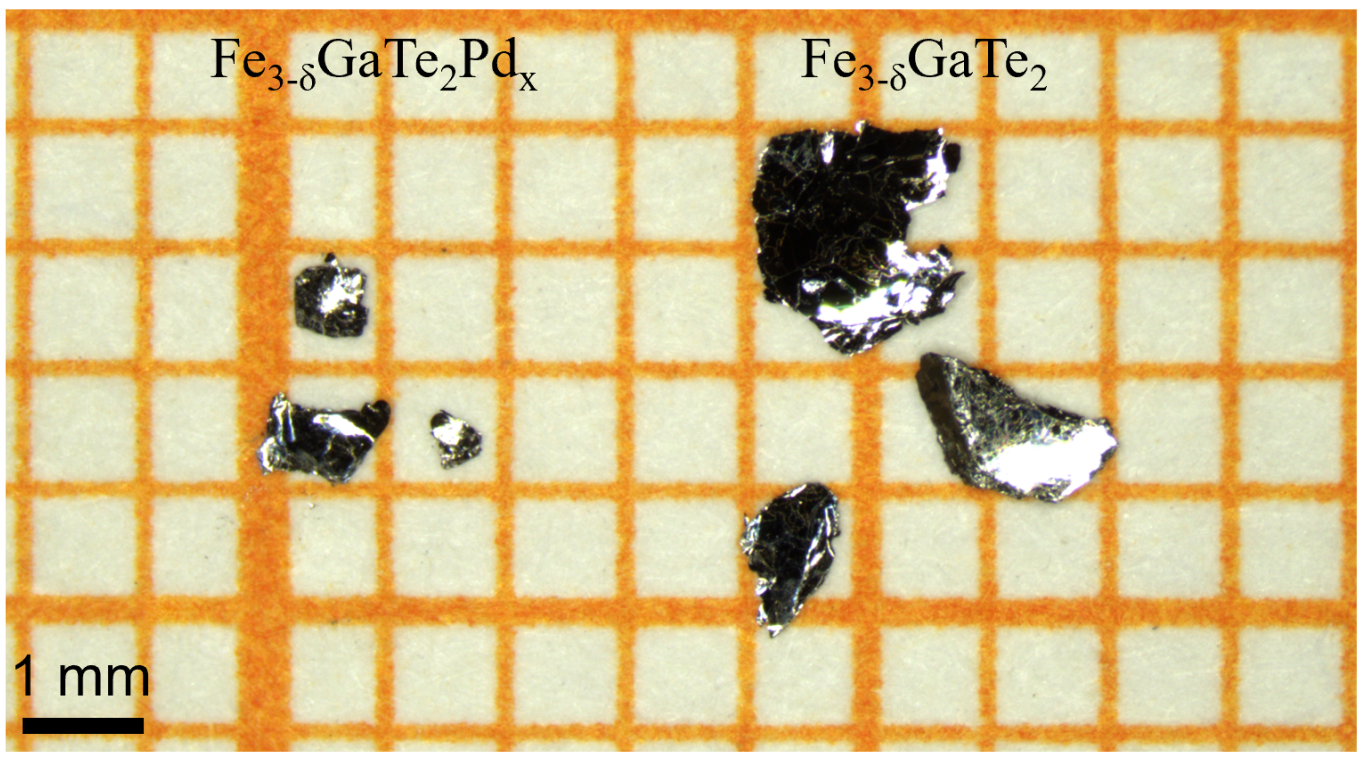


**Figure R2.** Optical images of typical $Fe_{3-\delta}GaTe_2Pd_x$ and $Fe_{3-\delta}GaTe_2$ crystals. The scale bar is 1 mm.

**Comment #3:** Therefore, it would be helpful if the authors could clarify how effectively the molten flux is removed after centrifugation, whether residual flux contributes to the formation of GaTe and FeTe secondary phases, the approximate fraction or extent of these impurity phases, and whether the reduced crystallinity and grain-boundary formation limit the growth of large single crystals.

**Authors' Reply:** We sincerely thank the reviewer for these valuable comments, which have helped us improve the clarity of the manuscript. As detailed in our response to Comment #1, the molten flux, mainly consisting of Ga and Te, was effectively

separated from the solid crystalline products during centrifugation. In this process, the residual liquid flux passed through the filter and was transferred to the opposite end of the quartz ampoule, thus separating it from the retained crystalline products used for subsequent characterizations. We would like to emphasize that the Ga-Te secondary phases discussed here are not formed from residual flux remaining after centrifugation, but rather originate from solid crystalline phases that had already formed during the crystal growth process before centrifugation.

To evaluate the extent of these secondary phases, we performed energy-dispersive X-ray spectroscopy (EDX) measurements on 23 randomly selected crystals collected from the retained solid products. The results indicate that approximately 70% of the crystals correspond to the target $Fe_{3-\delta}GaTe_2Pd_x$ phase, while the remaining ~30% are Ga-Te secondary phases. Therefore, although secondary phases are present, $Fe_{3-\delta}GaTe_2Pd_x$ remains the dominant crystalline product under our growth conditions.

Regarding the crystal size, we agree with the reviewer that in-plane grain boundaries may limit the growth of large single crystals. The intercalation of Pd increases the complexity of the crystal growth process and makes it more challenging to obtain large-area crystals. As a result, the as-grown $Fe_{3-\delta}GaTe_2Pd_x$ crystals are generally smaller than pristine $Fe_{3-\delta}GaTe_2$ crystals. Nevertheless, the obtained crystal size remains sufficient for the magnetic transport and LTEM characterizations performed in this work. We have added these clarifications to the revised manuscript (Page 18, Lines 518-530 in the **Methods** and Supplementary **Fig. S12**).

**Comment #4:** It would also be useful if the authors could mention the typical crystal dimensions obtained in the synthesis. A small inset optical image of representative crystals placed on graph paper or with a scale bar would significantly help readers assess the crystal size and quality. Including these clarifications in the Supplementary Information would strengthen the manuscript further.

**Authors' Reply:** We sincerely thank the reviewer for this valuable suggestion. To address the reviewer's concern, we have taken representative optical images of the $Fe_{3-\delta}GaTe_2Pd_x$ crystals. As shown in **Fig. R2**, the typical lateral size of the $Fe_{3-\delta}GaTe_2Pd_x$ crystals is on the order of 0.5-1 mm. These optical images, together with the corresponding descriptions, have been added to the Supplementary Information as **Fig. S12** to provide readers with a clearer assessment of the crystal size and quality.
